# Health and Economic Outcomes Associated With Musculoskeletal Disorders Attributable to High Body Mass Index in 192 Countries and Territories in 2019

**DOI:** 10.1001/jamanetworkopen.2022.50674

**Published:** 2023-01-20

**Authors:** Ningjing Chen, Daniel Yee Tak Fong, Janet Yuen Ha Wong

**Affiliations:** 1 School of Nursing, Li Ka Shing Faculty of Medicine, The University of Hong Kong, Hong Kong, China; 2 School of Nursing and Health Studies, Hong Kong Metropolitan University, Hong Kong, China

## Abstract

**Importance:**

The degree to which health and economic outcomes of musculoskeletal disorders are attributable to high body mass index (BMI) has not been quantified on a global scale.

**Objective:**

To estimate global health and economic outcomes associated with musculoskeletal disorders—low back pain (LBP), gout, and osteoarthritis attributable to high BMI in 2019.

**Design, Setting, and Participants:**

This cross-sectional study used data of 192 countries and territories from the Global Burden of Diseases, Injuries, and Risk Factors Study, World Health Organization Global Health Expenditure, World Bank, and International Labour Organization databases. Data analyses were conducted from February 24 to June 16, 2022.

**Main Outcomes and Measures:**

Prevalence, years lived with disability (YLDs), health care costs, and productivity losses due to morbidity from LBP, gout, and osteoarthritis attributable to high BMI by region and country. Prevalence and YLDs were calculated with the population attributable fraction approach. The economic burden, including health care costs and productivity losses due to morbidity, was also quantified. Health care costs borne by the public, private, and out-of-pocket sectors were estimated based on their corresponding payment shares. Productivity losses were estimated based on the output per worker. A sensitivity analysis was conducted to arrive at the base, minimum, and maximum estimates (ie, uncertainty interval [UI]) by using the mean, lower, and upper bounds of all input variables.

**Results:**

High BMI was estimated to be responsible for 36.3 million (UI, 18.4-61.0 million), 16.9 million (UI, 7.5-32.5 million), and 73.0 million (UI, 32.4-131.1 million) prevalent cases of LBP, gout, and osteoarthritis, respectively, which accounted for 7.3 million (UI, 3.0-15.0 million) YLDs across 192 countries and territories in 2019. Globally, the YLDs of musculoskeletal disorders attributable to high BMI accounted for 1.0% of all-cause YLDs in the working-age population aged 15 to 84 years. The global total costs of musculoskeletal disorders attributable to high BMI reached $180.7 billion (UI, $83.8-$333.1 billion), including $60.5 billion (UI, $30.7-$100.5 billion) in health care costs and $120.2 billion (UI, $53.1-$232.7 billion) in productivity losses. In terms of the global health care costs, 58.9% ($35.6 billion; UI, $17.8-$59.6 billion) was borne by the public sector, 24.0% ($14.5 billion; UI, $7.8-$23.2 billion) by the private sector, and 17.1% ($10.3 billion; UI, $5.1-$17.6 billion) by the out-of-pocket sector. On average, the total costs accounted for 0.2% of global gross domestic product. Great inequalities in the disease and economic burden existed across regions and countries. Nearly 80% of global health care (82.4%) and morbidity-related costs (82.9%) were paid by high-income countries, whereas more than 60% (61.4%) of global YLDs occurred in middle-income countries.

**Conclusions and Relevance:**

In this cross-sectional study of 192 countries and territories, a substantial amount of the health and economic impact of musculoskeletal disorders was attributable to high BMI. Developing effective policies and active participation from health professionals to prevent excessive weight gain are needed. More available estimates are also needed to facilitate a global analysis.

## Key Points

**Question:**

What health and economic outcomes associated with musculoskeletal disorders are attributable to high body mass index (BMI) worldwide?

**Findings:**

In this cross-sectional study of 192 countries and territories, 7.3 million years lived with disability and $180.7 billion total costs associated with musculoskeletal disorders were attributable to high BMI. The disease and economic burden varied by region and country.

**Meaning:**

These results suggest that weight management should be given a paramount priority on the global health and economy agenda, and effective policies and active participation from health professionals to prevent excess weight gain are needed.

## Teaser

This cross-sectional study evaluates outcomes from musculoskeletal disorders that are attributable to high body mass index in 192 countries and territories.

## Introduction

Musculoskeletal disorders cause long-term pain, physical disability, psychological distress, and reduced quality of life.^1,2^ In 2017, the global number of prevalent cases of musculoskeletal disorders reached 1.3 billion, leading to 121.3 thousand deaths and 138.7 million disability-adjusted life years (DALYs).^3^ In addition to adverse health outcomes, musculoskeletal disorders also lead to work disability, declines in productivity, and increases in health care spending.^4^ Health care spending on musculoskeletal disorders in the US reached $380.9 billion,^5^ and health care costs combined with productivity losses due to musculoskeletal disorders cost the European region €240 billion.^4^

High body mass index (BMI; calculated as weight in kilograms divided by height in meters squared) refers to a BMI of 25 or more for individuals aged 20 years and older, and having overweight or obesity according to the International Obesity Task Force standards for children aged 1 to 19 years.^6^ In 2019, high BMI accounted for 5.0 million deaths and 160 million DALYs globally.^7^ Additionally, high BMI has cost Australia approximately $23.7 billion, including $12.0 billion in health care costs and $11.7 billion in productivity losses.^8^ The economic costs of high BMI also accounted for 9.3% of gross domestic product (GDP) in the US.^9^

High BMI has been proven to be a risk factor for low back pain (LBP), osteoarthritis, and gout among musculoskeletal disorders in the Global Burden of Diseases, Injuries, and Risk Factors Study (GBD) 2019.^6^ Such risk–outcome pairs were determined by the inclusion criteria that the association demonstrated compelling evidence (eg, the absence of confounding).^6^ Although other categories of musculoskeletal disorders (eg, neck pain) might also be associated with high BMI,^10^ the associations were supported with too weak evidence to be considered in the GBD study.^6^ Additionally, although previous studies^3,4,5,7,8,9^ have advanced our understanding of the disease and economic burden of musculoskeletal disorders and high BMI, respectively, how the burden of musculoskeletal disorders is attributable to high BMI has not been investigated. This is a major limitation because research aimed at lightening the burden of diseases by reducing exposure to risk factors often fails due to insufficient knowledge about the full range of social effects associated with these risk factors. In addition, although previous studies^4,8,9^ have shown aggregate research findings, it is necessary to understand how the economic costs are distributed among the public, private, and out-of-pocket sectors. This will provide greater insight into developing further interventions through coordinated endeavors by different sectors.

Therefore, this study aimed to investigate the health and economic burden of musculoskeletal disorders, more specifically, LBP, gout, and osteoarthritis attributable to high BMI on a global scale. In this study, we estimated economic costs, including both direct (ie, health care) and indirect (ie, productivity losses due to morbidity) costs.

## Methods

This cross-sectional study was approved by the institutional review board of the University of Hong Kong/Hospital Authority Hong Kong West Cluster and a waiver of informed consent was granted because this study used deidentified and aggregated data. This study followed the Strengthening the Reporting of Observational Studies in Epidemiology (STROBE) reporting guideline for cross-sectional studies.

### Data Sources

To our knowledge, the GBD 2019 study was the most systematic effort to measure the global epidemiological patterns of diseases, injuries, and risk factors from 1990 to 2019.^11^ We derived data on prevalence estimates and years lived with disability (YLDs) from the GBD result tool.^12^ We also extracted GDP data and the proportion of health care costs paid by sector from the World Health Organization Global Health Expenditure Database.^13^ Additionally, we obtained employment data from the World Bank and International Labour Organization (ILO) data portals.^14,15^ Moreover, labor income shares in GDP and labor force participation rates were derived from the ILO data sets.^16,17^ In total, we included 192 countries and territories with available data (eMethods and eTables 1-3 in Supplement 1).

### Statistical Analysis

#### Estimation of Disease Burden: Prevalence and YLDs

The population attributable fraction (PAF) refers to the level of morbidity that could be reduced if a risk factor is removed.^18,19^ The high BMI PAFs for the age-standardized YLD rates of LBP, gout, and osteoarthritis were extracted from the GBD 2019 database.^12^ In each disease category, we estimated the number of attributable prevalent cases by multiplying the number of prevalent cases by the PAF for the age-standardized YLD rate. We also extracted high BMI–attributable YLDs of musculoskeletal disorders by category from the GBD 2019 database.^12^ However, due to a lack of data, for patients aged 15 to 19 years with LBP or gout, and those aged 15 to 29 years with osteoarthritis, we estimated the attributable YLDs by multiplying YLDs by the PAF for the age-standardized YLD rate in each disease category (eMethods in Supplement 1). We summed these YLDs across diseases and ages as the total YLDs in each country.

#### Estimation of Health Care Costs

To our knowledge, there have been no global estimates on disease-specific health care expenditures. Therefore, we estimated the health care costs of musculoskeletal disorders attributable to high BMI in each country by extrapolating a representative estimate. We conducted a systematic review and ranked the existing national reports with several criteria. Finally, health care spending in the US was selected as a baseline result (eMethods, eFigure 1, and eTable 4 in Supplement 1).^5^

We calculated health care costs for each country in several steps. First, we calculated health care spending per case on musculoskeletal disorders by category in the US (eMethods and eTable 5 in Supplement 1). The costs per case were then extrapolated to other countries based on the country-specific spending ratio (ie, the value of health spending per head in a country divided by that in the US). We obtained health spending per head from a global health spending study.^20^ The extrapolation approach has been well applied in previous economic analyses of noncommunicable diseases.^19,21,22^ Health care costs per case estimated in our study were generally comparable with previous estimates (eMethods and eFigure 2 in Supplement 1).^23,24,25,26,27,28^ We then multiplied the number of attributable prevalent cases by health care spending per case to arrive at disease-specific health care spending. Lastly, health care costs in each country were quantified by summing across disease-specific estimates. Generally, 3 main sectors pay for health care costs worldwide. This includes the public sector (eg, funds from government), the private sector or third party (eg, funds from insurance companies), and the out-of-pocket sector. Health care costs distributed among sectors were estimated based on the spending shares.^13^

#### Estimation of Lost Output Due to Morbidity

In this study, the labor income per worker was used as a measure of productivity losses. Particularly, we calculated the labor income per head by multiplying GDP^13^ by the labor income share of GDP,^16^ and dividing by the size of the labor force.^14^ Because not all people entered the labor market, we weighted the labor income per worker by the labor force participation rate (ie, the ratio of the number of workers to the population size) in each age group.

In addition to the decreased market production, high BMI also led to a decline in nonmarket production (ie, household production), which is often excluded from the calculation of GDP. Based on previous reports, nonmarket production is equivalent to 23%^29^ and 35%^30^ of GDP in the US and Ghana, respectively. Given that most countries do not publish such data, we assumed that household production equally accounted for 23% of GDP in upper-middle–income and high-income countries, and 35% of GDP in lower-middle–income and low-income countries. We estimated morbidity-related productivity losses in the working-age population (ie, 15 years and older).^17^ Consistent with a previous study,^30^ we assumed the labor force participation rate to be zero in people aged 85 years and older. Therefore, YLDs and productivity losses were only estimated in workers aged 15 to 84 years. In each age group, we estimated productivity losses by multiplying lost production per person by YLD counts (eMethods in Supplement 1). Furthermore, productivity losses were summed across age groups and diseases. We estimated the total costs in monetary terms and as a percentage of GDP for each country and territory. Complying with the standards, we presented health care costs, productivity losses, and the total costs in US dollars to facilitate comparisons across regions and countries.

#### Sensitivity Analysis

We also conducted a sensitivity analysis to calculate the base, minimum, and maximum estimates (ie, uncertainty interval [UI]) by using the mean, lower, and upper bounds of all input variables (eTable 3 in Supplement 1). As long as the input variables were not accompanied by uncertainty intervals (eg, GDP), the values of input variables were used to calculate the minimum, base, and maximum estimates. Data analyses were conducted in RStudio Version 1.3.1093.

## Results

Globally, high BMI accounted for 7.3 million (UI, 3.0-15.0 million) YLDs in 2019. Particularly, the number of prevalent cases of LBP attributable to high BMI reached 36.3 million (UI, 18.4-61.0 million) with 4.3 million (UI, 1.9-8.1 million) YLDs (Table 1). There were also 16.9 million (UI, 7.5-32.5 million) prevalent cases with gout attributable to high BMI, leading to 0.5 million (UI, 0.2-1.0 million) YLDs. In addition, 73.0 million (UI, 32.4-131.1 million) prevalent cases with osteoarthritis were attributable to high BMI, contributing to 2.6 million (UI, 0.9-5.8 million) YLDs (Table 1). High BMI–attributable YLDs of musculoskeletal disorders accounted for 1.0% of all-cause YLDs in the working-age population aged 15 to 84 years (Figure, panel A).

**Table 1.** Disease Burden of Musculoskeletal Disorders Attributable to High Body Mass Index by World Health Organization Region in 2019

Region	Low back pain				Gout				Osteoarthritis			
	Prevalence, No. in thousands (UI)	Prevalence per 1000 persons (UI)	YLDs in thousands (UI)	YLDs per 1000 persons (UI)	Prevalence, No. in thousands (UI)	Prevalence per 1000 persons (UI)	YLDs in thousands (UI)	YLDs per 1000 persons (UI)	Prevalence, No. in thousands (UI)	Prevalence per 1000 persons (UI)	YLDs in thousands (UI)	YLDs per 1000 persons (UI)
Global	36 267 (18 437-61 011)	4.8 (2.4-8.0)	4282 (1858-8147)	0.6 (0.2-1.1)	16 947 (7455-32 458)	2.2 (1.0-4.3)	508 (214-985)	0.07 (0.03-0.13)	72 969 (32 371-131 053)	9.6 (4.2-17.2)	2556 (912-5824)	0.3 (0.1-0.8)
Africa	2760 (1375-4752)	2.5 (1.3-4.4)	306 (125-605)	0.3 (0.1-0.6)	759 (330-1485)	0.7 (0.3-1.4)	24 (9-49)	0.02 (0.01-0.05)	3965 (1814-7032)	3.6 (1.7-6.4)	142 (51-323)	0.1 (0.05-0.3)
The Americas	10 187 (5733-15 709)	10.1 (5.7-15.6)	1182 (569-2093)	1.2 (0.6-2.1)	5628 (2898-9322)	5.6 (2.9-9.2)	161 (80-279)	0.16 (0.08-0.28)	17 396 (8698-28 824)	17.2 (8.6-28.5)	614 (247-1344)	0.6 (0.2-1.3)
Eastern Mediterranean	3561 (1863-5843)	5.1 (2.7-8.4)	412 (184-773)	0.6 (0.3-1.1)	926 (431-1695)	1.3 (0.6-2.4)	29 (12-57)	0.04 (0.02-0.08)	4618 (2267-7708)	6.6 (3.3-11.1)	162 (63-359)	0.2 (0.1-0.5)
Europe	9301 (4901-15 252)	10.0 (5.3-16.4)	1120 (509-2070)	1.2 (0.5-2.2)	3077 (1406-5732)	3.3 (1.5-6.2)	89 (39-170)	0.10 (0.04-0.18)	17 248 (8339-29 273)	18.5 (9.0-31.5)	588 (230-1304)	0.6 (0.2-1.4)
Southeast Asia	4736 (2336-8245)	2.4 (1.2-4.2)	553 (225-1093)	0.3 (0.1-0.6)	1820 (741-3736)	0.9 (0.4-1.9)	58 (22-119)	0.03 (0.01-0.06)	10 031 (4241-18 469)	5.1 (2.1-9.3)	349 (116-793)	0.2 (0.1-0.4)
Western Pacific	5723 (2229-11 210)	3.0 (1.2-5.8)	708 (246-1513)	0.4 (0.1-0.8)	4737 (1649-10 488)	2.5 (0.9-5.5)	147 (52-311)	0.08 (0.03-0.16)	19 712 (7011-39 747)	10.2 (3.6-20.7)	702 (204-1701)	0.4 (0.1-0.9)

Abbreviations: UI, uncertainty interval; YLDs, years lived with disability.

**Figure.**
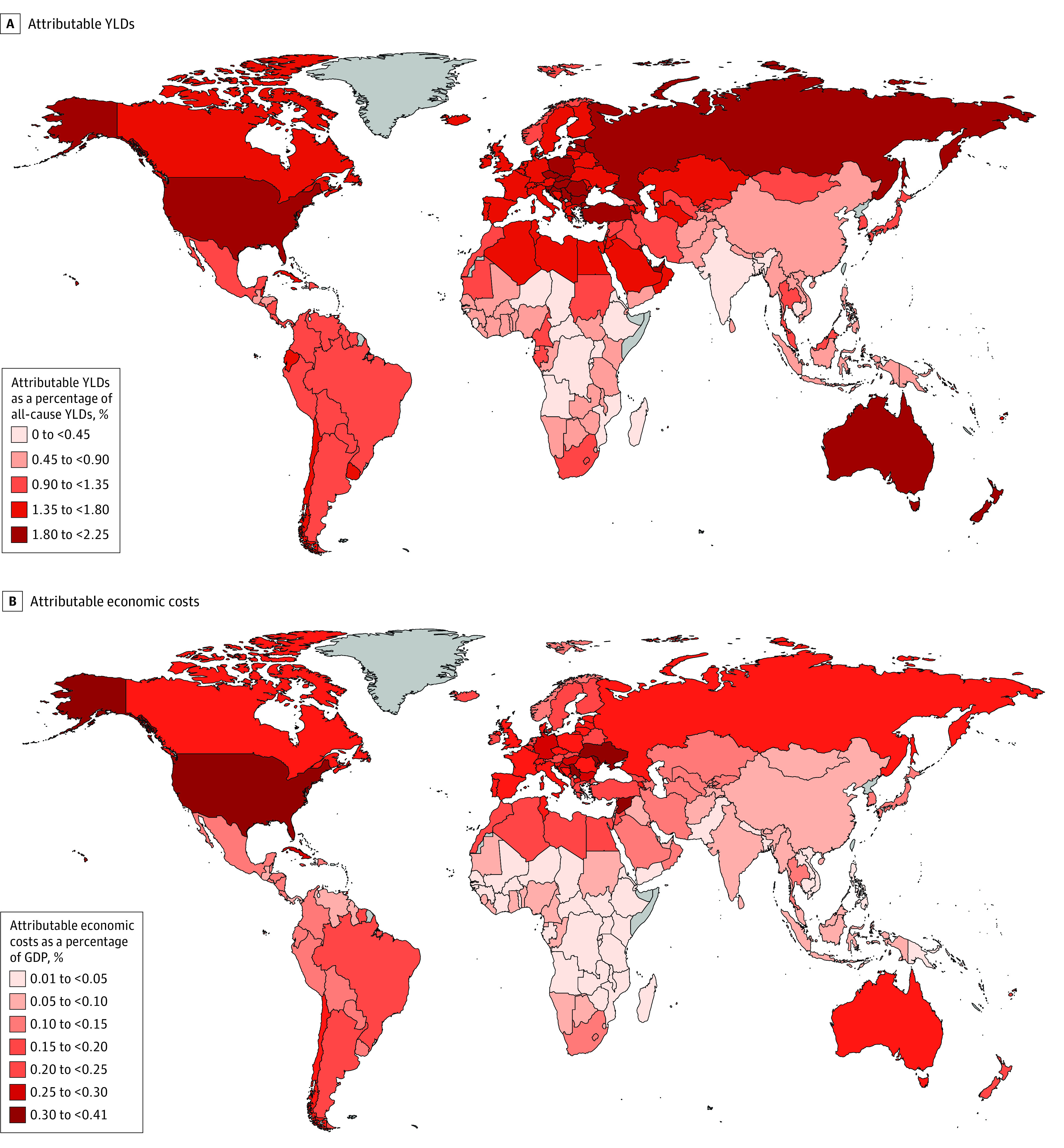
Health and Economic Burden of Musculoskeletal Disorders Attributable to High Body Mass Index Worldwide Grey areas represent countries or territories with no available data. GDP indicates gross domestic product; YLDs, years lived with disability.

At the regional level, in terms of LBP and gout, the heaviest burden was found in the Americas (10.2 million [UI, 5.7-15.7 million] prevalent cases with 1.2 million [UI, 0.6-2.1 million] YLDs, and 5.6 million [UI, 2.9-9.3 million] prevalent cases with 0.2 million [UI, 0.1-0.3 million] YLDs, respectively). In contrast, the Western Pacific region was most affected by osteoarthritis (19.7 million [UI, 7.0-39.7 million] prevalent cases and 0.7 million [UI, 0.2-1.7 million] YLDs). Africa was least affected (Table 1). Additional results are presented in eTables 6 and 7 in Supplement 1.

In 2019, high BMI accounted for $180.7 billion (UI, $83.8-$333.1 billion) of total costs worldwide (Table 2). Of these, $1.6 billion (UI, $0.7-$3.0 billion) was contributed by Africa, $98.4 billion (UI, $49.4-$171.0 billion) by the Americas, $3.9 billion (UI, $1.9-$7.0 billion) by the Eastern Mediterranean, $50.6 billion (UI, $22.6-$95.9 billion) by Europe, $3.1 billion (UI, $1.2-$6.2 billion) by Southeast Asia, and $23.1 billion (UI, $7.9-$49.9 billion) by the Western Pacific. Notably, the total costs accounted for 0.2% of global GDP on average. This percentage ranged from 0.02% in Ethiopia to 0.4% in the US. In 2019, total costs as a percentage of GDP were generally higher in high-income countries than in low-income and lower-middle–income countries. Despite this, the total costs also accounted for more than 0.3% of GDP in some low-income (eg, the Syrian Arab Republic) and lower-middle–income countries (eg, the Republic of Moldova and Ukraine) (Figure, panel B). Health care costs accounted for nearly 30% of the total costs globally, with 29.3% in Africa, 34.9% in the Americas, 37.5% in the Eastern Mediterranean, 32.2% in Europe, 27.0% in Southeast Asia, and 30.4% in the Western Pacific (Table 2). This proportion by country is shown in eTable 8 in Supplement 1.

**Table 2.** Health Care, Morbidity-Related, and Total Costs of Musculoskeletal Disorders Attributable to High Body Mass Index by World Health Organization Region in 2019

Region	Health care costs			Morbidity-related costs			Total costs	
	Amount in millions, US$ (UI)	Proportion of total, %^a^	Amount per 1000 persons, US$ (UI)	Amount in millions, US$ (UI)	Proportion of total, %^a^	Amount per 1000 persons, US$ (UI)	Amount in millions, US$ (UI)	Amount per 1000 persons, US$ (UI)
Global	60 485 (30 683-100 453)	33.5	7929 (4022-13 109)	120 233 (53 073-232 665)	66.5	15 731 (6958-30 545)	180 718 (83 757-333 118)	23 728 (10 980-43 654)
Africa	470 (242-774)	29.3	430 (221-709)	1132 (476-2269)	70.7	1037 (436-2078)	1602 (718-3043)	1467 (658-2787)
The Americas	34 376 (18 682-54 107)	34.9	34 037 (18 498-53 574)	64 049 (30 761-116 873)	65.1	63 418 (30 457-11 5847)	98 425 (49 443-170 979)	97 455 (48 956-169 315)
Eastern Mediterranean	1475 (779-2369)	37.5	2117 (1118-3400)	2454 (1107-4671)	62.5	3522 (1589-6703)	3930 (1886-7040)	5639 (2707-10 103)
Europe	16 316 (8024-27 700)	32.2	17 541 (8626-29 780)	34 278 (14 561-68 228)	67.8	36 851 (15 654-73 350)	50 594 (22 585-95 928)	54 392 (24 280-103 130)
Southeast Asia	838 (377-1510)	27.0	424 (191-764)	2265 (873-4695)	73.0	1146 (442-2376)	3103 (1249-6206)	1570 (632-3140)
Western Pacific	7011 (2579-13 993)	30.4	3645 (1341-7276)	16 055 (5296-35 929)	69.6	8348 (2754-18 682)	23 065 (7875-49 923)	11 993 (4095-25 958)

Abbreviation: UI, uncertainty interval.

^a^ Percentage calculations were based on mean values.

In 2019, $60.5 billion (UI, $30.7-$100.5 billion) health care costs of musculoskeletal disorders were attributable to high BMI globally (costs by disease category and location are provided in eTables 9 through 11 in Supplement 1). Globally, the public sector bore the largest burden (58.9%; $35.6 billion; UI, $17.8-$59.6 billion) (Table 3). Specifically, the public sector bore more than half of health care costs across the world except Southeast Asia (43.5%; $0.4 billion; UI, $0.2-$0.7 billion). Additionally, the European and Western Pacific regions were the only 2 regions wherein the private sector contributed less than 10% (8.3% and 8.1%, respectively). Furthermore, the out-of-pocket sector was responsible for 17.1% ($10.3 billion; UI, $5.1-$17.6 billion) of global health care costs. This proportion ranged from 12.9% ($4.4 billion; UI, $2.4-$7.0 billion) in the Americas to 42.2% ($0.4 billion; UI, $0.2-$0.6 billion) in Southeast Asia. Health care costs paid by sector and location are presented in eTable 12 in Supplement 1.

**Table 3.** Health Care Costs of Musculoskeletal Disorders Attributable to High Body Mass Index Borne by Sector and World Health Organization Region in 2019

Region	Public sector			Private or third-party sector			Out-of-pocket sector		
	Amount, millions US$ (UI)	Proportion of health care costs, %^a^	Amount per 1000 persons, US$ (UI)	Amount, million US$ (UI)	Proportion of health care costs, %^a^	Amount per 1000 persons, US$ (UI)	Amount, million US$ (UI)	Proportion of health care costs, %^a^	Amount per 1000 persons, US$ (UI)
Global	35 600 (17 845-59 622)	58.9	4667 (2339-7816)	14 534 (7764-23 215)	24.0	1905 (1018-3043)	10 322 (5058-17 571)	17.1	1353 (663-2303)
Africa	237 (125-384)	50.5	217 (114-352)	116 (61-191)	24.7	106 (55-175)	116 (57-198)	24.7	106 (52-181)
The Americas	17 725 (9615-27 937)	51.6	17 550 (9520-27 662)	12 176 (6661-19 074)	35.4	12 056 (6596-18 886)	4443 (2389-7048)	12.9	4399 (2366-6978)
Eastern Mediterranean	795 (423-1270)	53.9	1141 (607-1823)	192 (102-306)	13.0	275 (147-440)	488 (254-793)	33.1	701 (364-1137)
Europe	11 838 (5824-20 086)	72.6	12 727 (6261-21 594)	1362 (663-2327)	8.3	1464 (713-2502)	3118 (1538-5291)	19.1	3353 (1654-5688)
Southeast Asia	365 (165-655)	43.5	185 (83-332)	120 (54-216)	14.3	61 (27-109)	353 (158-639)	42.2	179 (80-323)
Western Pacific	4639 (1694-9289)	66.2	2412 (881-4830)	568 (223-1101)	8.1	295 (116-572)	1803 (662-3603)	25.7	937 (344-1873)

Abbreviation: UI, uncertainty interval.

^a^ Percentage calculations were based on mean values.

High BMI–associated morbidity led to productivity losses of $120.2 billion (UI, $53.1-$232.7 billion) globally. The Americas was the most affected region ($64.0 billion; UI, $30.8-$116.9 billion), followed by Europe ($34.3 billion; UI, $14.6-$68.2 billion), the Western Pacific ($16.1 billion; UI, $5.3-$35.9 billion), the Eastern Mediterranean ($2.5 billion; UI, $1.1-$4.7 billion), Southeast Asia ($2.3 billion; UI, $0.9-$4.7 billion), and Africa ($1.1 billion; UI, $0.5-$2.3 billion) (Table 2). Additional results are shown in eTable 13 in Supplement 1. The economic burden was not directly proportional to the population size and morbidity. For example, 13.2% of the world’s population is in the Americas (1.0 billion people), accounting for $34.4 billion (56.8%) of global health care costs, $64.0 billion (53.3%) of global morbidity-related costs, and 2.0 million (26.6%) of global YLDs. In contrast, Southeast Asia accounted for 2.0 billion (25.9%) of the world’s population but contributed to $838 million (1.4%) of global health care costs, $2.3 billion (1.9%) of global morbidity-related costs, and only 1.0 million (13.1%) of global YLDs. Approximately 80% of global health care (82.4%) and morbidity-related costs (82.9%) were paid by high-income countries, whereas more than 60% (61.4%) of the world’s YLDs occurred in middle-income countries (Table 4).

**Table 4.** Health Care Costs, Morbidity-Related Costs, and YLDs of Musculoskeletal Disorders Attributable to High Body Mass Index by WHO Region and World Bank Income Group, 2019

Characteristics	Population, No. in millions (proportion of global total, %)	Health-care costs, million US$ (proportion of global total, %)	Per capita health care costs, US$	Morbidity-related costs, million US$ (proportion of global total, %)	Per capita morbidity-related costs, US$	YLDs , No. in thousands (proportion of global total, %)	YLDs per 1000 persons
Global	7628 (100.0)	60 485 (100.0)	7.9	120 233 (100.0)	15.8	7346 (100.0)	1.0
WHO region							
Africa	1092 (14.3)	470 (0.8)	0.4	1132 (0.9)	1.0	472 (6.4)	0.4
The Americas	1010 (13.2)	34 376 (56.8)	34.0	64 049 (53.3)	63.4	1957 (26.6)	1.9
Eastern Mediterranean	697 (9.1)	1475 (2.4)	2.1	2454 (2.0)	3.5	603 (8.2)	0.9
Europe	930 (12.2)	16 316 (27.0)	17.5	34 278 (28.5)	36.9	1797 (24.5)	1.9
Southeast Asia	1976 (25.9)	838 (1.4)	0.4	2265 (1.9)	1.1	960 (13.1)	0.5
Western Pacific	1923 (25.2)	7011 (11.6)	3.6	16 055 (13.4)	8.3	1556 (21.2)	0.8
Income group							
High	1197 (15.7)	49 832 (82.4)	41.6	99 684 (82.9)	83.3	2640 (35.9)	2.2
Upper-middle	2895 (37.9)	9068 (15.0)	3.1	17 074 (14.2)	5.9	2991 (40.7)	1.0
Lower-middle	2909 (38.1)	1477 (2.4)	0.5	3351 (2.8)	1.2	1521 (20.7)	0.5
Low	627 (8.2)	107 (0.2)	0.2	123 (0.1)	0.2	193 (2.6)	0.3

Abbreviations: WHO, World Health Organization; YLDs, years lived with disability.

Based on the uncertainty intervals of all input variables, we estimated that high BMI was responsible for between 18.4 and 61.0 million prevalent cases of LBP, 7.5 and 32.5 million prevalent cases of gout, 32.4 and 131.1 million prevalent cases of osteoarthritis, and 3.0 and 15.0 million YLDs (Table 1). High BMI was also associated with $30.7 and $100.5 billion of health care costs, $53.1 and $232.7 billion of productivity losses, and $83.8 and $333.1 billion of total costs (Table 2).

## Discussion

Our results suggest that high BMI was responsible for 7.3 million YLDs of musculoskeletal disorders, which cost the global economy approximately $180.7 billion in 2019. Of these, $60.5 billion were health care costs, which exceeded the GDP in 113 out of 192 countries and territories. In addition, the productivity losses due to morbidity were $120.2 billion.

In 2019, the total costs representing a high percentage of GDP was observed not only in high-income countries but also in low-income and lower-middle–income countries. Therefore, the increased prevalence of overweight and obesity should not be simply treated as a result of increased income.^31^ The comprehensive effects of rapid urbanization should also be taken into account. Although urbanization has played a key role in increasing BMI by changing eating habits and physical exercise, increasing BMI in rural areas is the leading contributor to the obesity epidemic globally.^32^ Particularly, BMI is rising at the same rate or even faster in rural dwellers (except for women in sub-Saharan Africa) than in their urban peers in low-income and middle-income countries.^32^ Therefore, the total costs accounting for a substantial proportion of GDP in low-income and lower-middle–income countries could be a function of increased BMI in rural settings.

Interestingly, although there were a higher rate and a larger number of rural residents in Africa than in other economically developed regions (eg, the Americas),^33^ Africa suffered least from the disease burden. The possible reason might be that Africa was less affected by high BMI. The prevalence rates of overweight (34.5%) and obesity (12.7%) in Africa were much lower than those (overweight, 64.2%; obesity, 28.3%) in the Americas.^34^ As high BMI increased the risk for musculoskeletal disorders, the number of prevalent cases and YLDs of musculoskeletal disorders were also lower in Africa than in the Americas.^12^ Consequently, the prevalence of high BMI among cases of musculoskeletal disorders would be lower, and lower PAFs were observed in Africa than in the Americas.^12^ Therefore, the high BMI–attributable disease burden was lower in Africa than in the Americas. Another explanation might be poor diagnostic preparedness. A shortage of health professionals and medical equipment in Africa hindered timely and precise diagnosis and a proportion of cases were underdiagnosed.^35^

In this study, we also found that the economic burden was not directly proportional to the population size and morbidity. The possible reasons might be population aging and increased life expectancy. We took the disease and economic burden in Southeast Asia and the Americas for example. In 2019, the number of people aged 65 years and older accounted for 8.6% and 16.0% of the total population in Latin America and the Caribbean and in North America, respectively.^36^ In contrast, this proportion was 7.2% in Southeast Asia.^36^ Additionally, life expectancy at 60 years was 22.7 years in the Americas, whereas it was 19.1 years in Southeast Asia.^37^ As the musculoskeletal disease burden increased with age,^3^ the disease and economic burden was heavier in the region with a higher proportion of older people. The prevalence of people classified as overweight or with obesity also contributed. Overweight prevalence was 24.3% in Southeast Asia compared with 64.2% in the Americas, and obesity prevalence was 6.2% in Southeast Asia compared to 28.3% in the Americas.^34^

Despite the noted burden, it is difficult to take action, such as increasing access to and delivering healthy food in low-income and lower-middle–income countries, where resources are often scarce and undernutrition brings additional threats.^38^ Most often, these countries seek external funding to prioritize interventions to address nutritional deficiencies. Therefore, food security outweighs increasing BMI in these areas.^31^ To enhance the double-duty actions, more adjustments are needed in administration and funding.^39^ New food policies such as enhancing healthy education and behaviors, taxes on unhealthy foods, and encouraging healthy food production were proposed in 2015.^40^ However, given the increasing burden of high BMI, it could be concluded that these policies might achieve success in the long term, but only slight progress in the short term.^31^ To tackle the significant burden effectively, more substantial efforts from health care professionals should be made to prevent excess weight gain, such as launching awareness-raising initiatives, delivering knowledge about weight management, and monitoring weight gain.^41^

Several studies have shown the mechanisms of high BMI in musculoskeletal disorders. Lee et al^42^ found that as weight increased, the lumbar spine and intervertebral discs were compressed, leading to LBP. In addition to the elevated loading borne by joints, excessive pressure worsened cartilage loss and caused osteoarthritis.^43,44^ Furthermore, high BMI contributed to gout by increasing the levels of serum uric acid,^45^ contributing to monosodium urate crystal deposits and systemic inflammation.^46^

In this study, the total costs of high BMI ranged from $83.8 to $333.1 billion, presenting approximately a 4-fold difference between the lower and upper limits. In contrast, the total costs of physical inactivity estimated in a previous global work ranged from $18.5 to $182.1 billion,^19^ nearly a 10-fold variation existing in the uncertainty levels. The disparities were derived from the differences in the uncertainty intervals of input variables. Conducting a sensitivity analysis with the lower and upper limits of input variables did not necessarily indicate the presence of extreme situations.^19^ Monte Carlo simulation could be an ideal strategy. However, lacking a full understanding of the distributions of input data impeded such efforts.^19^

### Limitations

This study had several limitations. First, we assumed that nonmarket production equally accounted for 23% of GDP in high-income and upper-middle–income countries, and 35% of GDP in lower-middle–income and low-income countries. Although this might not be in line with the actual situations, such data in most countries were absent. Second, our health outcomes only included prevalence estimates and YLDs without capturing psychosociological impact due to the absence of standardizing measurement tools. Third, the input variables of a country segmented into urban and rural areas would provide additional information on the distribution of burden across settings. However, only the aggregate data of a country are available. Fourth, because the prevalence of high BMI among cases of musculoskeletal disorders by category and the relative risk adjusted for sociodemographics were not estimated in the GBD result tool,^12^ we could not provide results with the adjusted PAFs. Fifth, smoking was also a risk factor for musculoskeletal disorders, and high BMI was also a risk factor for cardiovascular diseases.^6^ However, due to the limited article length, we could not investigate all relevant risk factors and diseases given different PAFs. Future efforts are warranted to address these issues.

Additionally, several missing values were imputed. For example, the spending shares by sector were not available in Albania in 2019, which were replaced with its latest values in 2018. The missing rate for this variable was 2.1% only, which was unlikely to make substantial changes to the regional and global estimates. Despite these, the socioeconomic factors in a country change over time, which might affect the interpretation of our results. This study also used data from other databases. Limiting analysis to those countries with complete data could reduce the sample size and valuable information provided by other variables would not be used. However, different databases might apply different methodologies during the data estimation process, and it is challenging to determine whether data are comparable.^47^ Therefore, future work aiming at providing more available estimates is needed to generate global estimates.

## Conclusions

Substantial health and economic outcomes of musculoskeletal disorders were associated with high BMI. More effective policies and active participation from health professionals are recommended to prevent excess weight gain. More available estimates are also needed to facilitate a global analysis.

## Notes

Supplement 1.
eMethods.
**eTable 1.** Sources or Calculation Methods for the Labor Force **eTable 2.** Locations With Missing Values, Substitutes, and Income Levels **eTable 3.** A Summary of Variables in This Study **eFigure 1.** Study Selection **eTable 4.** Study Ranking **eTable 5.** Health Care Costs per Case of Low Back Pain, Gout, and Osteoarthritis in the US **eFigure 2.** Comparisons of Health Care Costs per Case (US$) **eTable 6.** Disease Burden of Low Back Pain, Gout, and Osteoarthritis Attributable to High Body Mass Index by Location in 2019 (Amount, Uncertainty Interval) **eTable 7.** Disease Burden of Musculoskeletal Disorders Attributable to High Body Mass Index by Sex, Age, and World Bank Income Group, 2019 (Number in Thousands) **eTable 8.** Health Care, Morbidity-related, and Total Costs of Musculoskeletal Disorders Attributable to High Body Mass Index by Location in 2019 **eTable 9.** Health Care Costs of Low Back Pain Attributable to High Body Mass Index by Location in 2019 **eTable 10.** Health Care Costs of Gout Attributable to High Body Mass Index by Location in 2019 **eTable 11.** Health Care Costs of Osteoarthritis Attributable to High Body Mass Index by Location in 2019 **eTable 12.** Health Care Costs of Musculoskeletal Disorders Attributable to High Body Mass Index Borne by Sector and Location in 2019 **eTable 13.** Health Care, Morbidity-related, and Total Costs of Musculoskeletal Disorders Attributable to High Body Mass Index by World Bank Income Group in 2019
eReferences.

Supplement 2.
Data Sharing Statement
