# Supplementary material for: Health and Economic Outcomes Associated With Musculoskeletal Disorders Attributable to High Body Mass Index in 192 Countries and Territories in 2019
Source: JAMA Netw Open. 2023 Jan 20;6(1):e2250674. doi: 10.1001/jamanetworkopen.2022.50674 (PMC9860530; doi:10.1001/jamanetworkopen.2022.50674)
Supplement: Supplement 1. — eMethods. eTable 1. Sources or Calculation Methods for the Labor Force eTable 2. Locations With Missing Values, Substitutes, and Income Levels eTable 3. A Summary of Variables in This Study eFigure 1. Study Selection eTable 4. Study Ranking eTable 5. Health Care Costs per Case of Low Back Pain, Gout, and Osteoarthritis in the US eFigure 2. Comparisons of Health Care Costs per Case (US$) eTable 6. Disease Burden of Low Back Pain, Gout, and Osteoarthritis Attributable to High Body Mass Index by Location in 2019 (Amount, Uncertainty Interval) eTable 7. Disease Burden of Musculoskeletal Disorders Attributable to High Body Mass Index by Sex, Age, and World Bank Income Group, 2019 (Number in Thousands) eTable 8. Health Care, Morbidity-related, and Total Costs of Musculoskeletal Disorders Attributable to High Body Mass Index by Location in 2019 eTable 9. Health Care Costs of Low Back Pain Attributable to High Body Mass Index by Location in 2019 eTable 10. Health Care Costs of Gout Attributable to High Body Mass Index by Location in 2019 eTable 11. Health Care Costs of Osteoarthritis Attributable to High Body Mass Index by Location in 2019 eTable 12. Health Care Costs of Musculoskeletal Disorders Attributable to High Body Mass Index Borne by Sector and Location in 2019 eTable 13. Health Care, Morbidity-related, and Total Costs of Musculoskeletal Disorders Attributable to High Body Mass Index by World Bank Income Group in 2019 eReferences. [file jamanetwopen-e2250674-s001.pdf]

Supplemental Online Content

Chen N, Fong DYT, Wong JYH. Health and economic outcomes associated with musculoskeletal disorders attributable to high body mass index in 192 countries and territories in 2019. *JAMA Netw Open.* 2023;6(1):e2250674. doi:10.1001/jamanetworkopen.2022.50674

eMethods.

eTable 1. Sources or Calculation Methods for the Labor Force

eTable 2. Locations With Missing Values, Substitutes, and Income Levels

eTable 3. A Summary of Variables in This Study

eFigure 1. Study Selection

eTable 4. Study Ranking

eTable 5. Health Care Costs per Case of Low Back Pain, Gout, and Osteoarthritis in the US

eFigure 2. Comparisons of Health Care Costs per Case (US$)

eTable 6. Disease Burden of Low Back Pain, Gout, and Osteoarthritis Attributable to High Body Mass Index by Location in 2019 (Amount, Uncertainty Interval)

eTable 7. Disease Burden of Musculoskeletal Disorders Attributable to High Body Mass Index by Sex, Age, and World Bank Income Group, 2019 (Number in Thousands)

eTable 8. Health Care, Morbidity-related, and Total Costs of Musculoskeletal Disorders Attributable to High Body Mass Index by Location in 2019

eTable 9. Health Care Costs of Low Back Pain Attributable to High Body Mass Index by Location in 2019

eTable 10. Health Care Costs of Gout Attributable to High Body Mass Index by Location in 2019

© 2023 Chen N et al. *JAMA Network Open.*

eTable 11. Health Care Costs of Osteoarthritis Attributable to High Body Mass Index by Location in 2019

eTable 12. Health Care Costs of Musculoskeletal Disorders Attributable to High Body Mass Index Borne by Sector and Location in 2019

eTable 13. Health Care, Morbidity-related, and Total Costs of Musculoskeletal Disorders Attributable to High Body Mass Index by World Bank Income Group in 2019

eReferences.

This supplemental material has been provided by the authors to give readers additional information about their work.

eMethods

Data sources

We obtained data on the prevalent estimates, years lived with disability (YLDs), and population attributable fractions (PAFs) from the Global Burden of Diseases, Injuries, and Risk Factors Study (GBD) 2019 database.[1] These variables were computed with 95% uncertainty intervals (ie, the 25 th and 975 th values of the 1000 ordered draws in the posterior distribution).[2,3]

We also extracted the shares of health-care costs borne by the public, private, and out-of-pocket sectors from the World Health Organization (WHO) Global Health Expenditure Database.[4] Given that several countries did not have this estimate in 2019, we used its shares in the latest year, ie, Albania (2018), Libya (2011), Yemen (2015), and Syria (2012). Gross domestic product (GDP) data in 2019 were collected from the WHO Global Health Expenditure Database.[4] However, we also collected data from the World Bank,[5] if they were missing in the WHO Global Health Expenditure Database,[4] such as the GDP of Syria, Albania, and Libya in 2019. Additionally, Yemen's GDP data in 2019 was missing, which was replaced with its GDP in 2018 in the World Bank database.[5]

Further, we extracted the number of laborers in country i (L_i) from the World Bank database.[5] Due to the lack of this data in several countries and territories (ie, the Marshall Islands, San Marino, Kiribati, Cook Islands, and Seychelles), we obtained this estimate of these five countries and territories from the International Labour Organization (ILO) data repository.[6]

Given that the number of laborers in Grenada, Antigua and Barbuda, and Micronesia (Federated States of) was not available either in the World Bank or the ILO data repositories, we arrived at this estimate by using the number of people aged 15 and older[7] multiplied by the labor force participation rate in people aged 15 and older.[8] As the number of people aged 15 and older[8] was not estimated for Dominica, Saint Kitts and Nevis, Andorra, Monaco, Palau, Nauru, and Tuvalu, we replaced those with the national populations provided in the World Bank database.[5]Because Niue's population could only be found on its national website[9] and updated in 2017 (**eTable 1**), we used this estimate for Niue in this study. Additional information on data preparation is provided in **eTable 1**.

To estimate the lost output due to morbidity, we obtained the labor income share in GDP in country i (α_i)[10] and the ratio of the number of laborers to the population size of age j, ie, (L_{ij}/N_{ij})[8] from the ILO websites. The ILO only provided the labor income share in GDP in country i (α_i) between 2004 and 2017, which was relatively stable. For this reason, we computed the mean value of the α_i during this period as the α_i in 2019 for each country and territory. The L_{ij}/N_{ij} for each age group was missing in 83 countries

© 2023 Chen N et al. *JAMA Network Open.*

and territories (**eTable 2**). Instead, the aggregate results of L_{ij}/N_{ij} in workers aged 15 and older, 15 to 24, and 25 and older were available. As such, the missing L_{ij}/N_{ij} for each age group in these 83 countries and territories was replaced with those of their neighboring countries based on the income level and the aggregate L_{ij}/N_{ij}, particularly in those aged 15 and older, and 25 and older (**eTable 2**). A summary of included variables in this study is provided in **eTable 3**.

Additional information on the disease burden estimation

In the GBD project, sequelae refer to the disabling health outcomes of a disease or injury, and the disability weights measure the severity levels of disability, ranging from 0 (ie, full health) to 1 (ie, death).[3] For example, low back pain (LBP) has 8 sequelae, including mild, moderate, severe, and most severe LBP with or without leg pain, and the disability weights range from 0.02 to 0.38.[3] YLDs by sequela were estimated as the prevalence of a sequela multiplied by the disability weight for that sequela.[3] Details on the YLD estimation are presented in a previous study.[3]

In the GBD data repository,[1] high BMI-attributable YLDs of LBP and gout were only estimated in individuals aged 20 years and older, and high BMI-attributable YLDs of osteoarthritis were only estimated in individuals aged 30 years and older. For this reason, we extracted the available data on high BMI-attributable YLDs of musculoskeletal disorders (MDs) by category from the GBD data repository.[1] Subsequently, we multiplied YLD counts of LBP in individuals aged 15 to 19 years by the high BMI PAF for the age-standardized YLD rate of LBP to arrive at high BMI-attributable YLDs of LBP in individuals aged 15 to 19 years. Similarly, we used this method to calculate high BMI-attributable YLDs of gout in individuals aged 15 to 19 years, and high BMI-attributable YLDs of osteoarthritis in individuals aged 15 to 29 years.

Systematic review

We conducted a systematic review of the English-language literature reporting the national health-care costs of MDs published in the latest ten years (ie, between January 1, 2012 and February 10, 2022). We searched PubMed using the following terms: "((cost[Title] OR spending[Title] OR expenditure[Title] OR econom*[Title]) AND (musculoskeletal[Title/Abstract] OR back pain[Title/Abstract] OR gout[Title/Abstract] OR osteoarthritis[Title/Abstract] OR neck pain[Title/Abstract] OR rheumatoid arthritis[Title/Abstract]))". The study selection process is summarized in **eFigure 1**, as suggested by the PRISMA statement.[11]

Totally, there were 1332 studies. We included not only those studies reporting health-care costs of at least one category of MDs or making comparisons of health-care costs between cases and controls, but also those studies conducting cost-effectiveness analysis which might also estimate the original health-care costs. In addition, 12 studies were identified from selected reviews. Therefore, 1344 studies were

© 2023 Chen N et al. *JAMA Network Open.*

identified, and 1338 studies were screened after removing 6 duplicates. We first screened the studies based on titles and abstracts. Subsequently, we screened full texts if we were not sure whether the studies were relevant. Because the national economic analyses of MDs were limited, "national estimates" were defined broadly. Some studies estimated the economic costs of MDs but used a large-scale representative sample (sample size >2000) selected from a national database were also included to conduct comparisons and ranking. We did not include pure reviews without providing specific health-care costs, studies with samples that were not selected from a national database or not large enough, and studies conducting the cost-effectiveness analysis among several interventions without estimating disease-specific health-care costs. In addition, we also excluded studies focusing on complication costs, as we did not estimate the costs of complications in our study. Furthermore, studies estimating only one component of health-care costs or using a subpopulation (ie, only females or severe cases) were also excluded. We also did not include two records that conducted cost projections or provided simulation results only. Finally, 14 studies (**eTable 4**) were assessed and ranked based on several criteria listed below.

Selection criteria in descending order of importance

1. Statistical methods: Most studies conducted descriptive analysis. Because a patient might have multiple diseases, we prioritized the study results adjusted for comorbidities, and dealt with data nonrepresentativeness.
2. Precision of study results: Studies using self-reported data were more vulnerable to social desirability, recall, and response bias than observed data. In addition, misclassification of health conditions might occur in self-reported data.
3. Sample size: Studies with larger sample sizes were preferable. We prioritized those studies using a national sample, although the specific sample size might not be provided.
4. Data source: The samples should be selected from a national database or a national sample.
5. Inclusiveness of diseases: We aimed to arrive at the health-care costs per case for LBP, gout, and osteoarthritis. Priority was given to studies including these three categories of MDs.
6. Year of publication: Old data might provide outdated information. We prioritized those studies using the latest data. However, we did not give priority to this benchmark because the bias from statistical flaws outweighed old estimates.
7. Study duration: Studies used data covering a period, representing an average level. However, this criterion was only considered in case we could not arrive at a conclusion based on the six criteria listed above.

Taken together, in terms of the study's representativeness, we ranked the US study first.

Additional information on the estimation of health-care costs per case in the US

In the previous US study, health-care costs were only estimated from 1996 to 2016.[12]

Therefore, we projected the health-care costs of the US from 2016 to 2019 based on the annual growth rate between 1996 and 2016.[12] In the US study,[12] only health-care spending on gout, osteoarthritis, and an aggregate health-care spending on both LBP and neck pain was available (ie, health-care spending on LBP was not estimated separately). For this reason, we assumed that health-care spending per case on LBP and neck pain was equal. We then extracted the prevalent cases of LBP, neck pain, gout, and osteoarthritis in the US from the GBD 2019 database.[1] Subsequently, we calculated the health-care spending per case on MDs by category in the US (**eTable 5**).

Methodological validity

In this study, we estimated the health-care costs per case for each country using an extrapolation method, which was based on the assumption that variations in the overall health spending per head contributed entirely to variations in the cross-country disease-specific costs per case. Despite the potential limitation of reducing generalizability, the extrapolation approach has been well applied in the global economic analyses of non-communicable diseases.[13-15]

Currently, no databases estimate national disease-specific costs per case. For this reason, we compared our estimates with those reported in previous studies[16-21] included in the systematic review shown below. Two national studies[16,18] did not provide prevalence estimates and per case costs. Therefore, we estimated per case costs by using the health-care costs estimated in these two studies divided by the location-year prevalence estimates extracted from the GBD result tool.[1] Currency exchange rates are US$0.9879 per €1 and US$0.0068 per JPY1, as of November 5, 2022. Health-care costs per case estimated with the extrapolation method in our study were generally comparable to previous estimates,[16-21] as the points in **eFigure 2** were close to the line y = x, with only an exception. The per case costs for LBP calculated by Dieleman et al[16] was US$1424.4 in the US in 2013, lower than that in our study (ie, US$2222.4) in 2019. A possible explanation was that per case costs for LBP in the US might increase during 2013-2016,[12] which was not taken into account when conducting comparisons.

Estimation of lost output due to morbidity

In this study, we calculated productivity loss per worker in country i by using the labor income share in GDP in country i (α_i) multiplied by GDP (Y_i), divided by the number of laborers (L_i). Like a previous report,[22] the output per worker in country i was assumed to be equal. Taking the unemployment rate in each age group into consideration, we estimated the lost output per worker of age j (W_{ij2019}) by using the ($\alpha_i Y_i/L_i$) multiplied by the employment late of age j (ie, the ratio of the size of the labor force, L_{ij}, to the population size of age j, N_{ij},),

$W_{ij2019} = (\alpha_i Y_i/L_i)*(L_{ij}/N_{ij})$ (1).

In equation (1), we assumed the α_i and L_{ij}/N_{ij} were constant over time.

© 2023 Chen N et al. *JAMA Network Open.*

Equation (1) was modified to include non-market production. According to previous estimates, household production was equivalent to 23%[23] of the US's GDP and 35%[22] of Ghana's GDP. Due to a lack of country-specific data on the proportion that household production accounts for national GDP, we assumed that non-market output equivalently contributed to 23% of GDP in high-income and upper-middle-income countries, and 35% of GDP in lower-middle-income and low-income countries. The equation (2) below was used to estimate the lost economic output per head due to morbidity in 2019 (W'_{ij2019}).

$W'_{ij2019} = (\alpha_i Y_i/L_i)*(L_{ij}/N_{ij}) + \lambda_i(\alpha_i Y_i/L_i)*[1-(L_{ij}/N_{ij})]$ (2),
in which the λ_i refers to the percentage of non-market production equally accounting for GDP in country i. In this study, the L_{ij}/N_{ij} and λ_j were assumed to be 0 in children and older people, ie, $L_{ij}/N_{ij} = 0$, $\lambda_j = 0$ ($j < 15$ or $j > 84$). Given that only the aggregate L_{ij}/N_{ij} in people aged 65 and older was available, we calculated the L_{ij}/N_{ij} for people aged 65 to 69, 70 to 74, 75 to 79, 80 to 84, and 85 and older based on the assumption that the L_{ij}/N_{ij} decreases linearly between age 65 and 85, and reached 0 at age 85 and older.[22]

We then multiplied the lost output per person in 2019 (W'_{ij2019}) by YLD counts of MDs attributable to high BMI to arrive at the lost economic output due to morbidity for a person of age j in country i, M_{ij}, which is calculated as
$M_{ij} = W'_{ij2019} * YLD_{ij}$ (3).
Finally, we summed M_{ij} across all ages and diseases as the lost output due to morbidity. The estimations in this study are also summarized in **eTable 3.**

An example of estimations

To enhance the understanding of our estimation, we took the calculation of disease burden, health-care costs, and lost output due to morbidity of LBP in the UK for example shown below. The cited information indicates where the data were extracted.

1. Attributable cases and YLDs
1) Attributable cases = Cases of LBP *PAF[1]
2) Attributable YLDs (for people aged 15-19) = YLDs of LBP *PAF[1]
3) Attributable YLDs (for people aged 20-84): extracted from the GBD result tool[1]

2. Health-care costs
1) Cost per case in the US = US spending on LBP[12]/Cases of LBP in the US[1]
The spending ratio = Overall health spending per head in the UK[24]/ Overall health spending per head in the US[24]
2) Cost per case in the UK= Cost per case in the US* the spending ratio
3) Attributable health-care costs= Attributable cases* cost per case in the UK

© 2023 Chen N et al. *JAMA Network Open.*

3. Lost output due to morbidity

1) The expected market production per worker of age j, ie, $W_{j2019} = (\alpha Y/L) * (L_j/N_j)$,

α: the labor income share in GDP[10]

Y: GDP[4]

L: the number of laborers[5]

L_j/N_j: the labor force participation rate of age j, ie, the ratio of the number of laborers (L_j) to the population size of age j (N_j)[8]

2) The market and non-market production per person, ie,

$W'_{ij2019} =(\alpha Y/L) *(L_j/N_j) +\lambda(\alpha Y/L) *[1-(L_j/N_j)]$

λ: The ratio of non-market production equally accounting for the UK's GDP

As the UK is a high-income country, we assumed λ to be 23%.

3) Productivity losses due to morbidity = W'_{ij2019} * Attributable YLDs

The method to estimate the lost output due to morbidity has been applied in previous studies.[22,25]

eTable 1. Sources or calculation methods for the labor force

Countries or territories	Sources or calculation methods for the labor force
Marshall Islands, San Marino, Kiribati, Cook Islands, and Seychelles	The International Labour Organization data repository[6]
The Grenada, Antigua and Barbuda, and Micronesia (Federated States of)	The number of people aged 15 and older[7] multiplied by the labor force participation rate in people aged 15 and older[8]
Dominica, Saint Kitts and Nevis, Andorra, Monaco, Palau, Nauru, and Tuvalu	The national populations in these countries and territories provided in the World Bank database[5] to multiply by the labor force participation rate in people aged 15 and older[8]
Niue	The national population extracted from the statistic official website of Niue[9] was used to multiply by the labor force participation rate in people aged 15 and older.[8]
Others	The World Bank database[5]

© 2023 Chen N et al. *JAMA Network Open.*

eTable 2. Locations with missing values, substitutes, and income levels

Locations with missing values	Income level of locations with missing values	Location substitutes	Income level of location substitutes
Afghanistan	Low	Guinea	Low
Algeria	Lower-middle	Guinea	Low
Andorra	High	Spain	High
Angola	Lower-middle	Kenya	Lower-middle
Antigua and Barbuda	High	Mexico	Upper-middle
Bahamas	High	Dominican Republic	Upper-middle
Bahrain	High	Qatar	High
Bangladesh	Lower-middle	Myanmar	Lower-middle
Benin	Lower-middle	Kenya	Lower-middle
Bhutan	Lower-middle	Myanmar	Lower-middle
Burkina Faso	Low	Guinea	Low
Burundi	Low	Rwanda	Low
Cabo Verde	Lower-middle	Senegal	Lower-middle
Cameroon	Lower-middle	Kenya	Lower-middle
Central African Republic	Low	Kenya	Lower-middle
Chad	Low	Guinea	Low
China	Upper-middle	Indonesia	Upper-middle
Comoros	Lower-middle	Rwanda	Low
Congo	Lower-middle	Rwanda	Low
Cuba	Upper-middle	Dominican Republic	Upper-middle
Democratic Republic of the Congo	Low	Rwanda	Low
Djibouti	Lower-middle	Somalia	Low
Dominica	Upper-middle	Saint Lucia	Upper-middle
Equatorial Guinea	Upper-middle	South Africa	Upper-middle
Eritrea	Low	Somalia	Low
Eswatini	Lower-middle	South Africa	Upper-middle
Ethiopia	Low	Somalia	Low
Fiji	Upper-middle	Cook Islands	High
Gabon	Upper-middle	South Africa	Upper-middle
Gambia	Low	Guinea	Low
Ghana	Lower-middle	Guinea	Low
Grenada	Upper-middle	Saint Lucia	Upper-middle
Guinea-Bissau	Low	Guinea	Low
Haiti	Low	Guinea	Low
Iraq	Upper-middle	Jordan	Upper-middle

© 2023 Chen N et al. *JAMA Network Open.*

Ivory Coast	Lower-middle	Guinea	Low
Kazakhstan	Upper-middle	Kyrgyzstan	Lower-middle
Kuwait	High	Qatar	High
Lao People's Democratic Republic	Lower-middle	Thailand	Upper-middle
Liberia	Low	Guinea	Low
Libya	Upper-middle	Guinea	Low
Madagascar	Low	Zimbabwe	Lower-middle
Malawi	Low	Zimbabwe	Lower-middle
Mali	Low	Guinea	Low
Mauritania	Lower-middle	Guinea	Low
Micronesia (Federated States of)	Lower-middle	Philippines	Lower-middle
Monaco	High	France	High
Morocco	Lower-middle	Guinea	Low
Mozambique	Low	Zimbabwe	Lower-middle
Namibia	Upper-middle	Botswana	Upper-middle
Nauru	High	Marshall Islands	Upper-middle
Nepal	Lower-middle	Myanmar	Lower-middle
Nicaragua	Lower-middle	Honduras	Lower-middle
Niger	Low	Guinea	Low
Niue	High	Cook Islands	High
Oman	High	Qatar	High
Palau	High	Japan	High
Papua New Guinea	Lower-middle	Indonesia	Upper-middle
Saint Kitts and Nevis	High	Mexico	Upper-middle
Saint Vincent and the Grenadines	Upper-middle	Saint Lucia	Upper-middle
San Marino	High	Italy	High
Sao Tome and Principe	Lower-middle	South Africa	Upper-middle
Saudi Arabia	High	Qatar	High
Sierra Leone	Low	Guinea	Low
Solomon Islands	Lower-middle	Indonesia	Upper-middle
South Sudan	Low	Kenya	Lower-middle
Sudan	Low	Somalia	Low
Suriname	Upper-middle	Guyana	Upper-middle
Syrian Arab Republic	Low	Guinea	Low
Tajikistan	Low	Kyrgyzstan	Lower-middle
Timor-Leste	Lower-middle	Indonesia	Upper-middle
Togo	Low	Guinea	Low
Tonga	Upper-middle	Cook Islands	High

© 2023 Chen N et al. *JAMA Network Open.*

Trinidad and Tobago	High	Colombia	Upper-middle
Tunisia	Lower-middle	Guinea	Low
Turkmenistan	Upper-middle	Kyrgyzstan	Lower-middle
Tuvalu	Upper-middle	Guinea	Low
Uganda	Low	Kenya	Lower-middle
Ukraine	Lower-middle	Belarus	Upper-middle
United Republic of Tanzania	Lower-middle	Rwanda	Low
Uzbekistan	Lower-middle	Kyrgyzstan	Lower-middle
Venezuela (Bolivarian Republic of)	Upper-middle	Colombia	Upper-middle

© 2023 Chen N et al. *JAMA Network Open.*

eTable 3. A summary of included variables in this study

<table>
<tr><th></th><th>Generated variables</th><th>Input variables</th><th>Input variables accompanied by 95% uncertainty intervals</th><th>Input data sources</th><th>Calculation methods or extraction</th></tr>
<tr><td rowspan="3">Disease burden</td><td>a. Attributable cases</td><td>a1. Cases of MDs by category
a2. PAFs</td><td>a1 and a2</td><td>GBD result tool[1]</td><td>a = a1*a2</td></tr>
<tr><td>b. Attributable YLDs</td><td>b1. YLDs of LBP and gout (for people aged 15-19), and osteoarthritis (for people aged 15-29)
b2. PAFs</td><td>b1 and b2</td><td>GBD result tool[1]</td><td>b = b1*b2</td></tr>
<tr><td>-</td><td>b3. YLDs of LBP and gout (for people aged 20-84) and osteoarthritis (for people aged 30-84)</td><td>b3</td><td>GBD result tool[1]</td><td>Extracted from the GBD result tool[1]</td></tr>
<tr><td rowspan="3">Health-care costs</td><td rowspan="2">c. Costs per case by category in the US</td><td>c1. Costs of MDs by category in the US</td><td>-</td><td>The US study[12]</td><td rowspan="2">c = c1/c2</td></tr>
<tr><td>c2. Cases of MDs by category in the US</td><td>-</td><td>GBD result tool[1]</td></tr>
<tr><td>d. Costs per case of MDs by category in each country</td><td>Overall health spending per capita in each country</td><td>-</td><td>The global health spending study[24]</td><td>The spending ratio = Overall health spending per capita in a country/Overall health spending per capita in the US</td></tr>
</table>

© 2023 Chen N et al. *JAMA Network Open.*

					d = c*the spending ratio
	e. Attributable health-care costs by category in each country	d and a	Listed above	Listed above	e = d*a
	f. Attributable health-care costs by sector	Expenditure shares	-	The WHO Global Health Expenditure Database[4]	f = The sum of attributable health-care costs (ie, e) across diseases*expenditure shares
Productivity losses	g. The lost market output per worker	g1. The labor income share in GDP in country *i* (α_i)	-	ILO database[10]	Extracted from the ILO database[10]
		g2. GDP (Y_i)	-	The WHO Global Health Expenditure Database,[4] and the World Bank[5]	Extracted from the WHO Global Health Expenditure Database,[4] and the World Bank[5]
		g3. The number of laborers (L_i)	-	The World Bank database,[5] and the ILO data repository[6]	The L_i in several countries and territories was calculated based on the labor force participation rate in

© 2023 Chen N et al. *JAMA Network Open.*

					people aged 15 and older (**eTable 1**).[8]
		g4. The ratio of the number of laborers to the population size of age j (L_{ij}/N_{ij})	-	ILO websites[8]	We estimated the lost output per worker of age j (W_{ij2019}) as follows- $W_{ij2019} = (\alpha_i Y_i/L_i)*(L_{ij}/N_{ij})$.
	h. The lost non-market output per person	The ratio of non-market production equally accounting for GDP in country i (λ_i)	-	The official website of the US government[23] and a previous study[22]	λ_i was assumed to be 23% in high-income and upper-middle-income countries, and 35% in lower-middle-income and low-income countries.<br><br>The lost market and non-market output per person, ie, $W'_{ij2019} = (\alpha_i Y_i/L_i)*(L_{ij}/N_{ij}) + \lambda_i(\alpha_i Y_i/L_i)*[1-(L_{ij}/N_{ij})]$.

Abbreviations: GBD, Global Burden of Diseases, Injuries, and Risk Factors Study; GDP, gross domestic product; ILO, International Labour Organization; LBP, low back pain; MDs, musculoskeletal disorders; PAFs, population attributable fractions; WHO, World Health Organization; YLDs, years lived with disability.

© 2023 Chen N et al. *JAMA Network Open.*

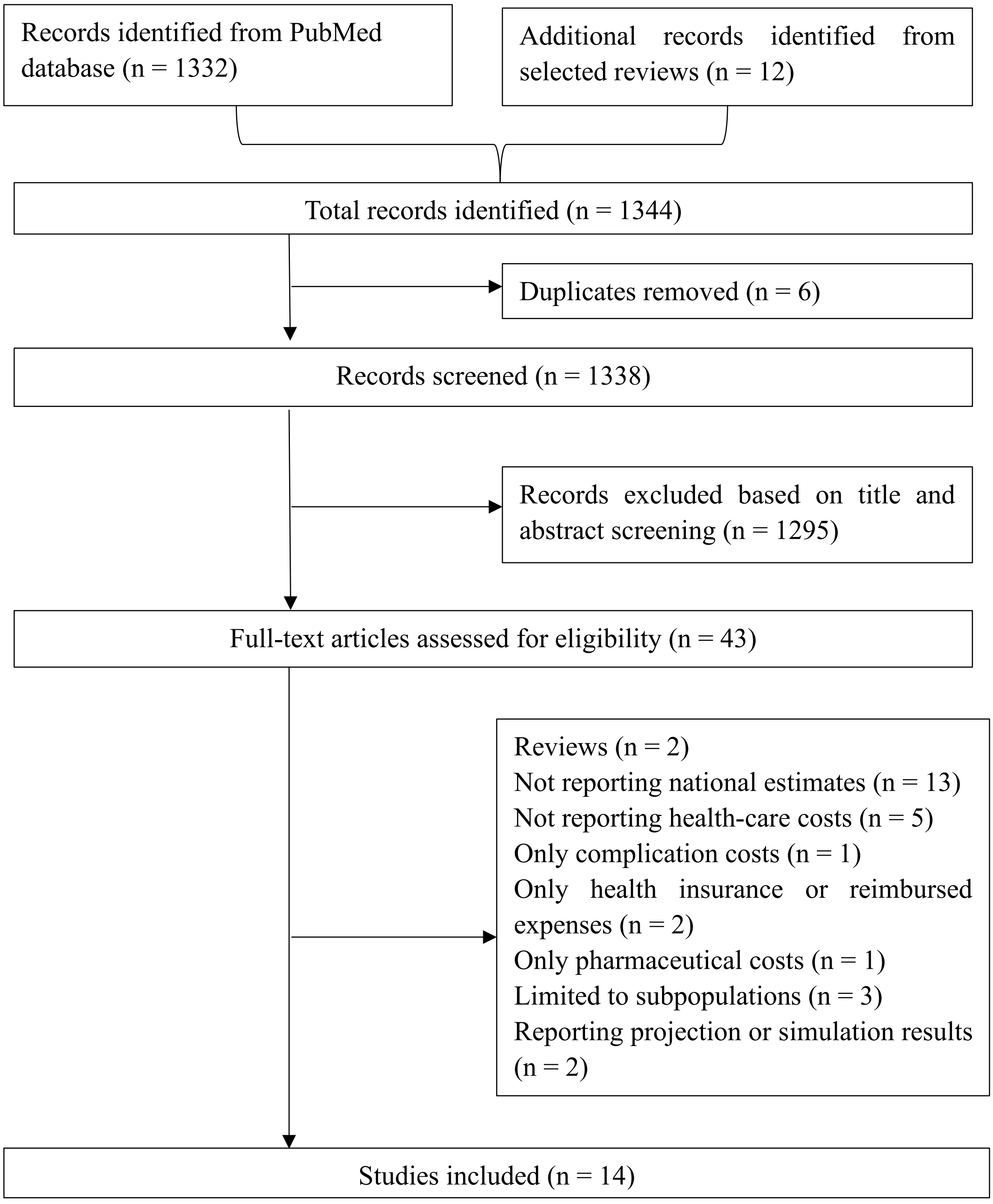

eFigure 1. Study selection

© 2023 Chen N et al. *JAMA Network Open.*

**eTable 4.** Study ranking									
Rank	Reference	Year of publi-cation	Coun-try	Data sources	Sample size	Statistical methods	Precision of study results	Inclusiveness of diseases	Study duration
1	Dieleman et al[12]	2020	The US	Government budgets, insurance claims, facility records, household surveys, and official US records.	A national sample. The sample size was not provided.	Spending estimates were adjusted for comorbidities. Data nonrepresentativeness was addressed and specific adjustments were discussed.	Samples were identified based on the diagnosis code.<br><br>The degree to which the parameters were constrained was determined.<br><br>The confidence intervals were calculated.	Low back, neck pain, osteoarthritis, gout, rheumatoid arthritis, and other MDs.	1996-2016
2	Dieleman et al[16]	2016	The US	Government budgets, insurance claims, facility surveys, household	A national sample. The sample size was not provided.	Spending was adjusted to reflect the health condition treated.	In some cases, a small number of cases were used as a basis for estimation.	Low back, neck pain, osteoarthritis, gout, rheumatoid	1996-2013

© 2023 Chen N et al. *JAMA Network Open.*

				surveys, and official US records.			The uncertainty intervals were calculated.	arthritis, and other MDs.	
3	Ebata-Kogure et al[17]	2020	Japan	The sample was selected from a medical claims database.	1,006,385	A descriptive analysis.	Not very precise because the costs were included regardless of whether they were incurred for hip or knee osteoarthritis or other concurrent diseases.<br><br>Uncertainty levels were not estimated, but the interquartile ranges of health-care costs were estimated.	Hip and knee osteoarthritis.	2013-2019
4	Lee et al[18]	2019	Korea	National Health Insurance claims data.	Nationally representative sample. The sample	The economic burden was estimated with a human resources	Not very precise because patients were defined based on their use	LBP and osteoarthritis.	2015

© 2023 Chen N et al. *JAMA Network Open.*

					size was not provided.	approach.	of health-care services, but there is a possibility that the number of patients was under- or overestimated.		
5	Ahn et al[26]	2016	Korea	The sample was selected from the Korean National Health Insurance claims database.	The sample size for patients with LBP was 111544.	A descriptive analysis.	Definitions of LBP and lumbar disorders relying solely on claims of disease diagnosis have limited accuracy.	LBP, intervertebral disc disorder, and spinal stenosis.	2011
6	Olafsson et al[19]	2018	Swe-den	The sample was selected from national registers and an administrative patient database.	129,973	Using a prevalence-based bottom-up approach.	Descriptive statistics accompanied by 95% CI<br><br>Limit to a region of Sweden<br><br>Some costs were not included (eg, out-of-pocket costs for over-the-	LBP	2008-2011

© 2023 Chen N et al. *JAMA Network Open.*

							counter drugs).		
7	Alonso-García and Sarría-Santamer[20]	2020	Spain	A nationally representative sample.	23,089	A descriptive analysis.	Not very precise because the information on LBP was self-reported.<br><br>Costs with missing values were imputed.	LBP	2017
8	Zhao et al[21]	2019	The US	Weighted nationally representative data from the 2015 Medical Expenditure Panel Survey	25,562,623	Rao-Scott chi-square tests for categorical variables and t-tests for continuous variables.	Using self-reported data	Osteoarthritis	2015
9	Flores et al[27]	2019	The US	National Health and Wellness Survey	2559	A descriptive analysis based on self-reported gout diagnosis and symptoms.	Using self-reported data	Gout	2012-2013
10	Williams et al[28]	2017	The US	The Medical Expenditures Panel Survey	35,313	A descriptive analysis.	Spending adjusted for functional limitations.	Arthritis and joint pain	2011

© 2023 Chen N et al. *JAMA Network Open.*

							Based on self-report data.		
11	Eriksson et al[29]	2015	Swe-den	Collected from national registries.<br><br>Costs data were derived from several sources.	49,829	A descriptive analysis.	The costs were underestimated due to the unavailable information on some cost components.<br><br>The uncertainty levels were calculated.	Rheumatoid arthritis	2010
12	Kalkan et al[30]	2014	Swe-den	Collected from national and regional registries.	Ranged from 2539 to 8058 annually.	A descriptive analysis.	Missing data were estimated based on assumptions.<br><br>Data for outpatient care and sick leave were not complete for all years in the 1990s, and average values were used, leading to possible over- or underestimation	Rheumatoid arthritis	1990-2010

© 2023 Chen N et al. *JAMA Network Open.*

							of minor importance for the total costs.		
13	Huscher et al[31]	2015	German	Collected from the National Database of the German Collaborative Arthritis Centers.	About 3400 patients annually.	Costs were calculated using fixed prices as well as annually updated cost factors.	The authors probably underestimated 12-months related components (ie, medication costs) due to memory bias.	Rheumatoid arthritis	2002-2011
14	Kinge et al[32]	2017	Norway	The Norwegian Directorate of Health.	The sample size was not provided.	A descriptive analysis.	This study did not provide the estimates we need because only an aggregate cost for the musculoskeletal system was available.	Musculoskeletal system	2013

Abbreviations: CI, confidence interval; LBP, low back pain; MDs, musculoskeletal disorders.

© 2023 Chen N et al. *JAMA Network Open.*

eTable 5. Health-care costs per case of low back pain, gout, and osteoarthritis in the US

Health condition	Health-care costs, 2016 US$ billion[a]	Annualized rate of change, 1996–2016 (%)[a]	Health-care costs, 2019 US$ billion	Prevalence, 2019[b]	Health-care costs per case, 2019 US$
Low back pain	95.6	6.6	115.8	52,105,428	2222.4
Gout	2.8	5.8	3.3	8,655,567	381.3
Osteoarthritis	80.0	7.7	99.9	51,865,889	1926.1

[a] Based on estimates from Dieleman et al[12]

[b] Based on estimates from the GBD 2019 Diseases and Injuries Collabourators[3]

© 2023 Chen N et al. *JAMA Network Open.*

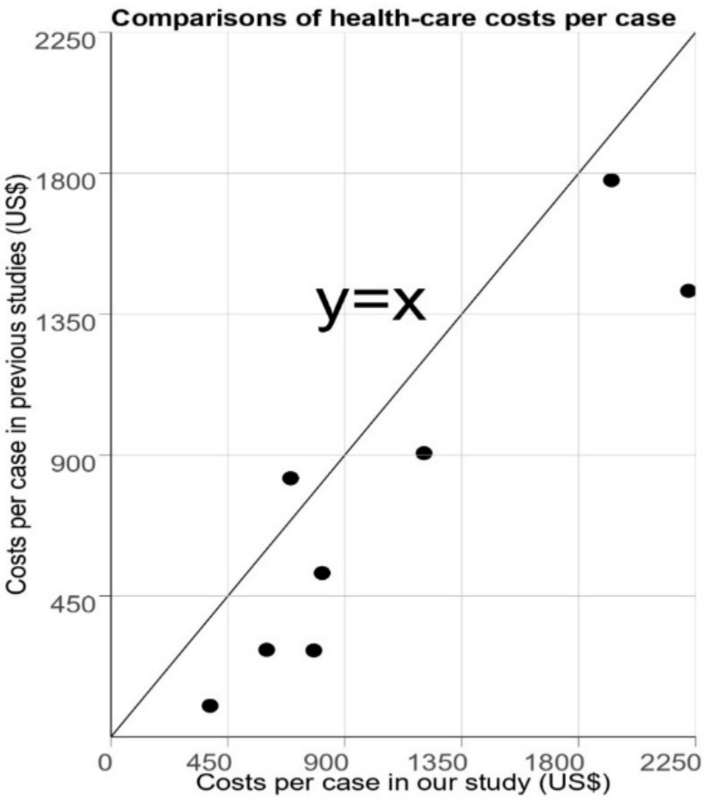

eFigure 2. Comparisons of health-care costs per case (US$)

© 2023 Chen N et al. *JAMA Network Open.*

eTable 6. Disease burden of low back pain, gout, and osteoarthritis attributable to high body mass index by location in 2019 (amount, uncertainty level)

	Low back pain		Gout		Osteoarthritis	
Location	Prevalence	YLDs	Prevalence	YLDs	Prevalence	YLDs
Global	36267110 (18437198 to 61011482)	4281563 (1857645 to 8146944)	16947252 (7454900 to 32457792)	508008 (214287 to 984921)	72969260 (32371084 to 131052677)	2556020 (912145 to 5824027)
Africa	2759627 (1375035 to 4752062)	305764 (125454 to 604680)	758571 (329691 to 1485300)	24079 (9475 to 49197)	3964819 (1814204 to 7032158)	142132 (51434 to 323229)
Algeria	285676 (151890 to 457322)	33514 (15260 to 61362)	82950 (40110 to 150719)	2598 (1106 to 5030)	443930 (218134 to 717656)	15483 (6152 to 34270)
Angola	48771 (21260 to 90401)	5302 (1806 to 11319)	12555 (4528 to 26958)	412 (131 to 925)	63278 (24502 to 122093)	2360 (713 to 5652)
Benin	36435 (18325 to 62022)	3910 (1633 to 7687)	8758 (3934 to 16633)	288 (113 to 593)	48948 (22767 to 86221)	1810 (665 to 4132)
Botswana	9249 (5394 to 14363)	1017 (471 to 1868)	3367 (1600 to 6249)	104 (41 to 210)	17822 (9137 to 29276)	622 (245 to 1377)
Burkina Faso	44535 (19388 to 84036)	4762 (1671 to 10264)	9313 (3365 to 20329)	298 (93 to 671)	63811 (25138 to 123214)	2302 (722 to 5468)
Burundi	11058 (3935 to 23095)	1207 (339 to 2832)	2925 (836 to 7306)	95 (23 to 239)	14323 (4883 to 30864)	519 (130 to 1317)
Cabo Verde	2389 (1279 to 3953)	276 (122 to 522)	770 (360 to 1446)	24 (9 to 48)	4521 (2198 to 7743)	158 (60 to 354)
Cameroon	113130 (59622 to 187541)	12207 (5330 to 23418)	27598 (12841 to 51004)	875 (353 to 1763)	148843 (73404 to 248944)	5315 (2062 to 11905)
Central African Republic	5456 (1949 to 11553)	596 (166 to 1396)	1308 (375 to 3272)	43 (10 to 107)	6991 (2199 to 15258)	258 (65 to 649)
Chad	19903 (8112 to 38096)	2066 (669 to 4546)	4283 (1396 to 9971)	137 (39 to 323)	24180 (8797 to 48457)	872 (250 to 2105)
Comoros	1914 (915 to 3382)	222 (86 to 448)	576 (226 to 1197)	18 (6 to 40)	2944 (1207 to 5590)	105 (34 to 246)

© 2023 Chen N et al. *JAMA Network Open.*

Congo	16659 (9035 to 27335)	1891 (813 to 3635)	5055 (2258 to 9646)	167 (64 to 344)	24918 (11709 to 43018)	910 (339 to 2036)
Democratic Republic of the Congo	119975 (49724 to 230839)	12685 (4130 to 28030)	36104 (12918 to 78970)	1124 (342 to 2608)	162531 (63546 to 326723)	5858 (1746 to 14078)
Equatorial Guinea	5208 (2884 to 8445)	568 (251 to 1077)	1245 (603 to 2203)	40 (16 to 79)	5765 (2916 to 9782)	204 (79 to 453)
Eritrea	6813 (2901 to 12837)	671 (215 to 1492)	2184 (782 to 4828)	63 (18 to 149)	11955 (4411 to 23361)	398 (108 to 978)
Eswatini	4682 (2551 to 7349)	492 (223 to 913)	1685 (847 to 2951)	50 (22 to 97)	8708 (4480 to 13944)	300 (122 to 657)
Ethiopia	133552 (54173 to 259601)	15039 (5001 to 32889)	38867 (12745 to 88280)	1274 (413 to 2853)	162527 (59512 to 333345)	5930 (1707 to 14436)
Gabon	8095 (4269 to 13062)	924 (415 to 1736)	2441 (1130 to 4500)	77 (32 to 151)	12772 (6252 to 21215)	460 (179 to 1028)
Gambia	5660 (2791 to 9833)	607 (245 to 1221)	1519 (644 to 2972)	49 (18 to 103)	8098 (3641 to 14465)	290 (104 to 657)
Ghana	102261 (56419 to 164425)	11364 (5087 to 21456)	31554 (14956 to 58008)	996 (399 to 1994)	190601 (94902 to 322196)	6813 (2613 to 15346)
Guinea	23309 (10838 to 42330)	2500 (916 to 5245)	5548 (2042 to 12047)	176 (56 to 395)	33685 (13743 to 63940)	1197 (383 to 2800)
Guinea-Bissau	3196 (1380 to 6084)	340 (116 to 735)	714 (258 to 1563)	23 (7 to 52)	4285 (1667 to 8306)	157 (48 to 373)
Ivory Coast	71045 (35071 to 123014)	8031 (3279 to 15741)	18291 (7422 to 36733)	608 (225 to 1284)	92763 (41276 to 166678)	3476 (1223 to 7881)
Kenya	131085 (67420 to 223807)	14829 (6289 to 28764)	39482 (17606 to 76540)	1295 (558 to 2547)	170821 (78224 to 304356)	6305 (2324 to 14141)
Lesotho	7317 (3920 to 11862)	794 (351 to 1494)	2279 (1078 to 4261)	69 (28 to 140)	13601 (6754 to 23181)	475 (182 to 1054)
Liberia	15975 (8388 to 26430)	1789 (790 to 3424)	4244 (1923 to 7988)	143 (55 to 290)	22275 (10649 to 38134)	826 (316 to 1846)
Madagascar	35993 (15906 to 65940)	3921 (1333 to 8384)	10320 (3758 to 22605)	337 (105 to 793)	52061 (20113 to 102112)	1877 (546 to 4520)

© 2023 Chen N et al. *JAMA Network Open.*

Malawi	32689 (14077 to 60688)	3548 (1229 to 7490)	8629 (3209 to 18102)	269 (86 to 593)	43439 (17058 to 84309)	1535 (468 to 3620)
Mali	34026 (15244 to 63136)	3590 (1284 to 7617)	9239 (3448 to 19605)	297 (97 to 658)	53348 (21510 to 100459)	1921 (618 to 4464)
Mauritania	15075 (8171 to 24772)	1656 (734 to 3156)	4491 (2116 to 8326)	139 (57 to 278)	24468 (12318 to 40905)	863 (335 to 1929)
Mauritius	7722 (4018 to 12908)	915 (396 to 1740)	4066 (1736 to 7773)	124 (50 to 251)	15527 (6929 to 26890)	519 (185 to 1159)
Mozambique	46292 (20638 to 84062)	5222 (1852 to 10742)	10793 (3981 to 23335)	354 (110 to 800)	55184 (21752 to 107812)	2017 (612 to 4813)
Namibia	7728 (4417 to 12356)	836 (374 to 1573)	2666 (1280 to 4897)	81 (32 to 163)	13988 (7100 to 23863)	483 (184 to 1073)
Niger	28472 (11978 to 54802)	2959 (998 to 6518)	6152 (2110 to 14239)	202 (59 to 473)	37893 (14328 to 75804)	1392 (416 to 3321)
Nigeria	630730 (314374 to 1087273)	69471 (28604 to 138456)	127274 (55380 to 249798)	4126 (1735 to 8112)	715885 (314830 to 1308373)	26209 (9468 to 58932)
Rwanda	23517 (9601 to 45011)	2577 (852 to 5609)	5504 (1879 to 12372)	173 (51 to 399)	33406 (12445 to 66513)	1197 (351 to 2921)
Sao Tome and Principe	657 (336 to 1099)	74 (32 to 143)	211 (95 to 402)	7 (3 to 14)	1143 (550 to 1981)	41 (16 to 93)
Senegal	35206 (18004 to 60893)	3865 (1555 to 7706)	10065 (4193 to 20271)	316 (114 to 671)	59527 (26370 to 105690)	2120 (732 to 4870)
Seychelles	598 (308 to 1009)	74 (33 to 140)	284 (124 to 543)	9 (4 to 18)	1034 (470 to 1789)	37 (13 to 83)
Sierra Leone	14583 (6174 to 27854)	1563 (522 to 3411)	3535 (1189 to 7940)	113 (33 to 262)	21045 (8069 to 41078)	761 (230 to 1822)
South Africa	310618 (179043 to 479891)	34812 (16967 to 62105)	128802 (64882 to 224391)	3909 (1857 to 7172)	644839 (347993 to 1016254)	22434 (9425 to 48918)

© 2023 Chen N et al. *JAMA Network Open.*

South Sudan	20595 (9933 to 36251)	2271 (901 to 4552)	5464 (2230 to 11037)	174 (63 to 369)	27600 (12146 to 50506)	978 (345 to 2223)
Togo	18228 (9036 to 32045)	2023 (800 to 4130)	4385 (1764 to 8992)	142 (48 to 305)	28377 (12217 to 52658)	1036 (352 to 2440)
Uganda	68074 (30470 to 123762)	7299 (2633 to 15127)	16354 (6163 to 34339)	523 (173 to 1148)	84728 (34822 to 162317)	3052 (976 to 7109)
United Republic of Tanzania	121912 (62482 to 208169)	13465 (5487 to 26494)	36105 (15629 to 72083)	1141 (409 to 2405)	184086 (82431 to 328140)	6593 (2312 to 15243)
Zambia	35991 (17353 to 63778)	3942 (1508 to 8090)	9949 (4086 to 19882)	323 (113 to 697)	47954 (20900 to 88044)	1728 (589 to 3953)
Zimbabwe	37572 (19639 to 63344)	4079 (1685 to 7979)	8667 (3657 to 17790)	274 (98 to 580)	54391 (23806 to 98702)	1937 (689 to 4484)
The Americas	10187105 (5732953 to 15709360)	1181586 (568778 to 2093267)	5628341 (2897828 to 9321606)	161240 (80145 to 279076)	17395805 (8698214 to 28824471)	613766 (246600 to 1343962)
Antigua and Barbuda	503 (260 to 830)	61 (27 to 116)	96 (44 to 175)	3 (1 to 6)	1273 (607 to 2168)	45 (17 to 101)
Argentina	262329 (126688 to 451237)	30616 (12963 to 59082)	177989 (77740 to 335389)	5303 (2191 to 10437)	625917 (291074 to 1093626)	22352 (8369 to 50367)
Bahamas	2474 (1330 to 3950)	298 (137 to 549)	449 (224 to 804)	14 (6 to 29)	5926 (2919 to 9802)	210 (81 to 465)
Barbados	2376 (1242 to 3857)	284 (129 to 526)	471 (231 to 845)	14 (6 to 28)	7000 (3388 to 11560)	238 (92 to 527)
Belize	2404 (1303 to 3762)	279 (129 to 511)	382 (189 to 675)	12 (5 to 24)	4419 (2173 to 7147)	156 (61 to 345)
Bolivia (Plurinational State of)	57317 (29490 to 94187)	6607 (2911 to 12466)	10619 (4981 to 19298)	337 (134 to 674)	121092 (59593 to 198515)	4313 (1669 to 9611)
Brazil	1520278 (816496 to 2456595)	182280 (83734 to 337309)	261936 (130106 to 462127)	7996 (3772 to 14637)	3205837 (1642826 to 5289373)	109456 (43766 to 243250)
Canada	391284 (207213 to 632944)	46728 (21542	399558 (184057 to 733296)	11658 (5218	550545 (254215 to 969187)	19062 (7573 to 41840)

© 2023 Chen N et al. *JAMA Network Open.*

		to 85347)		to 21452)		
Chile	142440 (71391 to 237419)	16822 (7482 to 31360)	94656 (42533 to 174790)	2826 (1209 to 5363)	342119 (164185 to 582151)	12281 (4748 to 27502)
Colombia	311577 (156798 to 520706)	36964 (15925 to 70549)	33368 (15753 to 60810)	1038 (395 to 2143)	637591 (300616 to 1079644)	21542 (8087 to 48647)
Costa Rica	30115 (15053 to 49822)	3573 (1562 to 6755)	3763 (1795 to 6808)	117 (45 to 238)	67756 (31557 to 115232)	2307 (872 to 5199)
Cuba	91468 (48985 to 145803)	10889 (5094 to 19872)	16981 (8288 to 30387)	502 (201 to 992)	246645 (119192 to 411772)	8265 (3192 to 18453)
Dominica	498 (266 to 793)	58 (27 to 108)	92 (45 to 165)	3 (1 to 6)	1277 (633 to 2077)	43 (17 to 94)
Dominican Republic	49943 (24641 to 84538)	5906 (2531 to 11363)	9166 (4230 to 16946)	292 (108 to 599)	109592 (49325 to 188335)	3857 (1416 to 8771)
Ecuador	100824 (58008 to 151615)	11777 (5677 to 20710)	24883 (12775 to 42883)	781 (333 to 1477)	253249 (133982 to 389205)	9078 (3665 to 20005)
El Salvador	37121 (18393 to 61714)	4262 (1841 to 8085)	3922 (1871 to 7076)	120 (44 to 245)	75919 (34335 to 129583)	2525 (953 to 5690)
Grenada	606 (321 to 994)	73 (33 to 138)	103 (49 to 190)	3 (1 to 7)	1411 (685 to 2378)	50 (19 to 111)
Guatemala	65306 (30099 to 117189)	7292 (2736 to 14865)	6081 (2516 to 12016)	192 (65 to 408)	103589 (44937 to 186608)	3552 (1224 to 8217)
Guyana	3748 (1897 to 6286)	438 (188 to 836)	587 (271 to 1087)	19 (7 to 38)	7705 (3561 to 13248)	272 (101 to 612)
Haiti	21035 (8248 to 41345)	2387 (750 to 5244)	3033 (1073 to 6760)	98 (28 to 231)	34940 (12578 to 71493)	1249 (342 to 3047)
Honduras	38506 (17742 to 67247)	4314 (1669 to 8665)	3722 (1597 to 7201)	118 (40 to 250)	64369 (28054 to 114204)	2236 (788 to 5124)
Jamaica	18673 (10173 to 29589)	2177 (1011 to 3987)	3185 (1611 to 5637)	96 (38 to 192)	41629 (21121 to 67883)	1398 (559 to 3076)

© 2023 Chen N et al. *JAMA Network Open.*

Mexico	806363 (416624 to 1313358)	94838 (42833 to 176450)	107705 (52610 to 191065)	3381 (1571 to 6287)	1681697 (818145 to 2809029)	59546 (23486 to 131464)
Nicaragua	31043 (14981 to 52750)	3574 (1485 to 6991)	3195 (1455 to 5945)	103 (38 to 214)	54211 (24729 to 93545)	1891 (704 to 4272)
Panama	21388 (10729 to 35559)	2505 (1064 to 4744)	2717 (1273 to 5054)	85 (32 to 175)	48615 (22822 to 82531)	1647 (616 to 3742)
Paraguay	37334 (19362 to 62046)	4379 (1928 to 8385)	5656 (2653 to 10424)	177 (69 to 361)	67365 (32489 to 114007)	2316 (882 to 5204)
Peru	176617 (85854 to 293398)	20613 (8820 to 39411)	37532 (17207 to 68692)	1151 (450 to 2338)	420532 (195319 to 707296)	14310 (5360 to 32051)
Saint Kitts and Nevis	393 (207 to 642)	48 (22 to 89)	76 (37 to 135)	2 (1 to 5)	985 (483 to 1637)	35 (14 to 79)
Saint Lucia	1201 (626 to 1952)	144 (65 to 267)	211 (101 to 381)	7 (3 to 13)	2903 (1386 to 4811)	100 (38 to 223)
Saint Vincent and the Grenadines	677 (343 to 1113)	81 (36 to 153)	128 (59 to 238)	4 (2 to 8)	1708 (802 to 2869)	59 (23 to 133)
Suriname	3316 (1682 to 5443)	393 (172 to 738)	600 (285 to 1104)	19 (7 to 39)	7981 (3698 to 13342)	278 (105 to 620)
Trinidad and Tobago	10855 (5963 to 17025)	1303 (610 to 2373)	2079 (1046 to 3665)	64 (26 to 125)	28755 (14625 to 46427)	990 (398 to 2171)
United States of America	5769970 (3443558 to 8463897)	658535 (334677 to 1114745)	4376671 (2312452 to 7040919)	123589 (63663 to 207798)	8142942 (4183684 to 13276933)	293421 (121874 to 629821)
Uruguay	23172 (11469 to 39315)	2700 (1148 to 5153)	16871 (7401 to 31863)	485 (201 to 943)	62023 (28970 to 107391)	2160 (813 to 4817)
Venezuela (Bolivarian Republic of)	153952 (75517 to 260440)	18388 (7819 to 35324)	19858 (9268 to 36757)	630 (234 to 1297)	366290 (169507 to 629464)	12526 (4675 to 28309)
Eastern Mediterranean	3560620 (1863280 to 5842942)	412333 (183675 to 773223)	926358 (431363 to 1694518)	29197 (12253 to 56950)	4618052 (2266815 to 7707889)	161816 (62825 to 359119)

Afghanistan	91949 (44696 to 161118)	9077 (3474 to 18741)	19898 (8602 to 38936)	588 (214 to 1249)	102633 (47629 to 179971)	3486 (1246 to 7850)
Bahrain	14222 (7646 to 22510)	1753 (835 to 3139)	4765 (2293 to 8306)	156 (67 to 300)	19623 (10073 to 31019)	704 (287 to 1542)
Djibouti	2266 (1004 to 4215)	265 (95 to 552)	807 (286 to 1730)	27 (9 to 60)	3538 (1413 to 6838)	131 (41 to 310)
Egypt	733212 (396707 to 1150359)	85811 (39891 to 154744)	182500 (88608 to 322139)	5881 (2529 to 11104)	956026 (495363 to 1510445)	34210 (13992 to 74628)
Iran (Islamic Republic of)	632255 (348083 to 1024329)	74570 (34185 to 137900)	160062 (75038 to 290751)	4902 (2251 to 9190)	794664 (398822 to 1329914)	27310 (10801 to 60742)
Iraq	244185 (127664 to 390261)	27751 (12440 to 51553)	62227 (29766 to 110485)	1939 (811 to 3739)	303851 (146488 to 493879)	10499 (4156 to 23192)
Jordan	90692 (50870 to 139286)	10425 (4976 to 18612)	23074 (11697 to 38596)	738 (327 to 1392)	114445 (60647 to 178119)	4053 (1660 to 8776)
Kuwait	47722 (27284 to 72461)	5716 (2805 to 9978)	12604 (6459 to 21108)	403 (181 to 748)	55789 (30529 to 85040)	1965 (816 to 4220)
Lebanon	36669 (19504 to 58741)	4201 (1934 to 7673)	12596 (6259 to 22215)	374 (162 to 707)	67725 (34029 to 108183)	2260 (902 to 4937)
Libya	54889 (29553 to 87265)	6412 (2994 to 11634)	15446 (7638 to 26678)	477 (207 to 916)	78567 (41058 to 125083)	2702 (1100 to 5886)
Morocco	243729 (124207 to 406199)	28687 (12699 to 54099)	64073 (29951 to 119413)	2010 (826 to 3933)	365284 (175239 to 613377)	12750 (4882 to 28577)
Oman	35941 (18959 to 57728)	4227 (1944 to 7720)	8689 (4269 to 15038)	286 (118 to 562)	30966 (16271 to 49159)	1119 (452 to 2429)
Pakistan	438392 (191479 to 825070)	48178 (16977 to 103263)	122099 (47568 to 254741)	3796 (1379 to 8242)	651312 (270421 to 1239388)	22631 (7525 to 52379)

© 2023 Chen N et al. *JAMA Network Open.*

Qatar	32486 (18187 to 49740)	3863 (1890 to 6822)	8625 (4235 to 14425)	281 (119 to 546)	26481 (14145 to 40885)	943 (385 to 2023)
Saudi Arabia	336912 (186462 to 521652)	40143 (19391 to 71193)	87163 (43954 to 149223)	2813 (1222 to 5238)	358678 (190902 to 571282)	12821 (5321 to 27665)
Sudan	169081 (86870 to 284040)	18923 (8178 to 36402)	39502 (17567 to 73760)	1248 (490 to 2552)	194836 (93113 to 329586)	6818 (2572 to 15313)
Syrian Arab Republic	93323 (47415 to 154936)	10986 (4904 to 20804)	27273 (12656 to 50313)	860 (363 to 1675)	156930 (76143 to 256009)	5523 (2153 to 12307)
Tunisia	86344 (44218 to 142032)	10252 (4561 to 19171)	26927 (12570 to 49146)	828 (343 to 1615)	155210 (75974 to 252717)	5323 (2088 to 11825)
United Arab Emirates	109433 (62110 to 170933)	13819 (6830 to 24108)	32997 (16130 to 56646)	1105 (472 to 2106)	105188 (56774 to 164291)	3843 (1570 to 8218)
Yemen	66918 (30362 to 120066)	7277 (2672 to 15115)	15030 (5818 to 30868)	484 (164 to 1077)	76307 (31784 to 142701)	2723 (877 to 6301)
Europe	9300538 (4900638 to 15251798)	1120436 (508972 to 2069504)	3077117 (1405687 to 5731746)	89258 (38516 to 169528)	17248229 (8339493 to 29272616)	587678 (230484 to 1303807)
Albania	26407 (12977 to 45618)	3236 (1406 to 6161)	4927 (2269 to 9286)	150 (60 to 295)	40066 (19475 to 66975)	1353 (530 to 2987)
Andorra	816 (411 to 1369)	100 (44 to 189)	367 (158 to 698)	11 (4 to 21)	1575 (720 to 2788)	56 (21 to 124)
Armenia	26580 (14287 to 42959)	3202 (1475 to 5912)	7284 (3585 to 12991)	223 (96 to 433)	38918 (19489 to 65123)	1335 (530 to 2982)
Austria	67836 (34252 to 116615)	8159 (3497 to 15605)	36031 (15443 to 70337)	1029 (415 to 2037)	171285 (80272 to 302307)	5961 (2236 to 13361)
Azerbaijan	75586 (38133 to 124274)	9204 (4122 to 17114)	18403 (8674 to 33880)	598 (248 to 1189)	94139 (46068 to 160613)	3433 (1350 to 7588)
Belarus	95322 (51496 to 155727)	11635 (5323 to 21430)	25293 (12584 to 45573)	752 (325 to 1435)	175154 (87246 to 291913)	5972 (2383 to 13382)

© 2023 Chen N et al. *JAMA Network Open.*

Belgium	96614 (49230 to 164797)	11479 (4900 to 22019)	43424 (18401 to 85261)	1219 (492 to 2396)	208002 (97220 to 366698)	7052 (2634 to 15729)
Bosnia and Herzegovina	33223 (16940 to 56027)	4112 (1845 to 7706)	6990 (3281 to 12984)	210 (86 to 411)	58493 (29023 to 97733)	1963 (772 to 4344)
Bulgaria	82408 (43028 to 135414)	10270 (4694 to 18800)	17865 (8513 to 32754)	527 (219 to 1016)	146158 (73580 to 238769)	4839 (1947 to 10815)
Croatia	52741 (28282 to 86576)	6473 (2990 to 11856)	11226 (5416 to 20239)	326 (139 to 624)	91670 (46213 to 147351)	2982 (1195 to 6525)
Cyprus	9185 (4383 to 16379)	1126 (454 to 2214)	3601 (1463 to 7293)	108 (41 to 219)	16462 (7137 to 29885)	594 (213 to 1355)
Czechia	133518 (69846 to 217396)	16599 (7773 to 30078)	28703 (14294 to 50338)	840 (358 to 1588)	233603 (120114 to 371208)	7609 (3091 to 16432)
Denmark	58431 (29638 to 99832)	6947 (3002 to 13266)	21661 (9037 to 42528)	627 (251 to 1236)	99693 (46516 to 175757)	3498 (1310 to 7864)
Estonia	15375 (8753 to 24224)	1809 (858 to 3236)	4633 (2393 to 8222)	133 (58 to 248)	29930 (15576 to 48657)	994 (401 to 2188)
Finland	47064 (24067 to 78909)	5543 (2397 to 10451)	23920 (10351 to 46809)	671 (276 to 1307)	118767 (56321 to 203692)	3986 (1525 to 8903)
France	575659 (300937 to 956819)	68036 (30007 to 127827)	247495 (104995 to 484687)	6989 (2836 to 13669)	1246588 (593345 to 2141999)	41677 (15689 to 93742)
Georgia	27213 (14479 to 45551)	3155 (1420 to 5900)	9180 (4304 to 16891)	267 (109 to 525)	48457 (23439 to 84226)	1598 (620 to 3556)
Germany	948506 (489442 to 1580790)	115090 (51686 to 213675)	409168 (177230 to 778329)	11588 (4832 to 22122)	2005598 (991329 to 3354211)	66826 (25786 to 149272)
Greece	95371 (47244 to 164086)	11372 (4851 to 21781)	49340 (20797 to 94899)	1366 (558 to 2689)	233815 (110079 to 394846)	7530 (2830 to 16947)
Hungary	130491 (73149 to 206859)	15887 (7514 to 28697)	26438 (13365 to 46588)	770 (331 to 1446)	217750 (113733 to 348403)	7108 (2878 to 15459)

© 2023 Chen N et al. *JAMA Network Open.*

Iceland	3372 (1746 to 5581)	400 (177 to 751)	1370 (608 to 2597)	40 (17 to 76)	6003 (2744 to 10553)	216 (84 to 480)
Ireland	49265 (25120 to 82601)	5859 (2585 to 10902)	17690 (7795 to 33705)	520 (215 to 999)	82380 (39729 to 140403)	2866 (1109 to 6449)
Israel	70215 (34508 to 119585)	8278 (3504 to 15915)	25176 (10909 to 48218)	737 (298 to 1439)	121586 (56332 to 213917)	4301 (1630 to 9680)
Italy	512845 (241981 to 909900)	62556 (25848 to 121365)	223152 (93731 to 439277)	6233 (2644 to 11989)	1221241 (565514 to 2156283)	40703 (15359 to 91810)
Kazakhstan	142810 (77266 to 226533)	16848 (7803 to 30845)	36378 (17898 to 64412)	1144 (485 to 2217)	189759 (95091 to 316181)	6635 (2668 to 14578)
Kyrgyzstan	30430 (15149 to 52143)	3504 (1497 to 6811)	7068 (3192 to 13406)	226 (87 to 459)	35263 (16268 to 62264)	1250 (472 to 2866)
Latvia	23065 (12888 to 36736)	2760 (1302 to 4993)	6529 (3216 to 11585)	187 (80 to 354)	44578 (22494 to 73878)	1482 (601 to 3258)
Lithuania	30947 (16009 to 51244)	3736 (1676 to 6884)	8809 (4235 to 16066)	254 (107 to 486)	62022 (30491 to 101928)	2028 (817 to 4521)
Luxembourg	5156 (2621 to 8851)	624 (271 to 1187)	2258 (987 to 4375)	66 (27 to 131)	10125 (4806 to 17412)	345 (130 to 773)
Malta	4266 (2076 to 7418)	517 (217 to 993)	1742 (707 to 3488)	50 (20 to 100)	8366 (3880 to 14717)	291 (106 to 659)
Monaco	460 (233 to 775)	55 (25 to 102)	220 (100 to 414)	6 (3 to 12)	1087 (522 to 1823)	37 (14 to 82)
Montenegro	7540 (4055 to 12174)	927 (430 to 1696)	1428 (697 to 2530)	43 (18 to 83)	11517 (5905 to 18387)	389 (158 to 857)
Netherlands	134076 (64136 to 232387)	16114 (6727 to 31277)	67225 (27424 to 135185)	1964 (780 to 3869)	332864 (152982 to 579915)	11385 (4204 to 25851)
North Macedonia	21849 (11317 to 35976)	2703 (1232 to 4958)	4528 (2193 to 8339)	140 (58 to 270)	34862 (17668 to 57037)	1195 (482 to 2610)
Norway	36828 (18055 to 63866)	4338 (1827 to 8462)	15609 (6386 to 31103)	445 (181 to 875)	78580 (35357 to 140709)	2745 (1008 to 6218)

© 2023 Chen N et al. *JAMA Network Open.*

Poland	475528 (256587 to 768822)	58226 (27460 to 105567)	99947 (49731 to 177335)	2935 (1392 to 5271)	784622 (408542 to 1249606)	25876 (10503 to 56232)
Portugal	106018 (50928 to 184884)	12710 (5377 to 24431)	43734 (17875 to 87242)	1229 (491 to 2437)	215362 (97405 to 385742)	7347 (2737 to 16798)
Republic of Moldova	36719 (19417 to 59303)	4490 (2072 to 8226)	9544 (4635 to 17471)	287 (121 to 559)	65915 (32956 to 109651)	2279 (907 to 5048)
Romania	244640 (143898 to 384876)	29756 (14352 to 53244)	50337 (25851 to 88618)	1472 (633 to 2746)	397953 (210842 to 632656)	13021 (5379 to 28356)
Russian Federation	1713553 (953477 to 2718512)	207111 (97896 to 372333)	426926 (212011 to 759289)	12681 (6043 to 23062)	2646151 (1259737 to 4565082)	95648 (38770 to 210233)
San Marino	335 (171 to 558)	40 (18 to 74)	146 (64 to 275)	4 (2 to 8)	692 (333 to 1181)	23 (9 to 52)
Serbia	104108 (53589 to 168696)	12927 (5937 to 23587)	21706 (10308 to 39566)	657 (275 to 1241)	174464 (86486 to 279905)	5889 (2380 to 12814)
Slovakia	61789 (32623 to 101050)	7728 (3554 to 14128)	12587 (6199 to 22770)	380 (160 to 727)	104184 (53673 to 167990)	3487 (1389 to 7622)
Slovenia	25251 (13476 to 40720)	3061 (1427 to 5580)	5816 (2866 to 10262)	168 (72 to 319)	45647 (23435 to 73039)	1461 (587 to 3186)
Spain	360956 (185577 to 606277)	43697 (19477 to 81493)	210709 (92977 to 396450)	5950 (2483 to 11408)	1012743 (423841 to 1934282)	34181 (13137 to 76217)
Sweden	86434 (43704 to 145525)	10147 (4444 to 19321)	38299 (16870 to 74026)	1051 (443 to 2005)	168251 (77567 to 291581)	5484 (2067 to 12386)
Switzerland	78780 (38484 to 137132)	9488 (4043 to 18201)	31294 (12633 to 62056)	893 (348 to 1781)	148527 (67930 to 261127)	5028 (1841 to 11430)
Tajikistan	22426 (9791 to 42474)	2555 (906 to 5472)	4909 (1791 to 10687)	161 (50 to 364)	23506 (9382 to 46122)	868 (281 to 2042)

© 2023 Chen N et al. *JAMA Network Open.*

Turkey	834448 (443730 to 1311407)	98759 (45602 to 180426)	223524 (110106 to 387268)	6823 (2964 to 12987)	1360677 (683969 to 2168981)	45887 (18261 to 100717)
Turkmenistan	33653 (17855 to 54782)	3954 (1822 to 7345)	8302 (4053 to 14814)	266 (112 to 517)	39790 (20218 to 65955)	1394 (548 to 3078)
Ukraine	508052 (284467 to 808395)	63130 (29428 to 114349)	124081 (61204 to 222142)	3696 (1578 to 6995)	851511 (435021 to 1395905)	29065 (11849 to 64261)
United Kingdom	758847 (383385 to 1258988)	88845 (40316 to 164831)	309918 (141692 to 572290)	8805 (4048 to 16163)	1505220 (734024 to 2518609)	51622 (20429 to 113655)
Uzbekistan	177530 (91344 to 293409)	21160 (9466 to 39838)	40211 (18190 to 73900)	1341 (545 to 2669)	186657 (87458 to 326641)	6882 (2630 to 15430)
Southeast Asia	4736245 (2336292 to 8245158)	553212 (224985 to 1093453)	1820023 (741420 to 3736221)	57548 (22306 to 118739)	10030775 (4241270 to 18468603)	349076 (116302 to 793237)
Bangladesh	300385 (124528 to 577032)	35314 (11891 to 75505)	81424 (27858 to 185193)	2601 (770 to 6117)	493413 (179142 to 1010516)	17337 (4856 to 42703)
Bhutan	2669 (1196 to 4840)	310 (118 to 635)	620 (238 to 1290)	20 (7 to 43)	4301 (1767 to 7910)	151 (51 to 352)
India	2700625 (1350094 to 4666679)	306160 (125635 to 603530)	1034895 (429656 to 2110525)	32079 (12682 to 65431)	6893718 (2945986 to 12599482)	237933 (80220 to 536557)
Indonesia	1059023 (544679 to 1804483)	131044 (55598 to 252764)	386035 (158052 to 779003)	12985 (5245 to 26358)	1308679 (562269 to 2380201)	48144 (16403 to 108251)
Maldives	1561 (714 to 2846)	194 (75 to 394)	719 (270 to 1474)	24 (8 to 53)	1916 (786 to 3645)	71 (23 to 165)
Myanmar	140088 (62012 to 255328)	16688 (6086 to 34396)	52811 (18961 to 114900)	1692 (538 to 3816)	226252 (87824 to 442249)	7962 (2404 to 18505)
Nepal	81679 (35161 to 154871)	9483 (3290 to 20250)	17244 (6148 to 37924)	549 (171 to 1240)	116071 (42750 to 229407)	4074 (1174 to 9787)

© 2023 Chen N et al. *JAMA Network Open.*

Sri Lanka	80396 (38874 to 134033)	9453 (3800 to 18362)	40013 (15462 to 84945)	1229 (444 to 2640)	159116 (64945 to 292173)	5305 (1742 to 12002)
Thailand	368384 (178576 to 641880)	44406 (18451 to 87226)	205725 (84636 to 419573)	6352 (2438 to 12998)	825560 (355314 to 1498931)	28039 (9416 to 64758)
Timor-Leste	1434 (459 to 3167)	162 (41 to 390)	536 (139 to 1394)	17 (4 to 44)	1748 (485 to 4089)	60 (13 to 158)
Western Pacific	5722976 (2229000 to 11210162)	708232 (245781 to 1512817)	4736841 (1648912 to 10488401)	146686 (51592 to 311432)	19711581 (7011087 to 39746939)	701553 (204499 to 1700672)
Australia	272644 (149934 to 429138)	31839 (15044 to 57183)	257382 (124285 to 468179)	7277 (3328 to 13366)	601563 (304723 to 983752)	21225 (8598 to 46182)
Brunei Darussalam	1614 (647 to 3111)	192 (65 to 412)	692 (240 to 1485)	23 (8 to 49)	2984 (1158 to 5826)	114 (35 to 265)
Cambodia	32993 (13084 to 65565)	3815 (1216 to 8368)	11184 (3695 to 25981)	360 (106 to 847)	42178 (14851 to 88424)	1462 (396 to 3574)
China	3684595 (1376321 to 7358638)	466592 (158990 to 1011042)	3598725 (1223341 to 8050029)	113352 (39673 to 241121)	15385544 (5402464 to 31214066)	549087 (159018 to 1338763)
Cook Islands	223 (125 to 343)	26 (12 to 46)	109 (55 to 187)	3 (1 to 6)	425 (222 to 672)	14 (6 to 30)
Fiji	7376 (4051 to 11464)	870 (415 to 1571)	3137 (1513 to 5530)	100 (44 to 186)	11887 (5854 to 19396)	419 (158 to 899)
Japan	578771 (180581 to 1255913)	68616 (19130 to 156434)	346571 (97348 to 843427)	9380 (2668 to 21601)	1699962 (514939 to 3619178)	58007 (14179 to 144185)
Kiribati	663 (318 to 1116)	80 (34 to 153)	199 (84 to 390)	7 (3 to 13)	792 (340 to 1427)	30 (10 to 68)
Lao People's Democratic Republic	18189 (8068 to 32264)	2116 (766 to 4317)	6927 (2580 to 14482)	228 (75 to 506)	22450 (8936 to 43159)	806 (244 to 1855)
Malaysia	160351 (86289 to 262295)	19049 (8377 to 35835)	74462 (33395 to 140768)	2352 (950 to 4725)	235040 (111867 to 403398)	8134 (2961 to 18313)

© 2023 Chen N et al. *JAMA Network Open.*

Marshall Islands	268 (119 to 475)	32 (13 to 64)	93 (37 to 191)	3 (1 to 7)	344 (139 to 646)	13 (4 to 30)
Micronesia (Federated States of)	729 (385 to 1175)	89 (41 to 164)	243 (114 to 456)	8 (4 to 16)	991 (473 to 1677)	38 (14 to 83)
Mongolia	15863 (7831 to 27586)	1900 (809 to 3681)	3802 (1659 to 7205)	124 (47 to 250)	17113 (7742 to 30672)	617 (226 to 1398)
Nauru	60 (30 to 99)	7 (3 to 14)	17 (8 to 33)	0.615 (0.254 to 1.195)	63 (29 to 109)	2 (1 to 5)
New Zealand	45608 (24221 to 74804)	5319 (2421 to 9908)	46629 (22874 to 80584)	1326 (618 to 2370)	98309 (48551 to 164521)	3504 (1366 to 7722)
Niue	18 (10 to 28)	2 (1 to 4)	8 (4 to 15)	0.248 (0.112 to 0.464)	33 (16 to 54)	1.108 (0.420 to 2.395)
Palau	198 (105 to 315)	24 (12 to 44)	96 (45 to 172)	3 (1 to 6)	360 (177 to 591)	13 (5 to 28)
Papua New Guinea	27699 (10654 to 54919)	3293 (1086 to 7126)	7867 (2507 to 18326)	269 (81 to 618)	29527 (10197 to 60721)	1121 (321 to 2747)
Philippines	380214 (176610 to 652374)	44081 (17501 to 86068)	132608 (53053 to 272710)	4232 (1618 to 8704)	407623 (171506 to 756769)	14123 (4705 to 31513)
Republic of Korea	254245 (92637 to 506965)	31508 (10311 to 67988)	138872 (45212 to 311627)	4236 (1330 to 9301)	742598 (255799 to 1503425)	28448 (8087 to 68224)
Samoa	1583 (841 to 2508)	183 (84 to 336)	576 (266 to 1039)	18 (8 to 35)	2089 (1007 to 3505)	74 (28 to 162)
Singapore	26051 (12515 to 45522)	3258 (1323 to 6389)	18338 (7354 to 37428)	576 (213 to 1186)	80376 (35471 to 146568)	3063 (1048 to 7016)
Solomon Islands	2637 (1162 to 4678)	319 (127 to 632)	768 (290 to 1582)	27 (10 to 57)	2809 (1119 to 5297)	109 (37 to 254)
Tonga	855 (480 to 1310)	98 (46 to 176)	300 (150 to 529)	9 (4 to 18)	1198 (599 to 1935)	41 (16 to 90)
Tuvalu	76 (37 to 126)	9 (4 to 17)	29 (12 to 57)	0.922 (0.359	110 (47 to 197)	4 (1 to 9)

© 2023 Chen N et al. *JAMA Network Open.*

				to 1.865)		
Vanuatu	1567 (743 to 2731)	181 (74 to 355)	455 (190 to 916)	15 (6 to 31)	1702 (744 to 3073)	61 (21 to 138)
Viet Nam	207885 (81201 to 414700)	24733 (7877 to 54494)	86752 (28601 to 205074)	2756 (795 to 6411)	323510 (112118 to 687884)	11021 (3014 to 27118)

Abbreviation: YLDs, years lived with disability.

Values in parentheses represent uncertainty levels derived from the sensitivity analyses.

© 2023 Chen N et al. *JAMA Network Open.*

eTable 7. Disease burden of musculoskeletal disorders attributable to high body mass index by sex, age, and World Bank income group, 2019 (number in thousands)

	Low back pain		Gout		Osteoarthritis	
	Prevalence	YLDs	Prevalence	YLDs	Prevalence	YLDs
Sex						
Male	15421 (7833 to 25970)	1726 (802 to 3113)	12849 (5642 to 24597)	397 (167 to 767)	29334 (13011 to 52633)	922 (304 to 2116)
Female	20846 (10566 to 35189)	2556 (1277 to 4446)	4098 (1769 to 7964)	111 (49 to 204)	43635 (19313 to 78557)	1634 (554 to 3797)
Age group (years)						
0-14	1067 (422 to 2242)	-	0	-	0	-
15-49	16718 (8070 to 29380)	1988 (936 to 3547)	4030 (1610 to 8383)	138 (59 to 265)	10608 (4346 to 20194)	395 (137 to 915)
50-74	14679 (7072 to 26440)	2004 (1015 to 3491)	9643 (3987 to 19847)	311 (134 to 593)	48867 (21296 to 88697)	1854 (682 to 4184)
75-84	2855 (1259 to 5450)	289 (128 to 520)	2427 (950 to 5130)	59 (23 to 113)	10073 (4520 to 17880)	306 (110 to 690)
≥85	947 (423 to 1760)	-	847 (345 to 1727)	-	3421 (1559 to 6031)	-
Income level						
High	13430 (7267 to 21523)	1578 (734 to 2859)	7940 (3842 to 13961)	226 (106 to 406)	24057 (11454 to 41340)	836 (322 to 1856)
Upper-middle	13546 (6607 to 23404)	1643 (691 to 3196)	6205 (2440 to 12854)	194 (75 to 396)	32741 (13825 to 60466)	1154 (394 to 2684)
Lower-middle	8115 (4029 to 13942)	932 (385 to 1824)	2505 (1059 to 5022)	79 (31 to 161)	14631 (6449 to 26351)	511 (179 to 1156)
Low	1177 (534 to 2142)	128 (48 to 267)	297 (115 to 621)	9 (3 to 21)	1541 (643 to 2896)	55 (18 to 129)

Abbreviation: YLDs, years lived with disability.
Values in parentheses represent uncertainty levels derived from the sensitivity analyses.
To estimate morbidity-related costs, YLDs were calculated in people aged 15 to 84 years only. The age categories were determined based on previous studies.[3,33]

© 2023 Chen N et al. *JAMA Network Open.*

eTable 8. Health-care, morbidity-related, and total costs of musculoskeletal disorders attributable to high body mass index by location in 2019

	Health-care costs		Morbidity-related costs		Total
Location	Amount (uncertainty level, US$, thousands)	% of total	Amount (uncertainty level, US$, millions)	% of total	Amount (uncertainty level, US$, millions)
Global	60485258 (30683169 to 100000000)	33.5	120000 (53073 to 233000)	66.5	181000 (83757 to 333000)
Africa	469748 (241799 to 773728)	29.3	1132 (476 to 2269)	70.7	1602 (718 to 3043)
Algeria	102601 (52124 to 165617)	30.8	231 (101 to 449)	69.2	333 (153 to 614)
Angola	4040 (1653 to 7672)	17.3	19 (6 to 42)	82.7	23 (8 to 50)
Benin	1244 (599 to 2160)	14.8	7 (3 to 15)	85.2	8 (4 to 17)
Botswana	5664 (3045 to 9145)	40.8	8 (4 to 16)	59.2	14 (7 to 25)
Burkina Faso	2365 (973 to 4530)	31.2	5 (2 to 12)	68.8	8 (3 to 16)
Burundi	404 (140 to 861)	51.1	0.387 (0.105 to 0.928)	48.9	0.791 (0.245 to 1.789)
Cabo Verde	479 (242 to 812)	30.5	1.091 (0.460 to 2.193)	69.5	2 (1 to 3)
Cameroon	5851 (2973 to 9767)	23.0	20 (8 to 39)	77.0	25 (11 to 49)
Central African Republic	140 (47 to 303)	22.0	0.497 (0.136 to 1.184)	78.0	0.637 (0.182 to 1.487)
Chad	579 (222 to 1138)	20.7	2 (1 to 5)	79.3	3 (1 to 6)
Comoros	167 (73 to 309)	30.2	0.386 (0.141 to 0.813)	69.8	0.553 (0.215 to 1.122)
Congo	713 (357 to 1207)	11.3	6 (2 to 11)	88.7	6 (3 to 13)
Democratic Republic of the Congo	2249 (901 to 4444)	20.4	9 (3 to 20)	79.6	11 (4 to 24)

© 2023 Chen N et al. *JAMA Network Open.*

Equatorial Guinea	1131 (598 to 1878)	18.5	5 (2 to 10)	81.5	6 (3 to 12)
Eritrea	158 (62 to 306)	33.3	0.317 (0.096 to 0.733)	66.7	0.476 (0.158 to 1.039)
Eswatini	1454 (764 to 2316)	38.1	2 (1 to 5)	61.9	4 (2 to 7)
Ethiopia	4130 (1586 to 8281)	28.5	10 (3 to 23)	71.5	14 (5 to 32)
Gabon	2075 (1047 to 3415)	28.3	5 (2 to 10)	71.7	7 (3 to 14)
Gambia	299 (140 to 529)	31.7	0.644 (0.253 to 1.341)	68.3	0.943 (0.393 to 1.869)
Ghana	10103 (5229 to 16795)	22.1	36 (15 to 72)	77.9	46 (20 to 88)
Guinea	1298 (561 to 2425)	21.6	5 (2 to 10)	78.4	6 (2 to 13)
Guinea-Bissau	245 (100 to 473)	47.5	0.271 (0.090 to 0.601)	52.5	0.516 (0.190 to 1.073)
Ivory Coast	5148 (2402 to 9117)	21.4	19 (7 to 39)	78.6	24 (10 to 48)
Kenya	13890 (6716 to 24326)	29.5	33 (14 to 67)	70.5	47 (20 to 91)
Lesotho	1489 (761 to 2497)	55.0	1 (1 to 2)	45.0	3 (1 to 5)
Liberia	1086 (541 to 1836)	49.7	1.099 (0.468 to 2.200)	50.3	2 (1 to 4)
Madagascar	1137 (466 to 2171)	38.5	2 (1 to 4)	61.5	3 (1 to 6)
Malawi	1505 (616 to 2869)	58.5	1.070 (0.358 to 2.328)	41.5	3 (1 to 5)
Mali	1283 (540 to 2407)	20.1	5 (2 to 11)	79.9	6 (2 to 13)
Mauritania	1524 (790 to 2536)	22.4	5 (2 to 11)	77.6	7 (3 to 13)
Mauritius	6655 (3140 to 11422)	38.7	11 (4 to 21)	61.3	17 (7 to 33)
Mozambique	1880 (787 to 3556)	40.4	3 (1 to 6)	59.6	5 (2 to 9)
Namibia	3835 (2038 to 6398)	41.6	5 (2 to 11)	58.4	9 (4 to 17)
Niger	867 (344 to 1710)	34.8	2 (1 to 4)	65.2	2 (1 to 5)

© 2023 Chen N et al. *JAMA Network Open.*

Nigeria	47880 (22444 to 85155)	11.5	368 (148 to 751)	88.5	415 (171 to 836)
Rwanda	1601 (621 to 3140)	50.7	1.557 (0.495 to 3.515)	49.3	3 (1 to 7)
Sao Tome and Principe	77 (38 to 132)	29.5	0.184 (0.076 to 0.376)	70.5	0.262 (0.114 to 0.509)
Senegal	2740 (1287 to 4827)	24.7	8 (3 to 17)	75.3	11 (4 to 22)
Seychelles	435 (208 to 748)	24.8	1 (1 to 3)	75.2	2 (1 to 3)
Sierra Leone	1578 (631 to 3060)	55.0	1.291 (0.421 to 2.887)	45.0	3 (1 to 6)
South Africa	209936 (115795 to 329451)	44.8	259 (120 to 498)	55.2	469 (236 to 828)
South Sudan	739 (339 to 1332)	38.6	1.177 (0.453 to 2.444)	61.4	2 (1 to 4)
Togo	819 (375 to 1490)	25.5	2 (1 to 5)	74.5	3 (1 to 7)
Uganda	3703 (1584 to 6937)	31.4	8 (3 to 17)	68.6	12 (4 to 24)
United Republic of Tanzania	5804 (2756 to 10188)	35.8	10 (4 to 22)	64.2	16 (7 to 32)
Zambia	3183 (1452 to 5762)	35.2	6 (2 to 12)	64.8	9 (4 to 18)
Zimbabwe	3563 (1690 to 6280)	50.6	3 (1 to 7)	49.4	7 (3 to 13)
The Americas	34375539 (18682376 to 54106795)	34.9	64049 (30761 to 117000)	65.1	98425 (49443 to 171000)
Antigua and Barbuda	407 (199 to 687)	21.9	1 (1 to 3)	78.1	2 (1 to 4)
Argentina	373909 (175597 to 651998)	47.3	417 (170 to 855)	52.7	791 (345 to 1507)
Bahamas	3717 (1885 to 6084)	22.2	13 (6 to 26)	77.8	17 (8 to 32)
Barbados	1703 (843 to 2802)	18.9	7 (3 to 15)	81.1	9 (4 to 17)
Belize	532 (272 to 850)	23.6	2 (1 to 3)	76.4	2 (1 to 4)

© 2023 Chen N et al. *JAMA Network Open.*

Bolivia (Plurinational State of)	18804 (9396 to 30889)	36.6	33 (14 to 65)	63.4	51 (23 to 96)
Brazil	1227840 (639585 to 2012663)	38.7	1948 (858 to 3837)	61.3	3176 (1497 to 5849)
Canada	1071367 (524963 to 1828023)	28.2	2732 (1217 to 5233)	71.8	3804 (1742 to 7061)
Chile	220423 (107011 to 373813)	36.9	376 (159 to 754)	63.1	597 (266 to 1128)
Colombia	206873 (99891 to 348837)	43.9	265 (109 to 536)	56.1	471 (209 to 885)
Costa Rica	31518 (15044 to 53135)	35.5	57 (24 to 116)	64.5	89 (39 to 169)
Cuba	172820 (86206 to 284847)	62.1	105 (46 to 210)	37.9	278 (132 to 495)
Dominica	215 (109 to 347)	29.6	0.511 (0.221 to 1.011)	70.4	0.725 (0.330 to 1.358)
Dominican Republic	32256 (14998 to 55194)	33.1	65 (27 to 133)	66.9	97 (42 to 188)
Ecuador	61230 (33262 to 93623)	38.3	99 (45 to 191)	61.7	160 (78 to 285)
El Salvador	14347 (6713 to 24270)	39.3	22 (9 to 45)	60.7	36 (16 to 69)
Grenada	312 (156 to 522)	21.2	1.161 (0.496 to 2.330)	78.8	1 (1 to 3)
Guatemala	18381 (8180 to 33080)	34.3	35 (13 to 75)	65.7	54 (21 to 108)
Guyana	1336 (638 to 2279)	25.8	4 (2 to 8)	74.2	5 (2 to 10)
Haiti	1127 (420 to 2272)	24.4	3 (1 to 8)	75.6	5 (1 to 10)
Honduras	7978 (3557 to 14073)	30.2	18 (7 to 39)	69.8	26 (11 to 53)

© 2023 Chen N et al. *JAMA Network Open.*

Jamaica	7745 (4028 to 12520)	29.9	18 (8 to 36)	70.1	26 (12 to 48)
Mexico	523548 (260295 to 867253)	38.7	829 (359 to 1642)	61.3	1353 (619 to 2509)
Nicaragua	7711 (3598 to 13234)	44.0	10 (4 to 20)	56.0	18 (8 to 33)
Panama	29495 (14163 to 49760)	45.3	36 (15 to 72)	54.7	65 (29 to 122)
Paraguay	18335 (9097 to 30841)	39.0	29 (12 to 58)	61.0	47 (21 to 89)
Peru	73256 (34531 to 122849)	31.3	161 (66 to 328)	68.7	234 (100 to 451)
Saint Kitts and Nevis	349 (175 to 578)	24.7	1.063 (0.456 to 2.120)	75.3	1 (1 to 3)
Saint Lucia	547 (269 to 902)	24.0	2 (1 to 3)	76.0	2 (1 to 4)
Saint Vincent and the Grenadines	267 (129 to 446)	28.0	0.688 (0.290 to 1.400)	72.0	0.955 (0.418 to 1.846)
Suriname	2875 (1373 to 4783)	48.8	3 (1 to 6)	51.2	6 (3 to 11)
Trinidad and Tobago	12996 (6768 to 20821)	27.4	34 (15 to 68)	72.6	47 (22 to 89)
United States of America	30176215 (16592947 to 47067700)	34.8	56525 (27491 to 102000)	65.2	86702 (44084 to 149000)
Uruguay	33712 (15984 to 58222)	37.3	57 (23 to 115)	62.7	90 (39 to 173)
Venezuela (Bolivarian Republic of)	21393 (10093 to 36598)	13.4	138 (56 to 282)	86.6	159 (66 to 319)
Eastern Mediterranean	1475227 (778959 to 2368993)	37.5	2454 (1107 to 4671)	62.5	3930 (1886 to 7040)
Afghanistan	8052 (3820 to 14144)	51.5	8 (3 to 16)	48.5	16 (7 to 30)

© 2023 Chen N et al. *JAMA Network Open.*

Bahrain	14188 (7424 to 22499)	35.8	25 (12 to 48)	64.2	40 (19 to 71)
Djibouti	94 (39 to 180)	10.4	0.816 (0.279 to 1.771)	89.6	0.910 (0.318 to 1.951)
Egypt	192551 (101676 to 303942)	39.8	291 (131 to 554)	60.2	484 (233 to 858)
Iran (Islamic Republic of)	293735 (153923 to 485077)	44.6	365 (162 to 707)	55.4	659 (316 to 1192)
Iraq	42128 (21115 to 68065)	27.9	109 (47 to 210)	72.1	151 (69 to 278)
Jordan	27871 (15161 to 43176)	39.6	43 (20 to 80)	60.4	70 (35 to 123)
Kuwait	52420 (29270 to 79927)	34.0	102 (48 to 187)	66.0	154 (77 to 267)
Lebanon	31771 (16311 to 50922)	44.7	39 (17 to 76)	55.3	71 (34 to 127)
Libya	28425 (15034 to 45308)	32.0	60 (27 to 116)	68.0	89 (42 to 161)
Morocco	48012 (23633 to 80533)	25.4	141 (60 to 280)	74.6	189 (84 to 361)
Oman	16014 (8420 to 25644)	17.4	76 (34 to 144)	82.6	92 (42 to 169)
Pakistan	31688 (13433 to 60136)	30.3	73 (25 to 160)	69.7	104 (39 to 220)
Qatar	33638 (18424 to 51801)	33.0	68 (32 to 126)	67.0	102 (51 to 178)
Saudi Arabia	418421 (226947 to 658117)	38.1	681 (318 to 1265)	61.9	1099 (545 to 1923)
Sudan	17017 (8421 to 28747)	47.4	19 (8 to 38)	52.6	36 (16 to 67)

© 2023 Chen N et al. *JAMA Network Open.*

Syrian Arab Republic	47950 (23683 to 78980)	68.8	22 (9 to 43)	31.2	70 (33 to 122)
Tunisia	39047 (19433 to 63984)	43.4	51 (22 to 101)	56.6	90 (41 to 165)
United Arab Emirates	129416 (71580 to 202690)	32.1	273 (129 to 501)	67.9	403 (201 to 703)
Yemen	2790 (1212 to 5122)	26.1	8 (3 to 17)	73.9	11 (4 to 22)
Europe	16315969 (8023944 to 27700038)	32.2	34278 (14561 to 68228)	67.8	50594 (22585 to 95928)
Albania	9907 (4835 to 16825)	36.0	18 (7 to 35)	64.0	28 (12 to 52)
Andorra	1381 (654 to 2403)	20.2	5 (2 to 11)	79.8	7 (3 to 13)
Armenia	16726 (8638 to 27613)	52.9	15 (7 to 29)	47.1	32 (15 to 57)
Austria	267231 (127896 to 469299)	34.0	518 (211 to 1062)	66.0	785 (339 to 1531)
Azerbaijan	17274 (8570 to 29013)	31.3	38 (16 to 74)	68.7	55 (25 to 103)
Belarus	57289 (29451 to 94898)	44.5	71 (31 to 140)	55.5	129 (61 to 235)
Belgium	317375 (152524 to 555013)	28.8	784 (320 to 1589)	71.2	1102 (472 to 2144)
Bosnia and Herzegovina	23610 (11832 to 39655)	53.3	21 (9 to 41)	46.7	44 (21 to 81)
Bulgaria	78794 (40209 to 129241)	44.8	97 (43 to 189)	55.2	176 (83 to 318)
Croatia	53070 (27408 to 86166)	31.7	114 (51 to 221)	68.3	167 (78 to 307)

© 2023 Chen N et al. *JAMA Network Open.*

Cyprus	8869 (3987 to 16040)	25.3	26 (10 to 54)	74.7	35 (14 to 70)
Czechia	220108 (113894 to 353663)	35.2	406 (183 to 778)	64.8	626 (297 to 1131)
Denmark	177858 (85611 to 310644)	25.0	534 (221 to 1074)	75.0	712 (307 to 1385)
Estonia	21637 (11645 to 34840)	30.9	48 (22 to 93)	69.1	70 (33 to 128)
Finland	141277 (68443 to 241549)	27.1	381 (158 to 769)	72.9	522 (226 to 1010)
France	1864938 (914829 to 3180197)	32.7	3837 (1602 to 7691)	67.3	5702 (2517 to 10871)
Georgia	15711 (7886 to 26955)	52.7	14 (6 to 28)	47.3	30 (14 to 55)
Germany	3500680 (1751045 to 5868102)	33.0	7112 (3038 to 14100)	67.0	10613 (4789 to 19968)
Greece	153155 (73067 to 261142)	37.5	255 (104 to 520)	62.5	408 (177 to 781)
Hungary	147198 (79118 to 234976)	35.1	273 (124 to 521)	64.9	420 (203 to 756)
Iceland	9020 (4330 to 15536)	19.5	37 (16 to 74)	80.5	46 (20 to 90)
Ireland	154160 (75885 to 261802)	23.9	491 (209 to 970)	76.1	645 (285 to 1232)
Israel	107703 (51006 to 187530)	18.6	471 (193 to 952)	81.4	578 (244 to 1139)
Italy	1234719 (573944 to 2189411)	29.9	2890 (1153 to 5940)	70.1	4125 (1727 to 8130)
Kazakhstan	52510 (27259 to 85689)	25.1	157 (70 to 303)	74.9	209 (97 to 388)

© 2023 Chen N et al. *JAMA Network Open.*

Kyrgyzstan	4265 (2043 to 7433)	46.0	5 (2 to 10)	54.0	9 (4 to 18)
Latvia	24710 (12955 to 40418)	33.0	50 (23 to 97)	67.0	75 (36 to 138)
Lithuania	43268 (21662 to 71437)	38.7	69 (30 to 136)	61.3	112 (51 to 207)
Luxembourg	18053 (8769 to 31131)	19.2	76 (31 to 153)	80.8	94 (40 to 184)
Malta	11117 (5232 to 19543)	41.2	16 (6 to 32)	58.8	27 (12 to 52)
Monaco	981 (478 to 1654)	6.9	13 (6 to 26)	93.1	14 (6 to 28)
Montenegro	6404 (3349 to 10288)	47.1	7 (3 to 14)	52.9	14 (7 to 24)
Netherlands	528673 (245312 to 923365)	29.4	1269 (507 to 2620)	70.6	1798 (753 to 3543)
North Macedonia	11769 (6015 to 19342)	45.8	14 (6 to 27)	54.2	26 (12 to 46)
Norway	147834 (68406 to 262604)	28.3	374 (151 to 769)	71.7	522 (219 to 1032)
Poland	518254 (273644 to 831836)	38.2	837 (380 to 1601)	61.8	1356 (654 to 2433)
Portugal	196985 (90833 to 350543)	34.7	371 (150 to 759)	65.3	568 (241 to 1109)
Republic of Moldova	12476 (6373 to 20557)	28.4	31 (14 to 61)	71.6	44 (20 to 82)
Romania	213148 (117969 to 337940)	39.6	325 (150 to 616)	60.4	538 (268 to 953)
Russian Federation	1235684 (630428 to 2060977)	35.3	2263 (1025 to 4341)	64.7	3499 (1656 to 6402)
San Marino	868 (425 to 1472)	34.3	2 (1 to 3)	65.7	3 (1 to 5)

© 2023 Chen N et al. *JAMA Network Open.*

Serbia	66068 (33237 to 106631)	39.2	102 (45 to 197)	60.8	168 (79 to 304)
Slovakia	71701 (37283 to 116469)	34.6	136 (60 to 262)	65.4	207 (97 to 379)
Slovenia	44554 (23207 to 71615)	33.6	88 (39 to 170)	66.4	133 (63 to 241)
Spain	995177 (444074 to 1835036)	34.4	1895 (798 to 3807)	65.6	2890 (1242 to 5642)
Sweden	287676 (137106 to 494988)	30.8	645 (270 to 1301)	69.2	933 (407 to 1796)
Switzerland	355331 (166127 to 624437)	24.0	1125 (457 to 2291)	76.0	1481 (623 to 2915)
Tajikistan	2146 (896 to 4144)	47.4	2 (1 to 5)	52.6	5 (2 to 9)
Turkey	529015 (272079 to 839811)	43.9	676 (299 to 1309)	56.1	1205 (571 to 2149)
Turkmenistan	16993 (8812 to 27966)	33.5	34 (15 to 65)	66.5	51 (24 to 93)
Ukraine	237586 (125978 to 385497)	46.6	273 (122 to 527)	53.4	510 (248 to 912)
United Kingdom	2056518 (1014245 to 3439575)	29.4	4928 (2149 to 9699)	70.6	6985 (3163 to 13139)
Uzbekistan	26517 (13041 to 45128)	42.9	35 (15 to 69)	57.1	62 (28 to 115)
Southeast Asia	838003 (376782 to 1510251)	27.0	2265 (873 to 4695)	73.0	3103 (1249 to 6206)
Bangladesh	18750 (7191 to 37516)	19.9	75 (24 to 168)	80.1	94 (31 to 206)
Bhutan	401 (171 to 735)	29.0	0.982 (0.361 to 2.095)	71.0	1 (1 to 3)

© 2023 Chen N et al. *JAMA Network Open.*

India	438755 (197106 to 790424)	26.5	1215 (463 to 2533)	73.5	1654 (660 to 3324)
Indonesia	165095 (77387 to 292346)	25.2	491 (199 to 983)	74.8	656 (277 to 1275)
Maldives	1405 (606 to 2629)	47.0	2 (1 to 3)	53.0	3 (1 to 6)
Myanmar	15758 (6453 to 30048)	39.1	25 (8 to 53)	60.9	40 (15 to 83)
Nepal	6822 (2698 to 13265)	45.0	8 (3 to 18)	55.0	15 (5 to 32)
Sri Lanka	24480 (10634 to 43687)	39.3	38 (14 to 78)	60.7	62 (25 to 121)
Thailand	166402 (74496 to 299292)	28.9	410 (159 to 856)	71.1	577 (234 to 1155)
Timor-Leste	135 (40 to 308)	34.0	0.262 (0.064 to 0.641)	66.0	0.396 (0.104 to 0.949)
Western Pacific	7010773 (2579309 to 13993379)	30.4	16055 (5296 to 35929)	69.6	23065 (7875 to 49923)
Australia	886300 (460357 to 1440282)	26.4	2468 (1109 to 4761)	73.6	3355 (1570 to 6202)
Brunei Darussalam	1230 (482 to 2397)	15.9	7 (2 to 14)	84.1	8 (3 to 17)
Cambodia	3262 (1214 to 6694)	39.5	5 (2 to 11)	60.5	8 (3 to 18)
China	3085173 (1096550 to 6261247)	29.5	7382 (2354 to 16936)	70.5	10467 (3451 to 23197)
Cook Islands	126 (68 to 198)	15.4	0.692 (0.315 to 1.313)	84.6	0.818 (0.383 to 1.511)
Fiji	1777 (915 to 2851)	20.0	7 (3 to 14)	80.0	9 (4 to 16)
Japan	1980091 (603502 to 4254798)	34.0	3850 (1022 to 9114)	66.0	5830 (1626 to 13369)

© 2023 Chen N et al. *JAMA Network Open.*

Kiribati	70 (32 to 123)	22.8	0.238 (0.097 to 0.475)	77.2	0.308 (0.129 to 0.597)
Lao People's Democratic Republic	1537 (643 to 2855)	19.1	7 (2 to 14)	80.9	8 (3 to 17)
Malaysia	88582 (44422 to 149520)	33.5	176 (74 to 348)	66.5	264 (118 to 498)
Marshall Islands	57 (24 to 104)	25.0	0.170 (0.066 to 0.349)	75.0	0.227 (0.090 to 0.453)
Micronesia (Federated States of)	42 (21 to 69)	9.2	0.413 (0.181 to 0.798)	90.8	0.454 (0.202 to 0.868)
Mongolia	3826 (1809 to 6762)	35.8	7 (3 to 14)	64.2	11 (5 to 21)
Nauru	32 (15 to 54)	32.2	0.067 (0.029 to 0.130)	67.8	0.099 (0.044 to 0.184)
New Zealand	120569 (60995 to 200832)	29.9	283 (123 to 556)	70.1	403 (184 to 757)
Niue	8 (4 to 13)	20.9	0.030 (0.013 to 0.058)	79.1	0.038 (0.017 to 0.070)
Palau	187 (95 to 305)	31.2	0.412 (0.184 to 0.789)	68.8	0.599 (0.279 to 1.093)
Papua New Guinea	1135 (414 to 2298)	9.3	11 (4 to 25)	90.7	12 (4 to 27)
Philippines	58665 (25937 to 105115)	32.7	121 (46 to 243)	67.3	180 (72 to 348)
Republic of Korea	637154 (222663 to 1288195)	31.4	1393 (429 to 3152)	68.6	2030 (651 to 4440)
Samoa	218 (110 to 358)	16.4	1.111 (0.487 to 2.125)	83.6	1 (1 to 2)
Singapore	89953 (40509 to 162850)	25.9	258 (97 to 542)	74.1	348 (138 to 704)
Solomon Islands	118 (50 to 217)	12.2	0.851 (0.329 to 1.745)	87.8	0.969 (0.378 to 1.962)
Tonga	110 (58 to 175)	11.4	0.855 (0.388 to 1.612)	88.6	0.966 (0.446 to 1.787)
Tuvalu	29 (13 to 50)	39.9	0.043 (0.018 to 0.086)	60.1	0.072 (0.031 to 0.137)

© 2023 Chen N et al. *JAMA Network Open.*

Vanuatu	56 (26 to 100)	6.1	0.871 (0.346 to 1.758)	93.9	0.927 (0.372 to 1.858)
Viet Nam	50464 (18382 to 104917)	39.9	76 (23 to 174)	60.1	127 (41 to 279)

Values in parentheses represent uncertainty levels derived from the sensitivity analyses.

Percentage calculation was based on the mean values.

© 2023 Chen N et al. *JAMA Network Open.*

eTable 9. Health-care costs of low back pain attributable to high body mass index by location in 2019

Location	Health-care costs per capita, 2019 ($PPP)	Spending ratio	Health-care costs per case, 2019 (US$)	Prevalence (uncertainty level)	Health-care costs (uncertainty level, US$, thousands)
Global	3073	0.271	602	36267110 (18437198 to 61011482)	23113028 (12772057 to 36371680)
Africa	318	0.028	62	2759627 (1375035 to 4752062)	186351 (100010 to 303177)
Algeria	765	0.067	150	285676 (151890 to 457322)	42811 (22762 to 68534)
Angola	195	0.017	38	48771 (21260 to 90401)	1863 (812 to 3453)
Benin	79	0.007	15	36435 (18325 to 62022)	564 (284 to 960)
Botswana	1144	0.101	224	9249 (5394 to 14363)	2073 (1209 to 3219)
Burkina Faso	119	0.010	23	44535 (19388 to 84036)	1038 (452 to 1959)
Burundi	86	0.008	17	11058 (3935 to 23095)	186 (66 to 389)
Cabo Verde	380	0.033	74	2389 (1279 to 3953)	178 (95 to 294)
Cameroon	121	0.011	24	113130 (59622 to 187541)	2682 (1413 to 4445)
Central African Republic	61	0.005	12	5456 (1949 to 11553)	65 (23 to 138)
Chad	71	0.006	14	19903 (8112 to 38096)	277 (113 to 530)
Comoros	187	0.016	37	1914 (915 to 3382)	70 (34 to 124)
Congo	93	0.008	18	16659 (9035 to 27335)	303 (165 to 498)
Democratic Republic of the Congo	43	0.004	8	119975 (49724 to 230839)	1011 (419 to 1944)
Equatorial Guinea	554	0.049	109	5208 (2884 to 8445)	565 (313 to 916)
Eritrea	46	0.004	9	6813 (2901 to 12837)	61 (26 to 116)
Eswatini	593	0.052	116	4682 (2551 to 7349)	544 (296 to 854)
Ethiopia	75	0.007	15	133552 (54173 to 259601)	1962 (796 to 3814)
Gabon	541	0.048	106	8095 (4269 to 13062)	858 (452 to 1384)
Gambia	118	0.010	23	5660 (2791 to 9833)	131 (65 to 227)
Ghana	189	0.017	37	102261 (56419 to 164425)	3786 (2089 to 6088)
Guinea	124	0.011	24	23309 (10838 to 42330)	566 (263 to 1028)
Guinea-Bissau	178	0.016	35	3196 (1380 to 6084)	111 (48 to 212)
Ivory Coast	170	0.015	33	71045 (35071 to	2366 (1168 to 4097)

© 2023 Chen N et al. *JAMA Network Open.*

				123014)	
Kenya	248	0.022	49	131085 (67420 to 223807)	6368 (3275 to 10873)
Lesotho	390	0.034	76	7317 (3920 to 11862)	559 (299 to 906)
Liberia	154	0.014	30	15975 (8388 to 26430)	482 (253 to 797)
Madagascar	70	0.006	14	35993 (15906 to 65940)	494 (218 to 904)
Malawi	107	0.009	21	32689 (14077 to 60688)	685 (295 to 1272)
Mali	80	0.007	16	34026 (15244 to 63136)	533 (239 to 989)
Mauritania	210	0.019	41	15075 (8171 to 24772)	620 (336 to 1019)
Mauritius	1553	0.137	304	7722 (4018 to 12908)	2349 (1222 to 3927)
Mozambique	100	0.009	20	46292 (20638 to 84062)	907 (404 to 1647)
Namibia	964	0.085	189	7728 (4417 to 12356)	1459 (834 to 2333)
Niger	71	0.006	14	28472 (11978 to 54802)	396 (167 to 762)
Nigeria	192	0.017	38	630730 (314374 to 1087273)	23723 (11824 to 40894)
Rwanda	153	0.013	30	23517 (9601 to 45011)	705 (288 to 1349)
Sao Tome and Principe	234	0.021	46	657 (336 to 1099)	30 (15 to 50)
Senegal	158	0.014	31	35206 (18004 to 60893)	1090 (557 to 1885)
Seychelles	1440	0.127	282	598 (308 to 1009)	169 (87 to 285)
Sierra Leone	241	0.021	47	14583 (6174 to 27854)	688 (291 to 1315)
South Africa	1202	0.106	235	310618 (179043 to 479891)	73139 (42158 to 112997)
South Sudan	83	0.007	16	20595 (9933 to 36251)	335 (162 to 589)
Togo	96	0.008	19	18228 (9036 to 32045)	343 (170 to 603)
Uganda	131	0.012	26	68074 (30470 to 123762)	1747 (782 to 3176)
United Republic of Tanzania	103	0.009	20	121912 (62482 to 208169)	2460 (1261 to 4200)
Zambia	205	0.018	40	35991 (17353 to 63778)	1445 (697 to 2561)
Zimbabwe	211	0.019	41	37572 (19639 to 63344)	1553 (812 to 2618)
The Americas	7659	0.675	1500	10187105 (5732953 to 15709360)	14331710 (8440629 to 21274325)
Antigua and Barbuda	1281	0.113	251	503 (260 to 830)	126 (65 to 208)
Argentina	2285	0.201	448	262329 (126688 to 451237)	117423 (56708 to 201981)
Bahamas	2468	0.218	483	2474 (1330 to 3950)	1196 (643 to 1910)
Barbados	1020	0.090	200	2376 (1242 to 3857)	475 (248 to 771)
Belize	431	0.038	84	2404 (1303 to 3762)	203 (110 to 318)
Bolivia (Plurinational State of)	585	0.052	115	57317 (29490 to 94187)	6568 (3379 to 10794)

© 2023 Chen N et al. *JAMA Network Open.*

Brazil	1443	0.127	283	1520278 (816496 to 2456595)	429745 (230803 to 694418)
Canada	5837	0.514	1143	391284 (207213 to 632944)	447407 (236934 to 723729)
Chile	2472	0.218	484	142440 (71391 to 237419)	68976 (34571 to 114970)
Colombia	1214	0.107	238	311577 (156798 to 520706)	74098 (37289 to 123832)
Costa Rica	1798	0.158	352	30115 (15053 to 49822)	10607 (5302 to 17548)
Cuba	2863	0.252	561	91468 (48985 to 145803)	51299 (27473 to 81773)
Dominica	676	0.060	132	498 (266 to 793)	66 (35 to 105)
Dominican Republic	1124	0.099	220	49943 (24641 to 84538)	10997 (5426 to 18614)
Ecuador	963	0.085	189	100824 (58008 to 151615)	19020 (10943 to 28602)
El Salvador	707	0.062	138	37121 (18393 to 61714)	5141 (2547 to 8547)
Grenada	863	0.076	169	606 (321 to 994)	102 (54 to 168)
Guatemala	601	0.053	118	65306 (30099 to 117189)	7689 (3544 to 13797)
Guyana	648	0.057	127	3748 (1897 to 6286)	476 (241 to 798)
Haiti	111	0.010	22	21035 (8248 to 41345)	457 (179 to 899)
Honduras	429	0.038	84	38506 (17742 to 67247)	3236 (1491 to 5651)
Jamaica	715	0.063	140	18673 (10173 to 29589)	2615 (1425 to 4144)
Mexico	1171	0.103	229	806363 (416624 to 1313358)	184973 (95570 to 301274)
Nicaragua	501	0.044	98	31043 (14981 to 52750)	3047 (1470 to 5177)
Panama	2353	0.207	461	21388 (10729 to 35559)	9859 (4946 to 16390)
Paraguay	968	0.085	190	37334 (19362 to 62046)	7080 (3672 to 11766)
Peru	683	0.060	134	176617 (85854 to 293398)	23631 (11487 to 39255)
Saint Kitts and Nevis	1415	0.125	277	393 (207 to 642)	109 (57 to 178)
Saint Lucia	744	0.066	146	1201 (626 to 1952)	175 (91 to 285)
Saint Vincent and the Grenadines	626	0.055	123	677 (343 to 1113)	83 (42 to 137)
Suriname	1420	0.125	278	3316 (1682 to 5443)	922 (468 to 1514)
Trinidad and Tobago	1836	0.162	360	10855 (5963 to 17025)	3904 (2145 to 6123)
United States of America	11345	1.000	2222	5769970 (3443558 to 8463897)	12823282 (7653024 to 18810311)
Uruguay	2156	0.190	422	23172 (11469 to 39315)	9786 (4844 to 16604)

© 2023 Chen N et al. *JAMA Network Open.*

Venezuela (Bolivarian Republic of)	230	0.020	45	153952 (75517 to 260440)	6936 (3402 to 11734)
Eastern Mediterranean	832	0.073	163	3560620 (1863280 to 5842942)	706804 (385241 to 1121446)
Afghanistan	223	0.020	44	91949 (44696 to 161118)	4017 (1953 to 7038)
Bahrain	2260	0.199	443	14222 (7646 to 22510)	6296 (3385 to 9966)
Djibouti	88	0.008	17	2266 (1004 to 4215)	39 (17 to 73)
Egypt	617	0.054	121	733212 (396707 to 1150359)	88621 (47949 to 139040)
Iran (Islamic Republic of)	1112	0.098	218	632255 (348083 to 1024329)	137727 (75824 to 223134)
Iraq	415	0.037	81	244185 (127664 to 390261)	19851 (10379 to 31727)
Jordan	734	0.065	144	90692 (50870 to 139286)	13040 (7314 to 20027)
Kuwait	2724	0.240	534	47722 (27284 to 72461)	25465 (14559 to 38666)
Lebanon	1663	0.147	326	36669 (19504 to 58741)	11946 (6354 to 19136)
Libya	1155	0.102	226	54889 (29553 to 87265)	12419 (6687 to 19744)
Morocco	429	0.038	84	243729 (124207 to 406199)	20483 (10438 to 34136)
Oman	1272	0.112	249	35941 (18959 to 57728)	8956 (4724 to 14384)
Pakistan	158	0.014	31	438392 (191479 to 825070)	13569 (5927 to 25537)
Qatar	3017	0.266	591	32486 (18187 to 49740)	19200 (10749 to 29397)
Saudi Arabia	3223	0.284	631	336912 (186462 to 521652)	212715 (117726 to 329354)
Sudan	252	0.022	49	169081 (86870 to 284040)	8347 (4288 to 14022)
Syrian Arab Republic	1046	0.092	205	93323 (47415 to 154936)	19122 (9716 to 31747)
Tunisia	884	0.078	173	86344 (44218 to 142032)	14952 (7657 to 24596)
United Arab Emirates	3203	0.282	627	109433 (62110 to 170933)	68663 (38971 to 107252)
Yemen	105	0.009	21	66918 (30362 to 120066)	1376 (625 to 2470)
Europe	3443	0.303	674	9300538 (4900638 to	5786444 (2990868 to

© 2023 Chen N et al. *JAMA Network Open.*

				15251798)	9624969)
Albania	816	0.072	160	26407 (12977 to 45618)	4221 (2074 to 7292)
Andorra	3141	0.277	615	816 (411 to 1369)	502 (253 to 842)
Armenia	1387	0.122	272	26580 (14287 to 42959)	7222 (3882 to 11672)
Austria	6132	0.541	1201	67836 (34252 to 116615)	81486 (41145 to 140081)
Azerbaijan	550	0.048	108	75586 (38133 to 124274)	8144 (4108 to 13390)
Belarus	1163	0.103	228	95322 (51496 to 155727)	21717 (11732 to 35479)
Belgium	5698	0.502	1116	96614 (49230 to 164797)	107841 (54951 to 183947)
Bosnia and Herzegovina	1416	0.125	277	33223 (16940 to 56027)	9216 (4699 to 15541)
Bulgaria	1896	0.167	371	82408 (43028 to 135414)	30608 (15981 to 50295)
Croatia	2020	0.178	396	52741 (28282 to 86576)	20870 (11191 to 34258)
Cyprus	1881	0.166	368	9185 (4383 to 16379)	3384 (1615 to 6035)
Czechia	3296	0.291	646	133518 (69846 to 217396)	86208 (45097 to 140365)
Denmark	6112	0.539	1197	58431 (29638 to 99832)	69960 (35486 to 119529)
Estonia	2623	0.231	514	15375 (8753 to 24224)	7900 (4498 to 12447)
Finland	4680	0.413	917	47064 (24067 to 78909)	43147 (22064 to 72342)
France	5605	0.494	1098	575659 (300937 to 956819)	632066 (330424 to 1050573)
Georgia	1133	0.100	222	27213 (14479 to 45551)	6040 (3214 to 10110)
Germany	6482	0.571	1270	948506 (489442 to 1580790)	1204399 (621486 to 2007263)
Greece	2551	0.225	500	95371 (47244 to 164086)	47659 (23609 to 81998)
Hungary	2321	0.205	455	130491 (73149 to 206859)	59331 (33259 to 94052)
Iceland	5227	0.461	1024	3372 (1746 to 5581)	3452 (1788 to 5715)
Ireland	6362	0.561	1246	49265 (25120 to 82601)	61398 (31306 to 102944)
Israel	3056	0.269	599	70215 (34508 to 119585)	42034 (20658 to 71590)
Italy	3916	0.345	767	512845 (241981 to 909900)	393414 (185629 to 698003)
Kazakhstan	855	0.075	167	142810 (77266 to	23919 (12941 to

© 2023 Chen N et al. *JAMA Network Open.*

				226533)	37942)
Kyrgyzstan	350	0.031	69	30430 (15149 to 52143)	2086 (1039 to 3575)
Latvia	2008	0.177	393	23065 (12888 to 36736)	9073 (5069 to 14450)
Lithuania	2562	0.226	502	30947 (16009 to 51244)	15532 (8035 to 25718)
Luxembourg	6436	0.567	1261	5156 (2621 to 8851)	6500 (3305 to 11159)
Malta	4803	0.423	941	4266 (2076 to 7418)	4014 (1953 to 6979)
Monaco	3479	0.307	682	460 (233 to 775)	313 (159 to 528)
Montenegro	1840	0.162	360	7540 (4055 to 12174)	2718 (1461 to 4388)
Netherlands	6217	0.548	1218	134076 (64136 to 232387)	163288 (78110 to 283018)
North Macedonia	1137	0.100	223	21849 (11317 to 35976)	4867 (2521 to 8013)
Norway	7013	0.618	1374	36828 (18055 to 63866)	50595 (24804 to 87740)
Poland	2256	0.199	442	475528 (256587 to 768822)	210153 (113395 to 339771)
Portugal	3350	0.295	656	106018 (50928 to 184884)	69574 (33421 to 121329)
Republic of Moldova	667	0.059	131	36719 (19417 to 59303)	4798 (2537 to 7749)
Romania	1819	0.160	356	244640 (143898 to 384876)	87173 (51275 to 137143)
Russian Federation	1546	0.136	303	1713553 (953477 to 2718512)	518953 (288763 to 823307)
San Marino	4612	0.407	903	335 (171 to 558)	303 (154 to 504)
Serbia	1302	0.115	255	104108 (53589 to 168696)	26553 (13668 to 43026)
Slovakia	2373	0.209	465	61789 (32623 to 101050)	28723 (15165 to 46974)
Slovenia	3456	0.305	677	25251 (13476 to 40720)	17095 (9124 to 27568)
Spain	3985	0.351	781	360956 (185577 to 606277)	281776 (144868 to 473283)
Sweden	6149	0.542	1205	86434 (43704 to 145525)	104114 (52643 to 175292)
Switzerland	8521	0.751	1669	78780 (38484 to 137132)	131500 (64237 to 228902)
Tajikistan	251	0.022	49	22426 (9791 to 42474)	1103 (481 to 2088)
Turkey	1316	0.116	258	834448 (443730 to 1311407)	215118 (114392 to 338076)
Turkmenistan	1247	0.110	244	33653 (17855 to 54782)	8221 (4362 to 13382)
Ukraine	957	0.084	187	508052 (284467 to 808395)	95245 (53329 to 151550)

© 2023 Chen N et al. *JAMA Network Open.*

United Kingdom	4960	0.437	972	758847 (383385 to 1258988)	737322 (372510 to 1223276)
Uzbekistan	391	0.034	77	177530 (91344 to 293409)	13598 (6996 to 22474)
Southeast Asia	311	0.027	61	4736245 (2336292 to 8245158)	292618 (145155 to 506720)
Bangladesh	129	0.011	25	300385 (124528 to 577032)	7591 (3147 to 14582)
Bhutan	315	0.028	62	2669 (1196 to 4840)	165 (74 to 299)
India	253	0.022	50	2700625 (1350094 to 4666679)	133846 (66912 to 231286)
Indonesia	373	0.033	73	1059023 (544679 to 1804483)	77381 (39799 to 131851)
Maldives	2145	0.189	420	1561 (714 to 2846)	656 (300 to 1196)
Myanmar	233	0.021	46	140088 (62012 to 255328)	6394 (2830 to 11654)
Nepal	188	0.017	37	81679 (35161 to 154871)	3008 (1295 to 5704)
Sri Lanka	555	0.049	109	80396 (38874 to 134033)	8741 (4226 to 14572)
Thailand	759	0.067	149	368384 (178576 to 641880)	54773 (26551 to 95437)
Timor-Leste	226	0.020	44	1434 (459 to 3167)	63 (20 to 140)
Western Pacific	1670	0.147	327	5722976 (2229000 to 11210162)	1809101 (710155 to 3541044)
Australia	5398	0.476	1057	272644 (149934 to 429138)	288304 (158546 to 453786)
Brunei Darussalam	1454	0.128	285	1614 (647 to 3111)	460 (184 to 886)
Cambodia	233	0.021	46	32993 (13084 to 65565)	1506 (597 to 2993)
China	893	0.079	175	3684595 (1376321 to 7358638)	644558 (240764 to 1287271)
Cook Islands	1056	0.093	207	223 (125 to 343)	46 (26 to 71)
Fiji	498	0.044	98	7376 (4051 to 11464)	720 (395 to 1118)
Japan	4787	0.422	938	578771 (180581 to 1255913)	542740 (169339 to 1177725)
Kiribati	259	0.023	51	663 (318 to 1116)	34 (16 to 57)
Lao People's Democratic Republic	202	0.018	40	18189 (8068 to 32264)	720 (319 to 1277)
Malaysia	1200	0.106	235	160351 (86289 to 262295)	37694 (20284 to 61658)
Marshall Islands	499	0.044	98	268 (119 to 475)	26 (12 to 46)

© 2023 Chen N et al. *JAMA Network Open.*

Micronesia (Federated States of)	131	0.012	26	729 (385 to 1175)	19 (10 to 30)
Mongolia	623	0.055	122	15863 (7831 to 27586)	1936 (956 to 3367)
Nauru	1387	0.122	272	60 (30 to 99)	16 (8 to 27)
New Zealand	4434	0.391	869	45608 (24221 to 74804)	39615 (21038 to 64975)
Niue	849	0.075	166	18 (10 to 28)	3 (2 to 5)
Palau	1817	0.160	356	198 (105 to 315)	70 (37 to 112)
Papua New Guinea	106	0.009	21	27699 (10654 to 54919)	575 (221 to 1140)
Philippines	396	0.035	78	380214 (176610 to 652374)	29495 (13700 to 50607)
Republic of Korea	3529	0.311	691	254245 (92637 to 506965)	175762 (64041 to 350470)
Samoa	319	0.028	62	1583 (841 to 2508)	99 (53 to 157)
Singapore	4645	0.409	910	26051 (12515 to 45522)	23705 (11388 to 41421)
Solomon Islands	116	0.010	23	2637 (1162 to 4678)	60 (26 to 106)
Tonga	290	0.026	57	855 (480 to 1310)	49 (27 to 74)
Tuvalu	835	0.074	164	76 (37 to 126)	12 (6 to 21)
Vanuatu	92	0.008	18	1567 (743 to 2731)	28 (13 to 49)
Viet Nam	512	0.045	100	207885 (81201 to 414700)	20850 (8144 to 41594)

Abbreviation: $PPP, 2020 purchasing-power parity-adjusted US$.

Values in parentheses represent uncertainty levels derived from the sensitivity analyses.

© 2023 Chen N et al. *JAMA Network Open.*

eTable 10. Health-care costs of gout attributable to high body mass index by location in 2019

Location	Health-care costs per capita, 2019 ($PPP)	Spending ratio	Health-care costs per case, 2019 (US$)	Prevalence (uncertainty level)	Health-care costs (uncertainty level, US$, thousands)
Global	3073	0.271	103	16947252 (7454900 to 32457792)	2483397 (1225572 to 4288596)
Africa	318	0.028	11	758571 (329691 to 1485300)	10509 (5008 to 19214)
Algeria	765	0.067	26	82950 (40110 to 150719)	2133 (1031 to 3875)
Angola	195	0.017	7	12555 (4528 to 26958)	82 (30 to 177)
Benin	79	0.007	3	8758 (3934 to 16633)	23 (10 to 44)
Botswana	1144	0.101	38	3367 (1600 to 6249)	129 (62 to 240)
Burkina Faso	119	0.010	4	9313 (3365 to 20329)	37 (13 to 81)
Burundi	86	0.008	3	2925 (836 to 7306)	8 (2 to 21)
Cabo Verde	380	0.033	13	770 (360 to 1446)	10 (5 to 18)
Cameroon	121	0.011	4	27598 (12841 to 51004)	112 (52 to 207)
Central African Republic	61	0.005	2	1308 (375 to 3272)	3 (1 to 7)
Chad	71	0.006	2	4283 (1396 to 9971)	10 (3 to 24)
Comoros	187	0.016	6	576 (226 to 1197)	4 (1 to 8)
Congo	93	0.008	3	5055 (2258 to 9646)	16 (7 to 30)
Democratic Republic of the Congo	43	0.004	1	36104 (12918 to 78970)	52 (19 to 114)
Equatorial Guinea	554	0.049	19	1245 (603 to 2203)	23 (11 to 41)
Eritrea	46	0.004	2	2184 (782 to 4828)	3 (1 to 7)
Eswatini	593	0.052	20	1685 (847 to 2951)	34 (17 to 59)
Ethiopia	75	0.007	3	38867 (12745 to 88280)	98 (32 to 223)

© 2023 Chen N et al. *JAMA Network Open.*

Gabon	541	0.048	18	2441 (1130 to 4500)	44 (21 to 82)
Gambia	118	0.010	4	1519 (644 to 2972)	6 (3 to 12)
Ghana	189	0.017	6	31554 (14956 to 58008)	200 (95 to 368)
Guinea	124	0.011	4	5548 (2042 to 12047)	23 (9 to 50)
Guinea-Bissau	178	0.016	6	714 (258 to 1563)	4 (2 to 9)
Ivory Coast	170	0.015	6	18291 (7422 to 36733)	104 (42 to 210)
Kenya	248	0.022	8	39482 (17606 to 76540)	329 (147 to 638)
Lesotho	390	0.034	13	2279 (1078 to 4261)	30 (14 to 56)
Liberia	154	0.014	5	4244 (1923 to 7988)	22 (10 to 41)
Madagascar	70	0.006	2	10320 (3758 to 22605)	24 (9 to 53)
Malawi	107	0.009	4	8629 (3209 to 18102)	31 (12 to 65)
Mali	80	0.007	3	9239 (3448 to 19605)	25 (9 to 53)
Mauritania	210	0.019	7	4491 (2116 to 8326)	32 (15 to 59)
Mauritius	1553	0.137	52	4066 (1736 to 7773)	212 (91 to 406)
Mozambique	100	0.009	3	10793 (3981 to 23335)	36 (13 to 78)
Namibia	964	0.085	32	2666 (1280 to 4897)	86 (41 to 159)
Niger	71	0.006	2	6152 (2110 to 14239)	15 (5 to 34)
Nigeria	192	0.017	6	127274 (55380 to 249798)	821 (357 to 1612)
Rwanda	153	0.013	5	5504 (1879 to 12372)	28 (10 to 64)
Sao Tome and Principe	234	0.021	8	211 (95 to 402)	2 (1 to 3)
Senegal	158	0.014	5	10065 (4193 to 20271)	53 (22 to 108)
Seychelles	1440	0.127	48	284 (124 to 543)	14 (6 to 26)
Sierra Leone	241	0.021	8	3535 (1189 to 7940)	29 (10 to 64)

© 2023 Chen N et al. *JAMA Network Open.*

South Africa	1202	0.106	40	128802 (64882 to 224391)	5203 (2621 to 9064)
South Sudan	83	0.007	3	5464 (2230 to 11037)	15 (6 to 31)
Togo	96	0.008	3	4385 (1764 to 8992)	14 (6 to 29)
Uganda	131	0.012	4	16354 (6163 to 34339)	72 (27 to 151)
United Republic of Tanzania	103	0.009	3	36105 (15629 to 72083)	125 (54 to 250)
Zambia	205	0.018	7	9949 (4086 to 19882)	69 (28 to 137)
Zimbabwe	211	0.019	7	8667 (3657 to 17790)	61 (26 to 126)
The Americas	7659	0.675	257	5628341 (2897828 to 9321606)	1793366 (939344 to 2912800)
Antigua and Barbuda	1281	0.113	43	96 (44 to 175)	4 (2 to 8)
Argentina	2285	0.201	77	177989 (77740 to 335389)	13668 (5970 to 25754)
Bahamas	2468	0.218	83	449 (224 to 804)	37 (19 to 67)
Barbados	1020	0.090	34	471 (231 to 845)	16 (8 to 29)
Belize	431	0.038	14	382 (189 to 675)	6 (3 to 10)
Bolivia (Plurinational State of)	585	0.052	20	10619 (4981 to 19298)	209 (98 to 379)
Brazil	1443	0.127	48	261936 (130106 to 462127)	12702 (6309 to 22410)
Canada	5837	0.514	196	399558 (184057 to 733296)	78376 (36104 to 143841)
Chile	2472	0.218	83	94656 (42533 to 174790)	7863 (3533 to 14520)
Colombia	1214	0.107	41	33368 (15753 to 60810)	1361 (643 to 2481)
Costa Rica	1798	0.158	60	3763 (1795 to 6808)	227 (108 to 411)
Cuba	2863	0.252	96	16981 (8288 to 30387)	1634 (797 to 2924)
Dominica	676	0.060	23	92 (45 to 165)	2 (1 to 4)
Dominican Republic	1124	0.099	38	9166 (4230 to 16946)	346 (160 to 640)
Ecuador	963	0.085	32	24883 (12775 to 42883)	805 (413 to 1388)

© 2023 Chen N et al. *JAMA Network Open.*

El Salvador	707	0.062	24	3922 (1871 to 7076)	93 (44 to 168)
Grenada	863	0.076	29	103 (49 to 190)	3 (1 to 6)
Guatemala	601	0.053	20	6081 (2516 to 12016)	123 (51 to 243)
Guyana	648	0.057	22	587 (271 to 1087)	13 (6 to 24)
Haiti	111	0.010	4	3033 (1073 to 6760)	11 (4 to 25)
Honduras	429	0.038	14	3722 (1597 to 7201)	54 (23 to 104)
Jamaica	715	0.063	24	3185 (1611 to 5637)	77 (39 to 135)
Mexico	1171	0.103	39	107705 (52610 to 191065)	4238 (2070 to 7519)
Nicaragua	501	0.044	17	3195 (1455 to 5945)	54 (24 to 100)
Panama	2353	0.207	79	2717 (1273 to 5054)	215 (101 to 400)
Paraguay	968	0.085	33	5656 (2653 to 10424)	184 (86 to 339)
Peru	683	0.060	23	37532 (17207 to 68692)	861 (395 to 1577)
Saint Kitts and Nevis	1415	0.125	48	76 (37 to 135)	4 (2 to 6)
Saint Lucia	744	0.066	25	211 (101 to 381)	5 (3 to 10)
Saint Vincent and the Grenadines	626	0.055	21	128 (59 to 238)	3 (1 to 5)
Suriname	1420	0.125	48	600 (285 to 1104)	29 (14 to 53)
Trinidad and Tobago	1836	0.162	62	2079 (1046 to 3665)	128 (65 to 226)
United States of America	11345	1.000	381	4376671 (2312452 to 7040919)	1668639 (881640 to 2684403)
Uruguay	2156	0.190	72	16871 (7401 to 31863)	1222 (536 to 2309)
Venezuela (Bolivarian Republic of)	230	0.020	8	19858 (9268 to 36757)	153 (72 to 284)
Eastern Mediterranean	832	0.073	28	926358 (431363 to 1694518)	32130 (15603 to 56576)
Afghanistan	223	0.020	7	19898 (8602 to 38936)	149 (64 to 292)
Bahrain	2260	0.199	76	4765 (2293 to 8306)	362 (174 to 631)

© 2023 Chen N et al. *JAMA Network Open.*

Djibouti	88	0.008	3	807 (286 to 1730)	2 (1 to 5)
Egypt	617	0.054	21	182500 (88608 to 322139)	3784 (1837 to 6679)
Iran (Islamic Republic of)	1112	0.098	37	160062 (75038 to 290751)	5981 (2804 to 10865)
Iraq	415	0.037	14	62227 (29766 to 110485)	868 (415 to 1541)
Jordan	734	0.065	25	23074 (11697 to 38596)	569 (289 to 952)
Kuwait	2724	0.240	92	12604 (6459 to 21108)	1154 (591 to 1932)
Lebanon	1663	0.147	56	12596 (6259 to 22215)	704 (350 to 1241)
Libya	1155	0.102	39	15446 (7638 to 26678)	600 (296 to 1035)
Morocco	429	0.038	14	64073 (29951 to 119413)	924 (432 to 1722)
Oman	1272	0.112	43	8689 (4269 to 15038)	371 (182 to 643)
Pakistan	158	0.014	5	122099 (47568 to 254741)	648 (253 to 1353)
Qatar	3017	0.266	101	8625 (4235 to 14425)	874 (429 to 1462)
Saudi Arabia	3223	0.284	108	87163 (43954 to 149223)	9441 (4761 to 16163)
Sudan	252	0.022	8	39502 (17567 to 73760)	335 (149 to 625)
Syrian Arab Republic	1046	0.092	35	27273 (12656 to 50313)	959 (445 to 1769)
Tunisia	884	0.078	30	26927 (12570 to 49146)	800 (373 to 1460)
United Arab Emirates	3203	0.282	108	32997 (16130 to 56646)	3552 (1736 to 6097)
Yemen	105	0.009	4	15030 (5818 to 30868)	53 (21 to 109)
Europe	3443	0.303	116	3077117 (1405687 to 5731746)	383483 (169681 to 727365)
Albania	816	0.072	27	4927 (2269 to 9286)	135 (62 to 255)
Andorra	3141	0.277	106	367 (158 to 698)	39 (17 to 74)
Armenia	1387	0.122	47	7284 (3585 to 12991)	339 (167 to 606)
Austria	6132	0.541	206	36031 (15443 to 70337)	7425 (3182 to 14494)

© 2023 Chen N et al. *JAMA Network Open.*

Azerbaijan	550	0.048	18	18403 (8674 to 33880)	340 (160 to 626)
Belarus	1163	0.103	39	25293 (12584 to 45573)	989 (492 to 1781)
Belgium	5698	0.502	191	43424 (18401 to 85261)	8315 (3524 to 16326)
Bosnia and Herzegovina	1416	0.125	48	6990 (3281 to 12984)	333 (156 to 618)
Bulgaria	1896	0.167	64	17865 (8513 to 32754)	1138 (542 to 2087)
Croatia	2020	0.178	68	11226 (5416 to 20239)	762 (368 to 1374)
Cyprus	1881	0.166	63	3601 (1463 to 7293)	228 (92 to 461)
Czechia	3296	0.291	111	28703 (14294 to 50338)	3179 (1583 to 5576)
Denmark	6112	0.539	205	21661 (9037 to 42528)	4449 (1856 to 8735)
Estonia	2623	0.231	88	4633 (2393 to 8222)	408 (211 to 725)
Finland	4680	0.413	157	23920 (10351 to 46809)	3762 (1628 to 7362)
France	5605	0.494	188	247495 (104995 to 484687)	46618 (19777 to 91296)
Georgia	1133	0.100	38	9180 (4304 to 16891)	350 (164 to 643)
Germany	6482	0.571	218	409168 (177230 to 778329)	89130 (38606 to 169545)
Greece	2551	0.225	86	49340 (20797 to 94899)	4230 (1783 to 8135)
Hungary	2321	0.205	78	26438 (13365 to 46588)	2062 (1042 to 3634)
Iceland	5227	0.461	176	1370 (608 to 2597)	241 (107 to 456)
Ireland	6362	0.561	214	17690 (7795 to 33705)	3782 (1667 to 7206)
Israel	3056	0.269	103	25176 (10909 to 48218)	2586 (1120 to 4952)
Italy	3916	0.345	132	223152 (93731 to 439277)	29367 (12335 to 57809)
Kazakhstan	855	0.075	29	36378 (17898 to 64412)	1045 (514 to 1851)
Kyrgyzstan	350	0.031	12	7068 (3192 to 13406)	83 (38 to 158)

© 2023 Chen N et al. *JAMA Network Open.*

Latvia	2008	0.177	67	6529 (3216 to 11585)	441 (217 to 782)
Lithuania	2562	0.226	86	8809 (4235 to 16066)	758 (365 to 1383)
Luxembourg	6436	0.567	216	2258 (987 to 4375)	488 (214 to 946)
Malta	4803	0.423	161	1742 (707 to 3488)	281 (114 to 563)
Monaco	3479	0.307	117	220 (100 to 414)	26 (12 to 48)
Montenegro	1840	0.162	62	1428 (697 to 2530)	88 (43 to 156)
Netherlands	6217	0.548	209	67225 (27424 to 135185)	14045 (5730 to 28244)
North Macedonia	1137	0.100	38	4528 (2193 to 8339)	173 (84 to 319)
Norway	7013	0.618	236	15609 (6386 to 31103)	3679 (1505 to 7330)
Poland	2256	0.199	76	99947 (49731 to 177335)	7577 (3770 to 13445)
Portugal	3350	0.295	113	43734 (17875 to 87242)	4923 (2012 to 9822)
Republic of Moldova	667	0.059	22	9544 (4635 to 17471)	214 (104 to 392)
Romania	1819	0.160	61	50337 (25851 to 88618)	3077 (1580 to 5417)
Russian Federation	1546	0.136	52	426926 (212011 to 759289)	22181 (11015 to 39448)
San Marino	4612	0.407	155	146 (64 to 275)	23 (10 to 43)
Serbia	1302	0.115	44	21706 (10308 to 39566)	950 (451 to 1731)
Slovakia	2373	0.209	80	12587 (6199 to 22770)	1004 (494 to 1816)
Slovenia	3456	0.305	116	5816 (2866 to 10262)	675 (333 to 1192)
Spain	3985	0.351	134	210709 (92977 to 396450)	28218 (12451 to 53092)
Sweden	6149	0.542	207	38299 (16870 to 74026)	7914 (3486 to 15297)
Switzerland	8521	0.751	286	31294 (12633 to 62056)	8961 (3618 to 17770)
Tajikistan	251	0.022	8	4909 (1791 to 10687)	41 (15 to 90)
Turkey	1316	0.116	44	223524 (110106 to 387268)	9885 (4869 to 17127)
Turkmenistan	1247	0.110	42	8302 (4053 to 14814)	348 (170 to 621)

© 2023 Chen N et al. *JAMA Network Open.*

Ukraine	957	0.084	32	124081 (61204 to 222142)	3991 (1968 to 7144)
United Kingdom	4960	0.437	167	309918 (141692 to 572290)	51659 (23618 to 95392)
Uzbekistan	391	0.034	13	40211 (18190 to 73900)	528 (239 to 971)
Southeast Asia	311	0.027	10	1820023 (741420 to 3736221)	20569 (8413 to 42068)
Bangladesh	129	0.011	4	81424 (27858 to 185193)	353 (121 to 803)
Bhutan	315	0.028	11	620 (238 to 1290)	7 (3 to 14)
India	253	0.022	9	1034895 (429656 to 2110525)	8799 (3653 to 17944)
Indonesia	373	0.033	13	386035 (158052 to 779003)	4839 (1981 to 9765)
Maldives	2145	0.189	72	719 (270 to 1474)	52 (19 to 106)
Myanmar	233	0.021	8	52811 (18961 to 114900)	414 (148 to 900)
Nepal	188	0.017	6	17244 (6148 to 37924)	109 (39 to 240)
Sri Lanka	555	0.049	19	40013 (15462 to 84945)	746 (288 to 1584)
Thailand	759	0.067	26	205725 (84636 to 419573)	5247 (2159 to 10702)
Timor-Leste	226	0.020	8	536 (139 to 1394)	4 (1 to 11)
Western Pacific	1670	0.147	56	4736841 (1648912 to 10488401)	243339 (87522 to 530573)
Australia	5398	0.476	181	257382 (124285 to 468179)	46690 (22546 to 84930)
Brunei Darussalam	1454	0.128	49	692 (240 to 1485)	34 (12 to 73)
Cambodia	233	0.021	8	11184 (3695 to 25981)	88 (29 to 203)
China	893	0.079	30	3598725 (1223341 to 8050029)	107998 (36712 to 241581)
Cook Islands	1056	0.093	35	109 (55 to 187)	4 (2 to 7)
Fiji	498	0.044	17	3137 (1513 to 5530)	52 (25 to 93)
Japan	4787	0.422	161	346571 (97348 to 843427)	55753 (15660 to 135683)
Kiribati	259	0.023	9	199 (84 to 390)	2 (1 to 3)

© 2023 Chen N et al. *JAMA Network Open.*

Lao People's Democratic Republic	202	0.018	7	6927 (2580 to 14482)	47 (18 to 98)
Malaysia	1200	0.106	40	74462 (33395 to 140768)	3003 (1347 to 5677)
Marshall Islands	499	0.044	17	93 (37 to 191)	2 (1 to 3)
Micronesia (Federated States of)	131	0.012	4	243 (114 to 456)	1 (1 to 2)
Mongolia	623	0.055	21	3802 (1659 to 7205)	80 (35 to 151)
Nauru	1387	0.122	47	17 (8 to 33)	0.812 (0.369 to 1.516)
New Zealand	4434	0.391	149	46629 (22874 to 80584)	6948 (3408 to 12008)
Niue	849	0.075	29	8 (4 to 15)	0.239 (0.116 to 0.431)
Palau	1817	0.160	61	96 (45 to 172)	6 (3 to 10)
Papua New Guinea	106	0.009	4	7867 (2507 to 18326)	28 (9 to 65)
Philippines	396	0.035	13	132608 (53053 to 272710)	1765 (706 to 3629)
Republic of Korea	3529	0.311	119	138872 (45212 to 311627)	16469 (5362 to 36957)
Samoa	319	0.028	11	576 (266 to 1039)	6 (3 to 11)
Singapore	4645	0.409	156	18338 (7354 to 37428)	2863 (1148 to 5842)
Solomon Islands	116	0.010	4	768 (290 to 1582)	3 (1 to 6)
Tonga	290	0.026	10	300 (150 to 529)	3 (1 to 5)
Tuvalu	835	0.074	28	29 (12 to 57)	0.814 (0.346 to 1.588)
Vanuatu	92	0.008	3	455 (190 to 916)	1 (1 to 3)
Viet Nam	512	0.045	17	86752 (28601 to 205074)	1493 (492 to 3529)

Abbreviation: $PPP, 2020 purchasing-power parity-adjusted US$.
Values in parentheses represent uncertainty levels derived from the sensitivity analyses.

© 2023 Chen N et al. *JAMA Network Open.*

eTable 11. Health-care costs of osteoarthritis attributable to high body mass index by location in 2019

Location	Health-care costs per capita, 2019 ($PPP)	Spending ratio	Health-care costs per case, 2019 (US$)	Prevalence (uncertainty level)	Health-care costs (uncertainty level, US$, thousands)
Global	3073	0.271	522	72969260 (32371084 to 131052677)	34888834 (16685541 to 59792908)
Africa	318	0.028	54	3964819 (1814204 to 7032158)	272887 (136781 to 451337)
Algeria	765	0.067	130	443930 (218134 to 717656)	57657 (28331 to 93209)
Angola	195	0.017	33	63278 (24502 to 122093)	2095 (811 to 4042)
Benin	79	0.007	13	48948 (22767 to 86221)	657 (305 to 1156)
Botswana	1144	0.101	194	17822 (9137 to 29276)	3461 (1775 to 5686)
Burkina Faso	119	0.010	20	63811 (25138 to 123214)	1289 (508 to 2489)
Burundi	86	0.008	15	14323 (4883 to 30864)	209 (71 to 451)
Cabo Verde	380	0.033	65	4521 (2198 to 7743)	292 (142 to 500)
Cameroon	121	0.011	21	148843 (73404 to 248944)	3058 (1508 to 5114)
Central African Republic	61	0.005	10	6991 (2199 to 15258)	72 (23 to 158)
Chad	71	0.006	12	24180 (8797 to 48457)	291 (106 to 584)
Comoros	187	0.016	32	2944 (1207 to 5590)	93 (38 to 177)
Congo	93	0.008	16	24918 (11709 to 43018)	393 (185 to 679)
Democratic Republic of the Congo	43	0.004	7	162531 (63546 to 326723)	1187 (464 to 2385)
Equatorial Guinea	554	0.049	94	5765 (2916 to 9782)	542 (274 to 920)

© 2023 Chen N et al. *JAMA Network Open.*

Eritrea	46	0.004	8	11955 (4411 to 23361)	93 (34 to 182)
Eswatini	593	0.052	101	8708 (4480 to 13944)	877 (451 to 1404)
Ethiopia	75	0.007	13	162527 (59512 to 333345)	2070 (758 to 4245)
Gabon	541	0.048	92	12772 (6252 to 21215)	1173 (574 to 1949)
Gambia	118	0.010	20	8098 (3641 to 14465)	162 (73 to 290)
Ghana	189	0.017	32	190601 (94902 to 322196)	6116 (3045 to 10339)
Guinea	124	0.011	21	33685 (13743 to 63940)	709 (289 to 1346)
Guinea-Bissau	178	0.016	30	4285 (1667 to 8306)	129 (50 to 251)
Ivory Coast	170	0.015	29	92763 (41276 to 166678)	2677 (1191 to 4811)
Kenya	248	0.022	42	170821 (78224 to 304356)	7192 (3294 to 12815)
Lesotho	390	0.034	66	13601 (6754 to 23181)	901 (447 to 1535)
Liberia	154	0.014	26	22275 (10649 to 38134)	582 (278 to 997)
Madagascar	70	0.006	12	52061 (20113 to 102112)	619 (239 to 1214)
Malawi	107	0.009	18	43439 (17058 to 84309)	789 (310 to 1532)
Mali	80	0.007	14	53348 (21510 to 100459)	725 (292 to 1364)
Mauritania	210	0.019	36	24468 (12318 to 40905)	872 (439 to 1458)
Mauritius	1553	0.137	264	15527 (6929 to 26890)	4094 (1827 to 7090)
Mozambique	100	0.009	17	55184 (21752 to 107812)	937 (369 to 1830)
Namibia	964	0.085	164	13988 (7100 to 23863)	2289 (1162 to 3906)
Niger	71	0.006	12	37893 (14328 to 75804)	457 (173 to 914)
Nigeria	192	0.017	33	715885 (314830 to 1308373)	23336 (10263 to 42649)
Rwanda	153	0.013	26	33406 (12445 to 66513)	868 (323 to 1728)

© 2023 Chen N et al. *JAMA Network Open.*

Sao Tome and Principe	234	0.021	40	1143 (550 to 1981)	45 (22 to 79)
Senegal	158	0.014	27	59527 (26370 to 105690)	1597 (707 to 2835)
Seychelles	1440	0.127	244	1034 (470 to 1789)	253 (115 to 437)
Sierra Leone	241	0.021	41	21045 (8069 to 41078)	861 (330 to 1681)
South Africa	1202	0.106	204	644839 (347993 to 1016254)	131594 (71016 to 207389)
South Sudan	83	0.007	14	27600 (12146 to 50506)	389 (171 to 712)
Togo	96	0.008	16	28377 (12217 to 52658)	463 (199 to 858)
Uganda	131	0.012	22	84728 (34822 to 162317)	1884 (774 to 3610)
United Republic of Tanzania	103	0.009	17	184086 (82431 to 328140)	3219 (1441 to 5738)
Zambia	205	0.018	35	47954 (20900 to 88044)	1669 (727 to 3064)
Zimbabwe	211	0.019	36	54391 (23806 to 98702)	1948 (853 to 3536)
The Americas	7659	0.675	1300	17395805 (8698214 to 28824471)	18250463 (9302403 to 29919670)
Antigua and Barbuda	1281	0.113	217	1273 (607 to 2168)	277 (132 to 471)
Argentina	2285	0.201	388	625917 (291074 to 1093626)	242819 (112919 to 424262)
Bahamas	2468	0.218	419	5926 (2919 to 9802)	2483 (1223 to 4107)
Barbados	1020	0.090	173	7000 (3388 to 11560)	1212 (587 to 2002)
Belize	431	0.038	73	4419 (2173 to 7147)	323 (159 to 523)
Bolivia (Plurinational State of)	585	0.052	99	121092 (59593 to 198515)	12027 (5919 to 19716)
Brazil	1443	0.127	245	3205837 (1642826 to 5289373)	785393 (402473 to 1295835)
Canada	5837	0.514	991	550545 (254215 to 969187)	545584 (251925 to 960453)

© 2023 Chen N et al. *JAMA Network Open.*

Chile	2472	0.218	420	342119 (164185 to 582151)	143583 (68907 to 244322)
Colombia	1214	0.107	206	637591 (300616 to 1079644)	131413 (61960 to 222525)
Costa Rica	1798	0.158	305	67756 (31557 to 115232)	20683 (9633 to 35176)
Cuba	2863	0.252	486	246645 (119192 to 411772)	119887 (57936 to 200151)
Dominica	676	0.060	115	1277 (633 to 2077)	147 (73 to 238)
Dominican Republic	1124	0.099	191	109592 (49325 to 188335)	20913 (9413 to 35940)
Ecuador	963	0.085	163	253249 (133982 to 389205)	41405 (21905 to 63633)
El Salvador	707	0.062	120	75919 (34335 to 129583)	9113 (4121 to 15554)
Grenada	863	0.076	147	1411 (685 to 2378)	207 (100 to 348)
Guatemala	601	0.053	102	103589 (44937 to 186608)	10570 (4585 to 19041)
Guyana	648	0.057	110	7705 (3561 to 13248)	848 (392 to 1457)
Haiti	111	0.010	19	34940 (12578 to 71493)	658 (237 to 1347)
Honduras	429	0.038	73	64369 (28054 to 114204)	4688 (2043 to 8318)
Jamaica	715	0.063	121	41629 (21121 to 67883)	5053 (2564 to 8240)
Mexico	1171	0.103	199	1681697 (818145 to 2809029)	334337 (162655 to 558460)
Nicaragua	501	0.044	85	54211 (24729 to 93545)	4611 (2103 to 7957)
Panama	2353	0.207	399	48615 (22822 to 82531)	19421 (9117 to 32970)
Paraguay	968	0.085	164	67365 (32489 to 114007)	11071 (5339 to 18736)
Peru	683	0.060	116	420532 (195319 to 707296)	48764 (22649 to 82017)
Saint Kitts and Nevis	1415	0.125	240	985 (483 to 1637)	237 (116 to 393)
Saint Lucia	744	0.066	126	2903 (1386 to 4811)	367 (175 to 608)
Saint Vincent and the Grenadines	626	0.055	106	1708 (802 to 2869)	182 (85 to 305)

© 2023 Chen N et al. *JAMA Network Open.*

Suriname	1420	0.125	241	7981 (3698 to 13342)	1924 (892 to 3217)
Trinidad and Tobago	1836	0.162	312	28755 (14625 to 46427)	8963 (4559 to 14472)
United States of America	11345	1.000	1926	8142942 (4183684 to 13276933)	15684295 (8058284 to 25572985)
Uruguay	2156	0.190	366	62023 (28970 to 107391)	22703 (10604 to 39309)
Venezuela (Bolivarian Republic of)	230	0.020	39	366290 (169507 to 629464)	14303 (6619 to 24580)
Eastern Mediterranean	832	0.073	141	4618052 (2266815 to 7707889)	736293 (378115 to 1190971)
Afghanistan	223	0.020	38	102633 (47629 to 179971)	3886 (1803 to 6814)
Bahrain	2260	0.199	384	19623 (10073 to 31019)	7529 (3865 to 11902)
Djibouti	88	0.008	15	3538 (1413 to 6838)	53 (21 to 102)
Egypt	617	0.054	105	956026 (495363 to 1510445)	100146 (51890 to 158223)
Iran (Islamic Republic of)	1112	0.098	189	794664 (398822 to 1329914)	150026 (75294 to 251077)
Iraq	415	0.037	70	303851 (146488 to 493879)	21409 (10321 to 34797)
Jordan	734	0.065	125	114445 (60647 to 178119)	14262 (7558 to 22197)
Kuwait	2724	0.240	462	55789 (30529 to 85040)	25801 (14119 to 39329)
Lebanon	1663	0.147	282	67725 (34029 to 108183)	19121 (9608 to 30544)
Libya	1155	0.102	196	78567 (41058 to 125083)	15406 (8051 to 24528)
Morocco	429	0.038	73	365284 (175239 to 613377)	26605 (12763 to 44675)
Oman	1272	0.112	216	30966 (16271 to 49159)	6687 (3514 to 10616)
Pakistan	158	0.014	27	651312 (270421 to 1239388)	17471 (7254 to 33246)
Qatar	3017	0.266	512	26481 (14145 to 40885)	13564 (7245 to 20942)

© 2023 Chen N et al. *JAMA Network Open.*

Saudi Arabia	3223	0.284	547	358678 (190902 to 571282)	196266 (104460 to 312600)
Sudan	252	0.022	43	194836 (93113 to 329586)	8336 (3984 to 14101)
Syrian Arab Republic	1046	0.092	178	156930 (76143 to 256009)	27869 (13522 to 45464)
Tunisia	884	0.078	150	155210 (75974 to 252717)	23294 (11402 to 37929)
United Arab Emirates	3203	0.282	544	105188 (56774 to 164291)	57201 (30873 to 89341)
Yemen	105	0.009	18	76307 (31784 to 142701)	1360 (567 to 2544)
Europe	3443	0.303	585	17248229 (8339493 to 29272616)	10146041 (4863395 to 17347704)
Albania	816	0.072	139	40066 (19475 to 66975)	5551 (2698 to 9279)
Andorra	3141	0.277	533	1575 (720 to 2788)	840 (384 to 1487)
Armenia	1387	0.122	235	38918 (19489 to 65123)	9164 (4589 to 15335)
Austria	6132	0.541	1041	171285 (80272 to 302307)	178320 (83569 to 314724)
Azerbaijan	550	0.048	93	94139 (46068 to 160613)	8790 (4302 to 14998)
Belarus	1163	0.103	197	175154 (87246 to 291913)	34584 (17227 to 57638)
Belgium	5698	0.502	967	208002 (97220 to 366698)	201219 (94050 to 354740)
Bosnia and Herzegovina	1416	0.125	240	58493 (29023 to 97733)	14062 (6977 to 23495)
Bulgaria	1896	0.167	322	146158 (73580 to 238769)	47048 (23685 to 76859)
Croatia	2020	0.178	343	91670 (46213 to 147351)	31438 (15849 to 50534)
Cyprus	1881	0.166	319	16462 (7137 to 29885)	5257 (2279 to 9544)
Czechia	3296	0.291	560	233603 (120114 to 371208)	130721 (67214 to 207722)
Denmark	6112	0.539	1038	99693 (46516 to 175757)	103449 (48269 to 182379)
Estonia	2623	0.231	445	29930 (15576 to 48657)	13328 (6937 to 21668)

© 2023 Chen N et al. *JAMA Network Open.*

Finland	4680	0.413	795	118767 (56321 to 203692)	94367 (44750 to 161845)
France	5605	0.494	952	1246588 (593345 to 2141999)	1186254 (564627 to 2038328)
Georgia	1133	0.100	192	48457 (23439 to 84226)	9321 (4509 to 16201)
Germany	6482	0.571	1100	2005598 (991329 to 3354211)	2207151 (1090953 to 3691293)
Greece	2551	0.225	433	233815 (110079 to 394846)	101266 (47675 to 171008)
Hungary	2321	0.205	394	217750 (113733 to 348403)	85805 (44817 to 137289)
Iceland	5227	0.461	887	6003 (2744 to 10553)	5327 (2435 to 9365)
Ireland	6362	0.561	1080	82380 (39729 to 140403)	88980 (42913 to 151652)
Israel	3056	0.269	519	121586 (56332 to 213917)	63083 (29227 to 110988)
Italy	3916	0.345	665	1221241 (565514 to 2156283)	811939 (375980 to 1433599)
Kazakhstan	855	0.075	145	189759 (95091 to 316181)	27545 (13803 to 45897)
Kyrgyzstan	350	0.031	59	35263 (16268 to 62264)	2095 (967 to 3700)
Latvia	2008	0.177	341	44578 (22494 to 73878)	15197 (7668 to 25186)
Lithuania	2562	0.226	435	62022 (30491 to 101928)	26978 (13263 to 44336)
Luxembourg	6436	0.567	1093	10125 (4806 to 17412)	11064 (5251 to 19026)
Malta	4803	0.423	815	8366 (3880 to 14717)	6822 (3164 to 12001)
Monaco	3479	0.307	591	1087 (522 to 1823)	642 (308 to 1077)
Montenegro	1840	0.162	312	11517 (5905 to 18387)	3598 (1845 to 5744)
Netherlands	6217	0.548	1056	332864 (152982 to 579915)	351340 (161473 to 612103)
North Macedonia	1137	0.100	193	34862 (17668 to 57037)	6730 (3410 to 11010)
Norway	7013	0.618	1191	78580 (35357 to 140709)	93561 (42097 to 167534)

© 2023 Chen N et al. *JAMA Network Open.*

Poland	2256	0.199	383	784622 (408542 to 1249606)	300524 (156478 to 478621)
Portugal	3350	0.295	569	215362 (97405 to 385742)	122488 (55400 to 219392)
Republic of Moldova	667	0.059	113	65915 (32956 to 109651)	7464 (3732 to 12417)
Romania	1819	0.160	309	397953 (210842 to 632656)	122898 (65113 to 195380)
Russian Federation	1546	0.136	262	2646151 (1259737 to 4565082)	694550 (330650 to 1198222)
San Marino	4612	0.407	783	692 (333 to 1181)	542 (261 to 924)
Serbia	1302	0.115	221	174464 (86486 to 279905)	38565 (19118 to 61873)
Slovakia	2373	0.209	403	104184 (53673 to 167990)	41974 (21624 to 67680)
Slovenia	3456	0.305	587	45647 (23435 to 73039)	26783 (13750 to 42856)
Spain	3985	0.351	677	1012743 (423841 to 1934282)	685184 (286755 to 1308661)
Sweden	6149	0.542	1044	168251 (77567 to 291581)	175647 (80976 to 304398)
Switzerland	8521	0.751	1447	148527 (67930 to 261127)	214870 (98272 to 377765)
Tajikistan	251	0.022	43	23506 (9382 to 46122)	1002 (400 to 1965)
Turkey	1316	0.116	223	1360677 (683969 to 2168981)	304012 (152817 to 484608)
Turkmenistan	1247	0.110	212	39790 (20218 to 65955)	8424 (4280 to 13963)
Ukraine	957	0.084	162	851511 (435021 to 1395905)	138351 (70681 to 226802)
United Kingdom	4960	0.437	842	1505220 (734024 to 2518609)	1267537 (618118 to 2120907)
Uzbekistan	391	0.034	66	186657 (87458 to 326641)	12391 (5806 to 21683)
Southeast Asia	311	0.027	53	10030775 (4241270 to 18468603)	524816 (223215 to 961463)
Bangladesh	129	0.011	22	493413 (179142 to 1010516)	10806 (3923 to 22132)
Bhutan	315	0.028	53	4301 (1767 to 7910)	230 (95 to 423)

© 2023 Chen N et al. *JAMA Network Open.*

India	253	0.022	43	6893718 (2945986 to 12599482)	296110 (126541 to 541193)
Indonesia	373	0.033	63	1308679 (562269 to 2380201)	82875 (35607 to 150731)
Maldives	2145	0.189	364	1916 (786 to 3645)	698 (286 to 1327)
Myanmar	233	0.021	40	226252 (87824 to 442249)	8950 (3474 to 17495)
Nepal	188	0.017	32	116071 (42750 to 229407)	3705 (1365 to 7322)
Sri Lanka	555	0.049	94	159116 (64945 to 292173)	14993 (6119 to 27530)
Thailand	759	0.067	129	825560 (355314 to 1498931)	106382 (45786 to 193154)
Timor-Leste	226	0.020	38	1748 (485 to 4089)	67 (19 to 157)
Western Pacific	1670	0.147	284	19711581 (7011087 to 39746939)	4958333 (1781632 to 9921762)
Australia	5398	0.476	916	601563 (304723 to 983752)	551307 (279266 to 901567)
Brunei Darussalam	1454	0.128	247	2984 (1158 to 5826)	737 (286 to 1438)
Cambodia	233	0.021	40	42178 (14851 to 88424)	1668 (587 to 3498)
China	893	0.079	152	15385544 (5402464 to 31214066)	2332617 (819073 to 4732395)
Cook Islands	1056	0.093	179	425 (222 to 672)	76 (40 to 120)
Fiji	498	0.044	85	11887 (5854 to 19396)	1005 (495 to 1640)
Japan	4787	0.422	813	1699962 (514939 to 3619178)	1381599 (418503 to 2941389)
Kiribati	259	0.023	44	792 (340 to 1427)	35 (15 to 63)
Lao People's Democratic Republic	202	0.018	34	22450 (8936 to 43159)	770 (306 to 1480)
Malaysia	1200	0.106	204	235040 (111867 to 403398)	47885 (22791 to 82185)
Marshall Islands	499	0.044	85	344 (139 to 646)	29 (12 to 55)

© 2023 Chen N et al. *JAMA Network Open.*

Micronesia (Federated States of)	131	0.012	22	991 (473 to 1677)	22 (11 to 37)
Mongolia	623	0.055	106	17113 (7742 to 30672)	1810 (819 to 3244)
Nauru	1387	0.122	235	63 (29 to 109)	15 (7 to 26)
New Zealand	4434	0.391	753	98309 (48551 to 164521)	74006 (36549 to 123850)
Niue	849	0.075	144	33 (16 to 54)	5 (2 to 8)
Palau	1817	0.160	308	360 (177 to 591)	111 (55 to 182)
Papua New Guinea	106	0.009	18	29527 (10197 to 60721)	531 (184 to 1093)
Philippines	396	0.035	67	407623 (171506 to 756769)	27405 (11531 to 50879)
Republic of Korea	3529	0.311	599	742598 (255799 to 1503425)	444923 (153260 to 900768)
Samoa	319	0.028	54	2089 (1007 to 3505)	113 (55 to 190)
Singapore	4645	0.409	789	80376 (35471 to 146568)	63386 (27973 to 115586)
Solomon Islands	116	0.010	20	2809 (1119 to 5297)	55 (22 to 104)
Tonga	290	0.026	49	1198 (599 to 1935)	59 (29 to 95)
Tuvalu	835	0.074	142	110 (47 to 197)	16 (7 to 28)
Vanuatu	92	0.008	16	1702 (744 to 3073)	27 (12 to 48)
Viet Nam	512	0.045	87	323510 (112118 to 687884)	28121 (9746 to 59795)

Abbreviation: $PPP, 2020 purchasing-power parity-adjusted US$. Values in parentheses represent uncertainty levels derived from the sensitivity analyses.

© 2023 Chen N et al. *JAMA Network Open.*

eTable 12. Health-care costs of musculoskeletal disorders attributable to high body mass index borne by sector and location in 2019

	The public sector		The private sector/third party		The out-of-pocket sector	
Location	Amount (uncertainty level, US$, thousands)	% of health-care costs	Amount (uncertainty level, US$, thousands)	% of health-care costs	Amount (uncertainty level, US$, thousands)	% of health-care costs
Global	35599675 (17845248 to 59621629)	58.9	14534104 (7763985 to 23215316)	24.0	10322440 (5057960 to 17570981)	17.1
Africa	237371 (124522 to 384181)	50.5	116234 (60552 to 191245)	24.7	115979 (56644 to 198030)	24.7
Algeria	66691 (33881 to 107651)	65.0	1539 (782 to 2484)	1.5	34269 (17410 to 55316)	33.4
Angola	1665 (681 to 3161)	41.2	861 (352 to 1634)	21.3	1515 (620 to 2877)	37.5
Benin	282 (136 to 490)	22.7	377 (182 to 655)	30.3	584 (282 to 1015)	47.0
Botswana	4446 (2390 to 7179)	78.5	1036 (557 to 1674)	18.3	176 (94 to 284)	3.1
Burkina Faso	988 (407 to 1893)	41.8	556 (229 to 1064)	23.5	821 (338 to 1572)	34.7
Burundi	135 (47 to 288)	33.4	170 (59 to 362)	42.0	100 (35 to 213)	24.7
Cabo Verde	315 (159 to 534)	65.8	45 (22 to 76)	9.3	119 (60 to 202)	24.9
Cameroon	199 (101 to 332)	3.4	1410 (717 to 2354)	24.1	4242 (2156 to 7081)	72.5
Central African Republic	15 (5 to 32)	10.6	41 (14 to 88)	29.1	85 (28 to 183)	60.3
Chad	100 (38 to 197)	17.3	147 (56 to 289)	25.4	331 (127 to 651)	57.2
Comoros	27 (12 to 50)	16.1	37 (16 to 68)	22.1	103 (45 to 191)	61.8
Congo	268 (134 to 454)	37.6	118 (59 to 199)	16.5	327 (164 to 554)	45.9
Democratic Republic of the Congo	355 (142 to 702)	15.8	1028 (412 to 2031)	45.7	866 (347 to 1711)	38.5

© 2023 Chen N et al. *JAMA Network Open.*

Equatorial Guinea	241 (127 to 400)	21.3	37 (20 to 62)	3.3	854 (452 to 1418)	75.5
Eritrea	28 (11 to 54)	17.6	62 (24 to 120)	39.2	68 (27 to 132)	43.2
Eswatini	737 (387 to 1174)	50.7	563 (296 to 896)	38.7	154 (81 to 246)	10.6
Ethiopia	937 (360 to 1880)	22.7	1627 (625 to 3263)	39.4	1565 (601 to 3139)	37.9
Gabon	1251 (631 to 2059)	60.3	345 (174 to 567)	16.6	479 (242 to 789)	23.1
Gambia	81 (38 to 144)	27.2	148 (69 to 262)	49.6	69 (32 to 123)	23.2
Ghana	4061 (2102 to 6751)	40.2	2384 (1234 to 3964)	23.6	3657 (1893 to 6080)	36.2
Guinea	292 (126 to 546)	22.5	238 (103 to 444)	18.3	769 (332 to 1435)	59.2
Guinea-Bissau	16 (6 to 30)	6.4	70 (28 to 134)	28.4	160 (65 to 308)	65.2
Ivory Coast	1498 (699 to 2653)	29.1	1730 (807 to 3063)	33.6	1920 (896 to 3401)	37.3
Kenya	6389 (3089 to 11190)	46.0	4125 (1995 to 7225)	29.7	3375 (1632 to 5911)	24.3
Lesotho	648 (331 to 1086)	43.5	636 (325 to 1066)	42.7	206 (105 to 345)	13.8
Liberia	175 (87 to 296)	16.1	320 (160 to 542)	29.5	591 (295 to 999)	54.4
Madagascar	366 (150 to 699)	32.2	401 (164 to 766)	35.3	369 (151 to 706)	32.5
Malawi	491 (201 to 935)	32.6	762 (312 to 1452)	50.6	254 (104 to 485)	16.9
Mali	431 (182 to 809)	33.6	449 (189 to 842)	35.0	403 (170 to 756)	31.4
Mauritania	572 (296 to 951)	37.5	267 (138 to 444)	17.5	686 (356 to 1141)	45.0
Mauritius	3128 (1476 to 5369)	47.0	479 (226 to 822)	7.2	3041 (1435 to 5220)	45.7
Mozambique	400 (168 to 757)	21.3	1292 (541 to 2443)	68.7	188 (79 to 356)	10.0
Namibia	1799 (956 to 3000)	46.9	1722 (915 to 2873)	44.9	314 (167 to 525)	8.2
Niger	310 (123 to 610)	35.7	158 (63 to 311)	18.2	400 (159 to 788)	46.1

© 2023 Chen N et al. *JAMA Network Open.*

Nigeria	7613 (3569 to 13540)	15.9	6464 (3030 to 11496)	13.5	33755 (15823 to 60034)	70.5
Rwanda	639 (248 to 1253)	39.9	775 (300 to 1520)	48.4	187 (73 to 367)	11.7
Sao Tome and Principe	37 (18 to 63)	47.3	27 (13 to 46)	35.1	14 (7 to 23)	17.7
Senegal	685 (322 to 1207)	25.0	655 (308 to 1154)	23.9	1397 (656 to 2462)	51.0
Seychelles	316 (151 to 544)	72.7	9 (4 to 15)	2.0	110 (52 to 189)	25.2
Sierra Leone	221 (88 to 428)	14.0	486 (194 to 943)	30.8	871 (348 to 1689)	55.2
South Africa	123442 (68087 to 193717)	58.8	74527 (41107 to 116955)	35.5	11966 (6600 to 18779)	5.7
South Sudan	120 (55 to 217)	16.3	445 (204 to 802)	60.2	174 (80 to 313)	23.5
Togo	124 (57 to 225)	15.1	153 (70 to 279)	18.7	542 (248 to 986)	66.2
Uganda	559 (239 to 1048)	15.1	1726 (738 to 3233)	46.6	1418 (606 to 2657)	38.3
United Republic of Tanzania	2374 (1127 to 4167)	40.9	2142 (1017 to 3759)	36.9	1288 (612 to 2262)	22.2
Zambia	1276 (582 to 2311)	40.1	1582 (722 to 2864)	49.7	325 (148 to 588)	10.2
Zimbabwe	627 (298 to 1105)	17.6	2066 (980 to 3643)	58.0	869 (412 to 1532)	24.4
The Americas	17725102 (9614909 to 27937145)	51.6	12176301 (6661181 to 19073842)	35.4	4443241 (2389295 to 7047604)	12.9
Antigua and Barbuda	238 (116 to 401)	58.4	70 (34 to 118)	17.2	99 (48 to 167)	24.3
Argentina	233320 (109572 to 406847)	62.4	37391 (17560 to 65200)	10.0	103573 (48640 to 180603)	27.7
Bahamas	1933 (980 to 3163)	52.0	810 (411 to 1326)	21.8	974 (494 to 1594)	26.2
Barbados	763 (378 to 1255)	44.8	145 (72 to 238)	8.5	795 (394 to 1308)	46.7
Belize	372 (190 to 594)	69.9	44 (23 to 71)	8.3	116 (59 to 185)	21.8

© 2023 Chen N et al. *JAMA Network Open.*

Bolivia (Plurinational State of)	13388 (6690 to 21993)	71.2	921 (460 to 1514)	4.9	4494 (2246 to 7383)	23.9
Brazil	499731 (260311 to 819154)	40.7	421149 (219378 to 690343)	34.3	305732 (159257 to 501153)	24.9
Canada	752100 (368524 to 1283272)	70.2	159634 (78220 to 272375)	14.9	159634 (78220 to 272375)	14.9
Chile	112195 (54469 to 190271)	50.9	35929 (17443 to 60931)	16.3	72299 (35100 to 122611)	32.8
Colombia	148741 (71822 to 250814)	71.9	27514 (13286 to 46395)	13.3	30824 (14884 to 51977)	14.9
Costa Rica	22850 (10907 to 38523)	72.5	1607 (767 to 2710)	5.1	7028 (3355 to 11849)	22.3
Cuba	154328 (76982 to 254368)	89.3	173 (86 to 285)	0.1	18319 (9138 to 30194)	10.6
Dominica	138 (70 to 224)	64.5	4 (2 to 6)	1.7	73 (37 to 118)	33.9
Dominican Republic	14483 (6734 to 24782)	44.9	3903 (1815 to 6678)	12.1	13838 (6434 to 23678)	42.9
Ecuador	37840 (20556 to 57859)	61.8	4470 (2428 to 6834)	7.3	18920 (10278 to 28929)	30.9
El Salvador	9110 (4263 to 15411)	63.5	1162 (544 to 1966)	8.1	4075 (1907 to 6893)	28.4
Grenada	129 (65 to 216)	41.4	13 (7 to 22)	4.2	170 (85 to 284)	54.4
Guatemala	7058 (3141 to 12703)	38.4	1029 (458 to 1852)	5.6	10293 (4581 to 18525)	56.0
Guyana	794 (379 to 1354)	59.4	76 (36 to 130)	5.7	465 (222 to 793)	34.8
Haiti	124 (46 to 250)	11.0	515 (192 to 1038)	45.7	488 (182 to 984)	43.3
Honduras	3127 (1394 to 5517)	39.2	654 (292 to 1154)	8.2	4196 (1871 to 7402)	52.6
Jamaica	5058 (2630 to 8176)	65.3	1417 (737 to 2291)	18.3	1270 (661 to 2053)	16.4

© 2023 Chen N et al. *JAMA Network Open.*

Mexico	258109 (128325 to 427556)	49.3	45025 (22385 to 74584)	8.6	220414 (109584 to 365113)	42.1
Nicaragua	4696 (2191 to 8059)	60.9	355 (166 to 609)	4.6	2653 (1238 to 4552)	34.4
Panama	19496 (9362 to 32891)	66.1	1858 (892 to 3135)	6.3	8141 (3909 to 13734)	27.6
Paraguay	8434 (4185 to 14187)	46.0	2273 (1128 to 3824)	12.4	7627 (3784 to 12830)	41.6
Peru	46078 (21720 to 77272)	62.9	6593 (3108 to 11056)	9.0	20585 (9703 to 34520)	28.1
Saint Kitts and Nevis	172 (86 to 285)	49.3	15 (8 to 25)	4.4	162 (81 to 268)	46.4
Saint Lucia	259 (127 to 427)	47.4	34 (17 to 57)	6.3	253 (124 to 418)	46.3
Saint Vincent and the Grenadines	177 (85 to 296)	66.3	12 (6 to 21)	4.6	78 (37 to 130)	29.1
Suriname	2070 (989 to 3444)	72.0	342 (163 to 569)	11.9	463 (221 to 770)	16.1
Trinidad and Tobago	5978 (3113 to 9578)	46.0	923 (481 to 1478)	7.1	6095 (3174 to 9765)	46.9
United States of America	15329517 (8429217 to 23910391)	50.8	11406609 (6272134 to 17791590)	37.8	3409912 (1875003 to 5318650)	11.3
Uruguay	22452 (10646 to 38776)	66.6	6034 (2861 to 10422)	17.9	5225 (2478 to 9024)	15.5
Venezuela (Bolivarian Republic of)	9841 (4643 to 16835)	46.0	7595 (3583 to 12992)	35.5	3958 (1867 to 6771)	18.5
Eastern Mediterranean	795303 (423113 to 1270111)	53.9	191550 (102210 to 306277)	13.0	488393 (253645 to 792639)	33.1
Afghanistan	660 (313 to 1160)	8.2	1006 (478 to 1768)	12.5	6385 (3029 to 11216)	79.3
Bahrain	8399 (4395 to 13319)	59.2	1575 (824 to 2497)	11.1	4214 (2205 to 6682)	29.7
Djibouti	51 (21 to 97)	53.7	21 (9 to 40)	22.2	23 (9 to 44)	24.2
Egypt	53529 (28266 to 84496)	27.8	18292 (9659 to 28875)	9.5	120729 (63751 to 190572)	62.7

© 2023 Chen N et al. *JAMA Network Open.*

Iran (Islamic Republic of)	145399 (76192 to 240113)	49.5	32311 (16932 to 53358)	11.0	116025 (60800 to 191605)	39.5
Iraq	20811 (10431 to 33624)	49.4	211 (106 to 340)	0.5	21106 (10579 to 34101)	50.1
Jordan	14270 (7762 to 22106)	51.2	5156 (2805 to 7988)	18.5	8445 (4594 to 13082)	30.3
Kuwait	45605 (25464 to 69537)	87.0	629 (351 to 959)	1.2	6186 (3454 to 9431)	11.8
Lebanon	15568 (7993 to 24952)	49.0	5560 (2854 to 8911)	17.5	10643 (5464 to 17059)	33.5
Libya	17993 (9517 to 28680)	63.3	0 (0 to 0)	0.0	10432 (5518 to 16628)	36.7
Morocco	19157 (9430 to 32133)	39.9	6386 (3143 to 10711)	13.3	22469 (11060 to 37689)	46.8
Oman	13836 (7275 to 22156)	86.4	1121 (589 to 1795)	7.0	1057 (556 to 1692)	6.6
Pakistan	10140 (4299 to 19243)	32.0	4500 (1907 to 8539)	14.2	17048 (7227 to 32353)	53.8
Qatar	24489 (13412 to 37711)	72.8	5012 (2745 to 7718)	14.9	4138 (2266 to 6372)	12.3
Saudi Arabia	289548 (157047 to 455417)	69.2	59834 (32453 to 94111)	14.3	69040 (37446 to 108589)	16.5
Sudan	3863 (1912 to 6526)	22.7	1702 (842 to 2875)	10.0	11470 (5676 to 19376)	67.4
Syrian Arab Republic	21721 (10728 to 35778)	45.3	479 (237 to 790)	1.0	25749 (12718 to 42412)	53.7
Tunisia	22296 (11096 to 36535)	57.1	1952 (972 to 3199)	5.0	14799 (7365 to 24250)	37.9
United Arab Emirates	67685 (37437 to 106007)	52.3	45554 (25196 to 71347)	35.2	16177 (8948 to 25336)	12.5
Yemen	285 (124 to 522)	10.2	248 (108 to 456)	8.9	2260 (981 to 4149)	81.0

© 2023 Chen N et al. *JAMA Network Open.*

Europe	11838002 (5823755 to 20086348)	72.6	1361900 (663331 to 2326912)	8.3	3118476 (1538070 to 5290822)	19.1
Albania	5350 (2611 to 9086)	54.0	139 (68 to 236)	1.4	4418 (2156 to 7504)	44.6
Andorra	960 (454 to 1670)	69.5	240 (114 to 418)	17.4	181 (86 to 315)	13.1
Armenia	2074 (1071 to 3424)	12.4	468 (242 to 773)	2.8	14184 (7325 to 23416)	84.8
Austria	195079 (93364 to 342588)	73.0	21111 (10104 to 37075)	7.9	51041 (24428 to 89636)	19.1
Azerbaijan	5476 (2717 to 9197)	31.7	52 (26 to 87)	0.3	11747 (5828 to 19729)	68.0
Belarus	40332 (20733 to 66808)	70.4	2234 (1149 to 3701)	3.9	14723 (7569 to 24389)	25.7
Belgium	243744 (117139 to 426250)	76.8	16186 (7779 to 28306)	5.1	57762 (27759 to 101012)	18.2
Bosnia and Herzegovina	16220 (8129 to 27243)	68.7	449 (225 to 753)	1.9	6941 (3479 to 11658)	29.4
Bulgaria	46646 (23804 to 76511)	59.2	1497 (764 to 2456)	1.9	30730 (15681 to 50404)	39.0
Croatia	43252 (22337 to 70226)	81.5	3715 (1919 to 6032)	7.0	6103 (3152 to 9909)	11.5
Cyprus	4967 (2233 to 8982)	56.0	1188 (534 to 2149)	13.4	2714 (1220 to 4908)	30.6
Czechia	179388 (92824 to 288236)	81.5	9685 (5011 to 15561)	4.4	31255 (16173 to 50220)	14.2
Denmark	148156 (71314 to 258766)	83.3	4446 (2140 to 7766)	2.5	25256 (12157 to 44111)	14.2
Estonia	16098 (8664 to 25921)	74.4	325 (175 to 523)	1.5	5193 (2795 to 8362)	24.0
Finland	113304 (54891 to 193722)	80.2	3391 (1643 to 5797)	2.4	24582 (11909 to 42030)	17.4

© 2023 Chen N et al. *JAMA Network Open.*

France	1404298 (688866 to 2394688)	75.3	287200 (140884 to 489750)	15.4	173439 (85079 to 295758)	9.3
Georgia	6410 (3218 to 10997)	40.8	1948 (978 to 3342)	12.4	7353 (3691 to 12615)	46.8
Germany	2720028 (1360562 to 4559515)	77.7	332565 (166349 to 557470)	9.5	448087 (224134 to 751117)	12.8
Greece	73667 (35145 to 125609)	48.1	25577 (12202 to 43611)	16.7	53910 (25720 to 91922)	35.2
Hungary	100095 (53800 to 159783)	68.0	5741 (3086 to 9164)	3.9	41510 (22311 to 66263)	28.2
Iceland	7478 (3590 to 12879)	82.9	144 (69 to 249)	1.6	1398 (671 to 2408)	15.5
Ireland	115004 (56611 to 195305)	74.6	21120 (10396 to 35867)	13.7	18037 (8879 to 30631)	11.7
Israel	69792 (33052 to 121520)	64.8	15402 (7294 to 26817)	14.3	22618 (10711 to 39381)	21.0
Italy	912458 (424145 to 1617975)	73.9	34572 (16070 to 61304)	2.8	287690 (133729 to 510133)	23.3
Kazakhstan	31453 (16328 to 51328)	59.9	3256 (1690 to 5313)	6.2	17801 (9241 to 29049)	33.9
Kyrgyzstan	2192 (1050 to 3820)	51.4	102 (49 to 178)	2.4	1970 (944 to 3434)	46.2
Latvia	14974 (7851 to 24493)	60.6	939 (492 to 1536)	3.8	8822 (4625 to 14429)	35.7
Lithuania	28167 (14102 to 46506)	65.1	1168 (585 to 1929)	2.7	13976 (6997 to 23074)	32.3
Luxembourg	15507 (7533 to 26742)	85.9	812 (395 to 1401)	4.5	1733 (842 to 2989)	9.6
Malta	7015 (3301 to 12332)	63.1	245 (115 to 430)	2.2	3847 (1810 to 6762)	34.6
Monaco	834 (407 to 1406)	85.0	70 (34 to 117)	7.1	79 (38 to 132)	8.0
Montenegro	3894 (2036 to 6255)	60.8	38 (20 to 62)	0.6	2472 (1293 to 3971)	38.6

© 2023 Chen N et al. *JAMA Network Open.*

Netherlands	348395 (161661 to 608498)	65.9	124238 (57648 to 216991)	23.5	56039 (26003 to 97877)	10.6
North Macedonia	6944 (3549 to 11412)	59.0	71 (36 to 116)	0.6	4755 (2430 to 7814)	40.4
Norway	126842 (58692 to 225314)	85.8	444 (205 to 788)	0.3	20549 (9508 to 36502)	13.9
Poland	370034 (195382 to 593931)	71.4	42497 (22439 to 68211)	8.2	105724 (55823 to 169695)	20.4
Portugal	119964 (55317 to 213481)	60.9	17138 (7902 to 30497)	8.7	60081 (27704 to 106916)	30.5
Republic of Moldova	7448 (3805 to 12273)	59.7	574 (293 to 946)	4.6	4454 (2275 to 7339)	35.7
Romania	170731 (94493 to 270690)	80.1	2131 (1180 to 3379)	1.0	40285 (22296 to 63871)	18.9
Russian Federation	756238 (385822 to 1261318)	61.2	28421 (14500 to 47402)	2.3	452260 (230736 to 754318)	36.6
San Marino	713 (349 to 1210)	82.2	7 (3 to 12)	0.8	147 (72 to 250)	17.0
Serbia	38584 (19410 to 62272)	58.4	3039 (1529 to 4905)	4.6	24445 (12298 to 39453)	37.0
Slovakia	56500 (29379 to 91778)	78.8	1434 (746 to 2329)	2.0	13767 (7158 to 22362)	19.2
Slovenia	32257 (16802 to 51850)	72.4	7129 (3713 to 11458)	16.0	5213 (2715 to 8379)	11.7
Spain	702595 (313516 to 1295535)	70.6	75633 (33750 to 139463)	7.6	216949 (96808 to 400038)	21.8
Sweden	244237 (116403 to 420244)	84.9	3452 (1645 to 5940)	1.2	39987 (19058 to 68803)	13.9
Switzerland	114061 (53327 to 200444)	32.1	151371 (70770 to 266010)	42.6	89899 (42030 to 157982)	25.3
Tajikistan	586 (245 to 1131)	27.3	30 (13 to 58)	1.4	1528 (638 to 2951)	71.2

© 2023 Chen N et al. *JAMA Network Open.*

Turkey	412102 (211949 to 654213)	77.9	27509 (14148 to 43670)	5.2	89403 (45981 to 141928)	16.9
Turkmenistan	3059 (1586 to 5034)	18.0	884 (458 to 1454)	5.2	13050 (6767 to 21478)	76.8
Ukraine	106439 (56438 to 172702)	44.8	9741 (5165 to 15805)	4.1	121406 (64375 to 196989)	51.1
United Kingdom	1634932 (806325 to 2734462)	79.5	69922 (34484 to 116946)	3.4	351665 (173436 to 588167)	17.1
Uzbekistan	11031 (5425 to 18773)	41.6	212 (104 to 361)	0.8	15300 (7525 to 26039)	57.7
Southeast Asia	364662 (164581 to 655243)	43.5	120119 (54178 to 216103)	14.3	353402 (158103 to 639230)	42.2
Bangladesh	3488 (1338 to 6978)	18.6	1631 (626 to 3264)	8.7	13631 (5228 to 27274)	72.7
Bhutan	295 (126 to 541)	73.6	35 (15 to 64)	8.7	71 (30 to 131)	17.8
India	143912 (64651 to 259259)	32.8	54406 (24441 to 98013)	12.4	240438 (108014 to 433152)	54.8
Indonesia	80731 (37842 to 142957)	48.9	26910 (12614 to 47652)	16.3	57453 (26931 to 101736)	34.8
Maldives	1114 (480 to 2085)	79.3	56 (24 to 105)	4.0	232 (100 to 434)	16.5
Myanmar	2490 (1020 to 4748)	15.8	1308 (536 to 2494)	8.3	11976 (4904 to 22837)	76.0
Nepal	1692 (669 to 3290)	24.8	1180 (467 to 2295)	17.3	3950 (1562 to 7681)	57.9
Sri Lanka	11555 (5019 to 20620)	47.2	1763 (766 to 3145)	7.2	11163 (4849 to 19921)	45.6
Thailand	119311 (53414 to 214593)	71.7	32781 (14676 to 58961)	19.7	14477 (6481 to 26038)	8.7
Timor-Leste	75 (22 to 172)	55.9	48 (14 to 110)	35.9	11 (3 to 25)	8.2

© 2023 Chen N et al. *JAMA Network Open.*

Western Pacific	4639235 (1694369 to 9288601)	66.2	568000 (222533 to 1100936)	8.1	1802949 (662203 to 3602656)	25.7
Australia	635477 (330076 to 1032682)	71.7	109015 (56624 to 177155)	12.3	141808 (73657 to 230445)	16.0
Brunei Darussalam	1160 (454 to 2260)	94.3	0 (0 to 0)	0.0	70 (27 to 137)	5.7
Cambodia	793 (295 to 1627)	24.3	369 (137 to 756)	11.3	2101 (782 to 4311)	64.4
China	1727697 (614068 to 3506298)	56.0	271495 (96496 to 550990)	8.8	1085981 (385985 to 2203959)	35.2
Cook Islands	111 (59 to 173)	87.6	8 (4 to 12)	6.0	8 (4 to 13)	6.4
Fiji	1162 (599 to 1864)	65.4	380 (196 to 610)	21.4	235 (121 to 376)	13.2
Japan	1661297 (506338 to 3569775)	83.9	63363 (19312 to 136154)	3.2	255432 (77852 to 548869)	12.9
Kiribati	58 (26 to 101)	82.3	12 (6 to 22)	17.6	0.070 (0.032 to 0.123)	0.1
Lao People's Democratic Republic	567 (237 to 1054)	36.9	326 (136 to 605)	21.2	642 (269 to 1193)	41.8
Malaysia	46240 (23188 to 78050)	52.2	11693 (5864 to 19737)	13.2	30649 (15370 to 51734)	34.6
Marshall Islands	23 (10 to 43)	41.2	27 (11 to 49)	47.1	7 (3 to 12)	11.7
Micronesia (Federated States of)	12 (6 to 20)	28.3	29 (14 to 48)	68.8	1 (1 to 2)	2.9
Mongolia	2165 (1024 to 3827)	56.6	329 (156 to 582)	8.6	1331 (630 to 2353)	34.8
Nauru	28 (13 to 47)	86.3	4 (2 to 7)	12.5	0.351 (0.169 to 0.595)	1.1
New Zealand	91150 (46112 to 151829)	75.6	14709 (7441 to 24502)	12.2	14709 (7441 to 24502)	12.2

© 2023 Chen N et al. *JAMA Network Open.*

Niue	8 (4 to 13)	98.7	0 (0 to 0)	0.0	0.104 (0.053 to 0.166)	1.3
Palau	84 (43 to 137)	45.0	81 (41 to 131)	43.1	22 (11 to 37)	12.0
Papua New Guinea	655 (239 to 1326)	57.7	368 (134 to 745)	32.4	112 (41 to 228)	9.9
Philippines	23818 (10530 to 42677)	40.6	6336 (2801 to 11352)	10.8	28511 (12605 to 51086)	48.6
Republic of Korea	379107 (132485 to 766476)	59.5	64990 (22712 to 131396)	10.2	192421 (67244 to 389035)	30.2
Samoa	158 (80 to 260)	72.6	38 (19 to 62)	17.2	22 (11 to 36)	10.1
Singapore	45157 (20335 to 81750)	50.2	17631 (7940 to 31919)	19.6	27166 (12234 to 49181)	30.2
Solomon Islands	88 (37 to 162)	74.7	26 (11 to 47)	21.8	4 (2 to 7)	3.4
Tonga	64 (34 to 102)	58.1	35 (19 to 56)	32.1	11 (6 to 17)	9.9
Tuvalu	21 (10 to 37)	74.3	7 (3 to 13)	25.4	0.086 (0.039 to 0.150)	0.3
Vanuatu	32 (15 to 57)	57.0	19 (9 to 34)	34.4	5 (2 to 9)	8.5
Viet Nam	22103 (8051 to 45954)	43.8	6712 (2445 to 13954)	13.3	21700 (7904 to 45114)	43.0

Values in parentheses represent uncertainty levels derived from the sensitivity analyses.

© 2023 Chen N et al. *JAMA Network Open.*

eTable 13. Health-care, morbidity-related, and total costs of musculoskeletal disorders attributable to high body mass index by World Bank income group in 2019

	Health-care costs		Morbidity-related costs		Total costs
	Amount (US$, millions)	% of total	Amount (US$, millions)	% of total	Amount (US$, millions)
Global	60485 (30683 to 100453)	33.5	120233 (53073 to 232665)	66.5	180718 (83757 to 333118)
High	49832 (25802 to 81534)	33.3	99684 (45114 to 189572)	66.7	149516 (70916 to 271106)
Upper-middle	9068 (4125 to 16180)	34.7	17074 (6574 to 35994)	65.3	26143 (10699 to 52174)
Lower-middle	1477 (706 to 2552)	30.6	3351 (1340 to 6837)	69.4	4829 (2046 to 9389)
Low	107 (50 to 187)	46.5	123 (46 to 262)	53.5	230 (96 to 449)

Values in parentheses represent uncertainty levels derived from the sensitivity analyses. The percentage calculations were based on the mean values.

© 2023 Chen N et al. *JAMA Network Open.*

eReferences

1. Institute for Health Metrics and Evaluation. GBD results tool. Accessed April 15, 2022. https://ghdx.healthdata.org/gbd-results-tool

2. Cieza A, Causey K, Kamenov K, Hanson SW, Chatterji S, Vos T. Global estimates of the need for rehabilitation based on the Global Burden of Disease Study 2019: a systematic analysis for the Global Burden of Disease Study 2019. *Lancet.* 2021;396(10267):2006-2017. doi:10.1016/s0140-6736(20)32340-0

3. GBD 2019 Diseases and Injuries Collaborators. Global burden of 369 diseases and injuries in 204 countries and territories, 1990-2019: a systematic analysis for the Global Burden of Disease Study 2019. *Lancet.* 2020;396(10258):1204-1222. doi:10.1016/s0140-6736(20)30925-9

4. World Health Organization. WHO global health expenditure database. Accessed December 20, 2021. https://apps.who.int/nha/database/Select/Indicators/en

5. The World Bank Group. World development indicators. Accessed December 25, 2021. https://databank.worldbank.org/source/world-development-indicators

6. International Labour Office Department of Statistics. Labour force by sex and age. Accessed March 10, 2022. https://www.ilo.org/shinyapps/bulkexplorer41/?lang=en&segment=indicator&id=EAP_TEAP_SEX_AGE_NB_A

7. United Nations Department of Economics and Social Affairs Population Dynamics. World population prospects 2019. Accessed October 15, 2021. https://population.un.org/wpp/Download/Standard/Population/

8. International Labour Office Department of Statistics. Statistics on the working-age population and labour force. Accessed April 4, 2022. https://ilostat.ilo.org/topics/population-and-labour-force/#

9. Statistics Niue. Population. Accessed September 16, 2022. https://niue.prism.spc.int/category/population/

10. International Labour Office Department of Statistics. Statistics on labour income and inequality. Accessed March 2, 2022. https://ilostat.ilo.org/topics/labour-income/

11. Moher D, Liberati A, Tetzlaff J, Altman DG. Preferred reporting items for systematic reviews and meta-analyses: the PRISMA statement. *PLoS Med.* 2009;6(7):e1000097. doi:10.1371/journal.pmed.1000097

12. Dieleman JL, Cao J, Chapin A, et al. US health care spending by payer and health condition, 1996-2016. *JAMA.* 2020;323(9):863-884. doi:10.1001/jama.2020.0734

13. The Economist Intelligence Unit. Breakaway: The global burden of cancer—challenges and opportunities. Accessed November 1, 2022. https://graphics.eiu.com/upload/eb/EIU_LIVESTRONG_Global_Cancer_Burden.pdf

14. World Economic Forum and the Harvard School of Public Health. The global economic burden of non-communicable diseases. Accessed March 5, 2022. https://www3.weforum.org/docs/WEF_Harvard_HE_GlobalEconomicBurdenNonCommunicableDiseases_2011.pdf

15. Ding D, Lawson KD, Kolbe-Alexander TL, et al. The economic burden of physical inactivity: a global analysis of major non-communicable diseases. *Lancet.*

© 2023 Chen N et al. *JAMA Network Open.*

2016;388(10051):1311-1324. doi:10.1016/s0140-6736(16)30383-x
16. Dieleman JL, Baral R, Birger M, et al. US spending on personal health care and public health, 1996-2013. *JAMA.* 2016;316(24):2627-2646. doi:10.1001/jama.2016.16885
17. Ebata-Kogure N, Murakami A, Nozawa K, et al. Treatment and healthcare cost among patients with hip or knee osteoarthritis: a cross-sectional study using a real-world claims database in Japan between 2013 and 2019. *Clin Drug Investig.* 2020;40(11):1071-1084. doi:10.1007/s40261-020-00968-6
18. Lee YR, Cho B, Jo MW, et al. Measuring the economic burden of disease and injury in Korea, 2015. *J Korean Med Sci.* 2019;34(Suppl 1):e80. doi:10.3346/jkms.2019.34.e80
19. Olafsson G, Jonsson E, Fritzell P, Hägg O, Borgström F. Cost of low back pain: results from a national register study in Sweden. *Eur Spine J.* 2018;27(11):2875-2881. doi:10.1007/s00586-018-5742-6
20. Alonso-García M, Sarría-Santamera A. The economic and social burden of low back pain in Spain: a national assessment of the economic and social impact of low back pain in Spain. *Spine (Phila Pa 1976).* 2020;45(16):E1026-e1032. doi:10.1097/brs.0000000000003476
21. Zhao X, Shah D, Gandhi K, et al. Clinical, humanistic, and economic burden of osteoarthritis among noninstitutionalized adults in the United States. *Osteoarthritis Cartilage.* 2019;27(11):1618-1626. doi:10.1016/j.joca.2019.07.002
22. Fisher S, Bellinger DC, Cropper ML, et al. Air pollution and development in Africa: impacts on health, the economy, and human capital. *Lancet Planet Health.* 2021;5(10):e681-e688. doi:10.1016/s2542-5196(21)00201-1
23. Kanal D, Kornegay JT. Accounting for household production in the national accounts: an update, 1965–2017. Accessed February 16, 2022. https://apps.bea.gov/scb/2019/06-june/0619-household-production.htm
24. Global Burden of Disease 2020 Health Financing Collaborator Network. Tracking development assistance for health and for COVID-19: a review of development assistance, government, out-of-pocket, and other private spending on health for 204 countries and territories, 1990-2050. *Lancet.* 2021;398(10308):1317-1343. doi:10.1016/s0140-6736(21)01258-7
25. Health and economic impact of air pollution in the states of India: the Global Burden of Disease Study 2019. *Lancet Planet Health.* 2021;5(1):e25-e38. doi:10.1016/s2542-5196(20)30298-9
26. Ahn YJ, Shin JS, Lee J, et al. Evaluation of use and cost of medical care of common lumbar disorders in Korea: cross-sectional study of Korean health insurance review and assessment service national patient sample data. *BMJ Open.* 2016;6(9):e012432. doi:10.1136/bmjopen-2016-012432
27. Flores NM, Nuevo J, Klein AB, Baumgartner S, Morlock R. The economic burden of uncontrolled gout: how controlling gout reduces cost. *J Med Econ.* 2019;22(1):1-6. doi:10.1080/13696998.2018.1532904
28. Williams EM, Walker RJ, Faith T, Egede LE. The impact of arthritis and joint pain on individual healthcare expenditures: findings from the Medical Expenditure Panel

© 2023 Chen N et al. *JAMA Network Open.*

Survey (MEPS), 2011. *Arthritis Res Ther.* 2017;19(1):38. doi:10.1186/s13075-017-1230-3
29. Eriksson JK, Johansson K, Askling J, Neovius M. Costs for hospital care, drugs and lost work days in incident and prevalent rheumatoid arthritis: how large, and how are they distributed? *Ann Rheum Dis.* 2015;74(4):648-654. doi:10.1136/annrheumdis-2013-204080
30. Kalkan A, Hallert E, Bernfort L, Husberg M, Carlsson P. Costs of rheumatoid arthritis during the period 1990-2010: a register-based cost-of-illness study in Sweden. *Rheumatology (Oxford).* 2014;53(1):153-160. doi:10.1093/rheumatology/ket290
31. Huscher D, Mittendorf T, von Hinüber U, et al. Evolution of cost structures in rheumatoid arthritis over the past decade. *Ann Rheum Dis.* 2015;74(4):738-745. doi:10.1136/annrheumdis-2013-204311
32. Kinge JM, Sælensminde K, Dieleman J, Vollset SE, Norheim OF. Economic losses and burden of disease by medical conditions in Norway. *Health Policy.* 2017;121(6):691-698. doi:10.1016/j.healthpol.2017.03.020
33. GBD 2019 Risk Factors Collaborators. Global burden of 87 risk factors in 204 countries and territories, 1990-2019: a systematic analysis for the Global Burden of Disease Study 2019. *Lancet.* 2020;396(10258):1223-1249. doi:10.1016/s0140-6736(20)30752-2

© 2023 Chen N et al. *JAMA Network Open.*
