# Supplementary material for: Health and Economic Outcomes Associated With Musculoskeletal Disorders Attributable to High Body Mass Index in 192 Countries and Territories in 2019
Source: JAMA Netw Open. 2023 Jan 20;6(1):e2250674. doi: 10.1001/jamanetworkopen.2022.50674 (PMC9860530; doi:10.1001/jamanetworkopen.2022.50674)
Supplement: Supplement 2. — Data Sharing Statement [file jamanetwopen-e2250674-s002.pdf]

Data Sharing Statement

Chen. Health and Economic Outcomes Associated With Musculoskeletal Disorders Attributable to High Body Mass Index in 192 Countries and Territories in 2019. *JAMA Netw Open*. Published January 20, 2023. doi:10.1001/jamanetworkopen.2022.50674

Data
Data available: Yes
Data types: Deidentified participant data
How to access data: All data we used for this work are publicly available. We provided information in the reference list.
When available: With publication

Supporting Documents
Document types: None

Additional Information
Who can access the data: Researchers whose proposed use of the data has been approved.
Types of analyses: For the research purpose.
Mechanisms of data availability: After approval of a proposal.
